# Chromatin interaction neural network (ChINN): a machine learning-based method for predicting chromatin interactions from DNA sequences

**DOI:** 10.1186/s13059-021-02453-5

**Published:** 2021-08-16

**Authors:** Fan Cao, Yu Zhang, Yichao Cai, Sambhavi Animesh, Ying Zhang, Semih Can Akincilar, Yan Ping Loh, Xinya Li, Wee Joo Chng, Vinay Tergaonkar, Chee Keong Kwoh, Melissa J. Fullwood

**Affiliations:** 1 Cancer Science Institute of Singapore, National University of Singapore, 14 Medical Dr, Singapore, 117599 Singapore; 2 School of Computer Science and Engineering, Nanyang Technological University, Block N4, 50 Nanyang Avenue, Singapore, 639798 Singapore; 3 Institute of Molecular and Cell Biology, Agency for Science (IMCB), A*STAR (Agency for Science, Technology and Research,, Singapore, 138673 Singapore; 4 School of Biological Sciences, Nanyang Technological University, 60 Nanyang Drive, Singapore, 637551 Singapore; 5 Department of Medicine, Yong Loo Lin School of Medicine, National University of Singapore, 1E Kent Ridge Road, Singapore, 119228 Singapore; 6 Department of Haematology-Oncology, National University Cancer Institute, National University Health System, NUH Zone B, Medical Centre, Singapore, 119074 Singapore; 7 Department of Pathology, Yong Loo Lin School of Medicine, National University of Singapore (NUS), Singapore, 117597 Singapore

**Keywords:** Machine learning, 3D genome organization, Chromatin interactions, ChIA-PET, Hi-C, DNA sequence, Leukemia, Bioinformatics

## Abstract

Chromatin interactions play important roles in regulating gene expression. However, the availability of genome-wide chromatin interaction data is limited. We develop a computational method, chromatin interaction neural network (ChINN), to predict chromatin interactions between open chromatin regions using only DNA sequences. ChINN predicts CTCF- and RNA polymerase II-associated and Hi-C chromatin interactions. ChINN shows good across-sample performances and captures various sequence features for chromatin interaction prediction. We apply ChINN to 6 chronic lymphocytic leukemia (CLL) patient samples and a published cohort of 84 CLL open chromatin samples. Our results demonstrate extensive heterogeneity in chromatin interactions among CLL patient samples.

**Supplementary Information:**

The online version contains supplementary material available at 10.1186/s13059-021-02453-5.

## Introduction

Chromatin interactions play important roles in regulating gene expression [1–3]. They bridge enhancers to genes [4–6] and create insulated domains to constrain the reach of enhancers [7]. High-throughput experimental techniques such as high-throughput chromosome conformation capture (Hi-C) [8] and chromatin interaction analysis with paired-end tags (ChIA-PET) [9] have been developed to detect genome-wide chromatin interactions. These techniques greatly advanced the understanding of genome organization and its roles in transcription regulation [4, 10–12]. However, due to costs and technical challenges, these methods have not been widely applied to large cohorts of cell lines or clinical samples. Hence, our understanding of how common or rare chromatin interactions are in different patient samples is limited.

A predictor that uses DNA sequences to predict chromatin interactions could potentially expand our understanding of genome organization. Sophisticated computational methods such as DeepSea [13] and DeepBind [14] have demonstrated that many transcription factors binding sites in open chromatin regions could be predicted from DNA sequences. Additionally, various computational methods have been developed to predict chromatin interactions to complement the experimental techniques [15–21]. Many of these methods rely on using various functional genomics data, meaning the use of chromatin immunoprecipitation sequencing (ChIP-seq) data of transcription factors and histone modifications, open chromatin data, and transcription data [15, 17, 19, 21].

Methods such as RIPPLE [17], TargetFinder [19], and JEME [15] reported high performances in predicting enhancer-promoter interactions using supervised machine learning approaches. However, the reported performances were exaggerated by using cross-validation with random splitting of samples [22, 23]. The lack of effective machine learning approaches has motivated the field to develop new methods.

Recently, the convolutional neural network framework was adapted to predict Hi-C contact matrices from 1-dimentional sequence data in a method called “Akita” [24]. There are other methods that predict Hi-C-like data and chromatin interactions, namely DeepTACT [25], SEPT [26], and DeepC [27]. A detailed comparison and description of these methods is discussed in Additional file 1: Table S1.

CTCF-associated genome folding patterns can be observed in the prediction results of Akita, suggesting the importance of CTCF in regulating chromatin interactions. In addition, prediction results can recapture the differences in genome folding between a normal and genetically altered cell lines, indicating that machine learning framework can predict different genome folding profiles given different input DNA sequences.

However, there are several limitations to these methods. First, Akita and DeepC only performs predictions with limited sequence regions (in the case of Akita, this is 1 Mb), thus long-range chromatin interactions cannot be predicted and genome-wide chromatin interactions cannot be obtained with these methods. Second, it is unclear whether ChIA-PET data can be predicted, as DeepTACT predicts promoter capture Hi-C data [25], and Akita, DeepC, and SEPT are restricted to Hi-C data. Third, none of these methods have been tested for their abilities to predict chromatin interactions de novo in patient cancer samples.

To overcome these challenges, in this study, we investigated the possibility of utilizing DNA sequence features to predict chromatin interactions between open chromatin regions, regardless of distance between them. Our study has several advantages. First, we demonstrated that open chromatin interactions can be predicted accurately from functional genomic data at the resolutions of the experimental techniques.

Second, we then developed a novel method, called chromatin interaction neural network (ChINN) to predict open chromatin interactions from DNA sequences. This model has been developed for RNA Polymerase II (RNA Pol II) ChIA-PET interactions, CTCF ChIA-PET interactions, and Hi-C interactions, overcoming previous limitations in terms of data input. Moreover, ChINN is able to identify open chromatin interactions in a genome-wide manner, overcoming the limitations of previous methods which were restricted to specific genomic regions.

Third, we extensively validated our method. ChINN was able to identify convergent CTCF motifs, AP-1 transcription family member motifs such as FOS, and other transcription factors such as MYC as being important in predicting chromatin interactions. Moreover, we further applied our model to a set of 6 newly generated chronic lymphocytic leukemia samples, which showed patient-specific chromatin interactions. We were able to validate predicted interactions by Hi-C and 4C. The models were then applied to a cohort of previously published 84 chronic lymphocytic leukemia (CLL) samples [28]. Thus, we demonstrated the prediction power of our method in practice.

Fourth, we used ChINN to characterize the levels of open chromatin interaction heterogeneity in patient samples. While we found that many chromatin interactions are ubiquitous, we also found widespread evidence for patient-specific open chromatin interactions, and open chromatin interactions that were different in different subtypes of CLL.

Taken together, our results indicate both functional genomics models and ChINN can predict open chromatin interactions, and application of ChINN to cancer patient samples demonstrates widespread patient heterogeneity in chromatin interactions.

## Results

### Open chromatin interactions can be predicted from functional genomic features

**Fig. 1:**
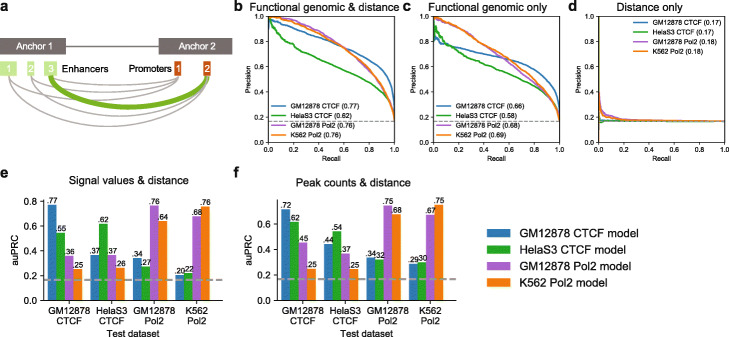
Performances of the functional genomic models on distance-matched datasets. The “Pol2” in the figure represents “RNA Pol II”. **a** Illustration of resolution discrepancy between cis-regulatory elements and chromatin interaction anchors. Precision-recall curves of the functional genomic models on distance-matched datasets using features based on **b** functional genomic data and distance (dis), **c** only functional genomic data, and **d** only distance. Numbers in brackets indicate the area-under precision-recall curve. **e**, **f**, Across-sample performances using distance (dis) and **e** signal values and **f** peak counts

In light of Xi et al. [22] and our previous study [23] showing that the existing prediction methods have exaggerated performances, we first tried to demonstrate that chromatin interactions could be predicted from functional genomic data. Many previous studies focused on enhancer-promoter interactions that were annotated using chromatin interactions derived from Hi-C or ChIA-PET [15, 17, 19]. The enhancers used were typically hundreds of base pairs, while the chromatin interaction anchors were much larger in size. For example, Hi-C anchors are normally 5 to 100 kb long (while only in rare case with extremely deep sequencing are the anchors down to 1 kb size) [29, 30], and ChIA-PET is normally several kilobase pairs long [31, 32]. The resolution discrepancy could lead to the introduction of a lot of noises to the training datasets (Fig. 1a). Thus, we used the chromatin interaction anchors directly.

Positive samples were constructed from ChIA-PET datasets separately and the corresponding distance-matched negative datasets were generated (Additional file 1: Fig. S1). The resulting distance-matched datasets have positive-to-negative ratios of approximately 1:5 and all chromatin interactions were between open chromatin regions in the corresponding cell types (Additional file 1: Table S2). We used ChIP-seq data of transcription factors and histone modifications commonly available to GM12878, K562, and HelaS3 and DNase-seq data from ENCODE [33] to annotate the anchors and build the feature vectors (Additional file 1: Table S3). For each chromatin interaction, the average signal of each transcription factor, histone modification, and open chromatin were calculated for both anchors. The distance between two anchors was also used as a feature.

Gradient boosted trees [34] were used to build models for each dataset. We tested three feature sets: (1) all common functional genomics data and distance, (2) distance only, and (3) common functional genomics data only. A precision-recall curve (PR) curve shows the trade-off between precision and recall across different decision thresholds. The auPRC is calculated as the area under the PR curve. The models trained on all features achieved auPRC ranging from 0.62 to 0.77 (Fig. 1b), while models trained on distance are mostly at baseline (Fig. 1d), showing that distance is properly controlled between positive and negative samples. The models trained on functional genomics features achieved auPRCs ranging from 0.58 to 0.69 (Fig. 1c), lower than models trained on all features. These results showed that although distance alone cannot predict chromatin interactions, combining distance feature with other features together can help to distinguish the positive and negative chromatin interaction considering the working mechanisms of the GB model (Additional file 1: Text S1).

The across-sample performances were lower than within-sample performances (Fig. 1e). Using peak counts instead of signal values produced better across-sample performances but lower within-sample performances (Fig. 1f). Models trained on RNA Pol II datasets generalize well to each other. Models trained on CTCF ChIA-PET datasets, however, did not generalize well to each other. Models trained on CTCF ChIA-PET data perform poorly on RNA Pol II ChIA-PET datasets and vice versa.

### Open chromatin interactions can be predicted from DNA sequences

In our previous section, we showed that open chromatin interactions can be predicted from functional genomics data, which consists of transcription factor data. As transcription factor binding can be predicted from sequences as shown by methods such as DeepSea (Zhou et al, Nature Methods, 2015) and DeepBind (Alipanahi et al, Nature Biotech, 2015), we reasoned that open chromatin interactions can be predicted from DNA sequences. Consequently, we went on to explore whether open chromatin interactions can be predicted from DNA sequences.

**Fig. 2:**
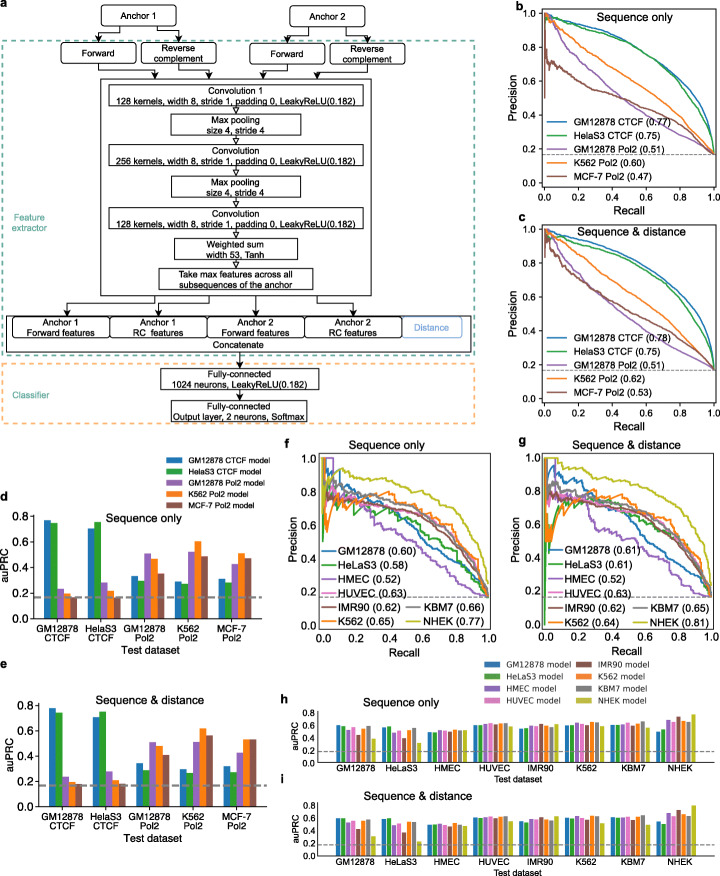
Architecture and performances of the ChINN sequence-based models on distance-matched datasets. The “Pol2” in the figure represents “RNA Pol II”. **a** The architecture of the sequence-based models using to train on distance-matched datasets. Precision-recall curves of the sequence-based models on distance-matched datasets using **b** only sequence features or **c** sequence features with distance. The numbers in the brackets indicates the area-under precision-recall curves. Across-sample performances as measured by area-under precision-recall curve (auPRC) of the models on distance-matched datasets using **d** only sequence features or **e** sequence features with distance. Precision-recall curves of the sequence-based models on distance-matched Hi-C datasets using **f** only sequence features or **g** sequence features with distance. The numbers in the brackets indicates the area-under precision-recall curves. Across-sample performances as measured by area-under precision-recall curve (auPRC) of the models on distance-matched Hi-C datasets using **h** only sequence features or **i** sequence features with distance

We built a convolutional neural network, ChINN, to predict chromatin interactions between open chromatin regions using DNA sequences (Fig. 2a). These models are called the ChINN sequence-based models. The models were trained on GM12878 CTCF, GM12878 RNA Pol II, HelaS3 CTCF, K562 RNA Pol II, and MCF-7 RNA Pol II datasets separately.

Compared to using functional genomics data for prediction, using sequences produced better within-sample performances for CTCF ChIA-PET datasets with auPRCs of 0.77 for GM12878 CTCF and 0.75 for HelaS3 CTCF (Fig. 2b), but worse within-sample performances for RNA Pol II ChIA-PET datasets with auPRC of 0.51 for GM12878 RNA Pol II, 0.6 for K562 RNA Pol II, and 0.47 for MCF-7 RNA Pol II. Including distance as a feature to classifier only slightly improved the performances for the distance-matched datasets (Fig. 2c). The across-sample performances of CTCF models showed well generalizability to each other (Fig. 2d). RNA Pol II models can also generalize to each other. Models trained on CTCF ChIA-PET datasets perform poorly on RNA Pol II ChIA-PET datasets and vice versa (Fig. 2d, e). The inability to generalize between CTCF chromatin interactions and RNA Pol II chromatin interactions could be attributed to the different sequence contexts.

For each model, we obtained and matched the position-weight matrices for all kernels on the first convolutional layer to known transcription factor binding motifs (Additional file 1: Fig. S2). As expected, CTCF motif was captured by both CTCF models. Other than the CTCF motif, the remaining known transcription factor binding motifs learned by the two models were different, indicating the possible cell-type-specific motifs. Our findings of the cell-type-specific motifs were supported by other pieces of evidence: studies show that cell-type-specific CTCF-mediated interactions are important in gene regulation [35, 36] and CTCF binding sites vary extensively across cell types [37, 38]. The patterns learned by RNA Pol II models showed more diversity and no matching transcription factor binding motif was shared among the three models. Interestingly, some of the transcription factors identified, such as ZNF143 in K562 and GATA3 in MCF-7, play important roles in the relevant cancer types [39, 40].

Besides, we also trained ChINN model on GM12878, HeLaS3, HMEC, HUVEC, IMR90, K562, KBM7, and NHEK Hi-C data, respectively. The auPRCs of within-sample performances using only sequences range from 0.52 to 0.77 for the above eight Hi-C models (Fig. 2f). Including distance as a feature to classifier only slightly improved the performances for the GM12878, HeLaS3, and NHEK Hi-C models (Fig. 2g). The across-sample performances of all eight Hi-C models showed well generalizability to each other (Fig. 2h, i).

Similarly, we obtained and matched the position-weight matrices for all kernels on the first convolutional layer to known transcription factor binding motifs for eight Hi-C datasets (Additional file 1: Table S4) and counted how many times each motif was detected (Additional file 1: Table S5). The CTCF motif was captured by all Hi-C models. The known transcription factor binding motifs learned by different Hi-C models were different. Some motifs, such as FOS, were learned by all models, but other motifs showed diversity, for example, ZN436 is detected by all other models except for HMEC, and ZIC3 is only detected by HeLaS3 (Additional file 1: Table S5). We noticed that the motifs detected in all cell lines exhibit smaller *p*-values than the cell-type-specific motifs, indicating that these “general” motifs are very important in predicting chromatin interactions. We speculate a model of chromatin interactions whereby there are general chromatin interactions facilitated by general transcription factors and common across different cell types, as well as cell-type specific chromatin interactions facilitated by cell-type specific transcription factors which can control cell-type specific transcription.

### Convergent CTCF motifs are important for prediction of CTCF-associated open chromatin interactions

After extracting the sequence features from both the forward and reverse complement sequences of the anchors, the sequence features were fed into the classifier to obtain a probability score that indicated how likely the pair of anchors were involved in a chromatin interaction. We obtained the feature importance scores of the gradient boosted trees trained and validated using a set of extended datasets that includes more negative samples than the distance-matched datasets (Methods, Additional file 1: Fig. S3a-d). We noted that the PR curves of the datasets that used sequence features and distance (Additional file 1: Fig. S3a) were better than that of sequence features alone (Additional file 1: Fig. S3b). However, distance alone was uninformative by itself in predicting chromatin interactions (Additional file 1: Fig. S3c), suggesting that it is the combination of distance as a property in addition to sequence features that provide predictive power.

**Fig. 3:**
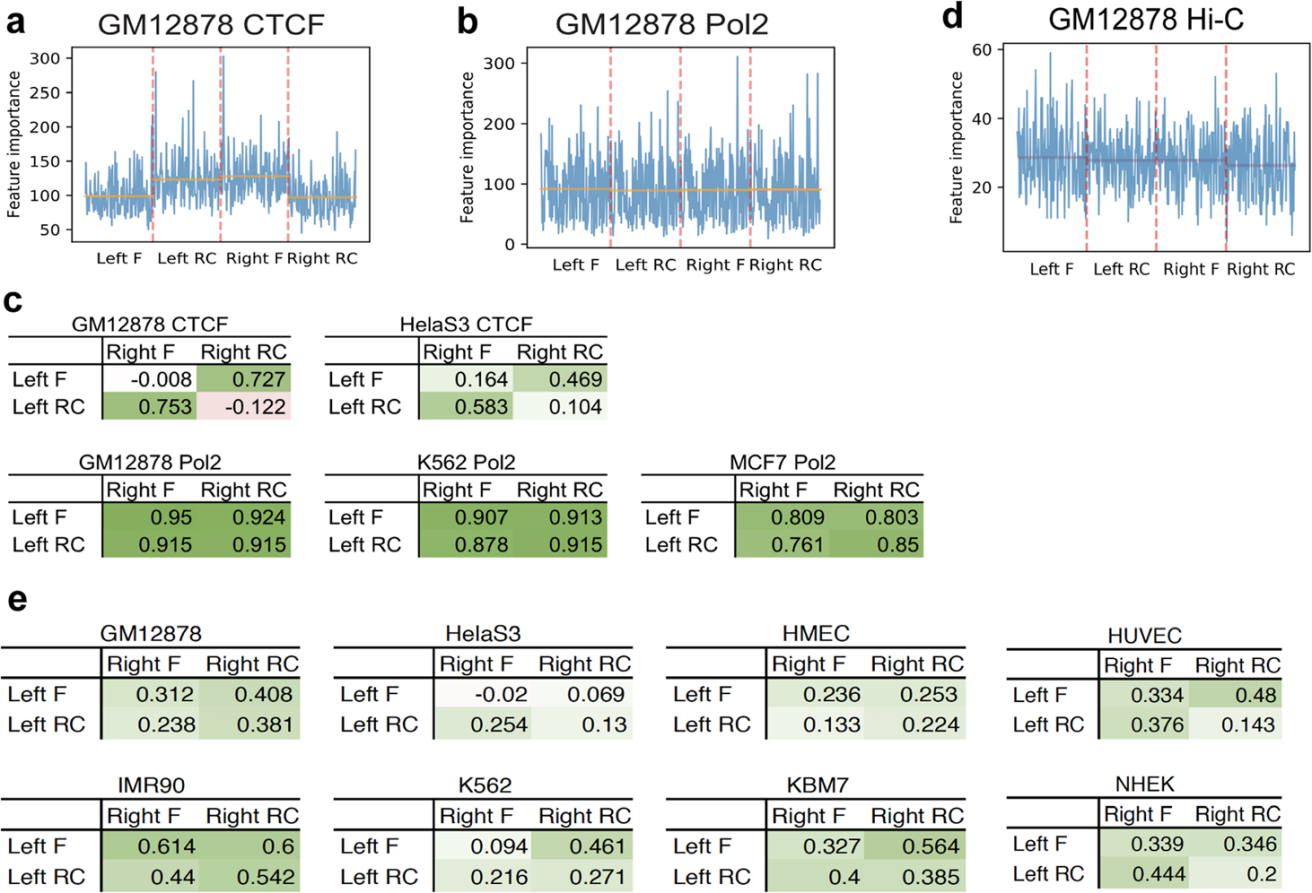
Sequence feature importance scores of gradient boosted trees trained on extended datasets. The “Pol2” in the figure represents “RNA Pol II”. **a**, **b** The importance scores of sequence features extracted from both directions (F, forward; RC, reverse complement) of the two anchors (left and right) by models trained on different datasets. The orange horizontal lines indicate average importance scores of the features from the strand of the anchor. **c** Pearson correlations between feature importance scores of the two anchors. **d** The importance scores of sequence features extracted from both directions (F, forward; RC, reverse complement) of the two anchors (left and right) by models trained on Hi-C datasets. The orange horizontal lines indicate average importance scores of the features from the strand of the anchor. **e** Pearson correlations between feature importance scores of the two anchors in Hi-C datasets

The sequence and distance-trained datasets were able to predict chromatin interactions across different cell types (Additional file 1: Fig. S3d). Consequently, we focused on the sequence features that were important for the prediction. As convergent CTCF motif has been observed in the anchor regions of CTCF loops [41–43], this suggests that the other sequence features or binding motifs at CTCF ChIA-PET anchors may also have such convergent orientation. Interestingly, in CTCF models the important sequence features were on different strands of the two anchors in a convergent manner (Fig. 3a, Additional file 1: Fig. S3e), while RNA Pol II models did not show such pattern (Fig. 3b, Additional file 1: Fig. S3f-g). For the CTCF models, importance scores of features on different strands of the two anchors showed good correlation, while importance scores of features on the same strand of the two anchors did not show much correlation (Fig. 3c). In contrast, the importance scores of features of RNA Pol II models were generally highly correlated regardless of the strand. These results are consistent with the previously observed convergent CTCF motifs at CTCF ChIA-PET and further suggest that other transcription factors also binds to CTCF loops in a similar manner.

The kernels on the last convolutional layer that generated the most important features in the extended CTCF models captured the CTCF motif (Additional file 1: Fig. S3h), suggesting that convergent CTCF motifs were important for the prediction of CTCF-associated chromatin interactions. However, using only CTCF motif information for the prediction of CTCF-associated open chromatin interactions could not recapitulate the performance achieved by the convolutional neural network (Additional file 1: Fig. S3i), indicating that CTCF was not the sole determining factor of chromatin interactions. We also showed the results when training with NN model (same NN structure as ChINN sequence-based models) on the same datasets using sequence and distance feature to illustrate the superiority of GB model here (Additional file 1: Fig. S3j).

Similarly, we trained gradient boosted trees with the corresponding extended datasets for eight Hi-C datasets. Distance was the largest contributor (in terms of feature importance score) when it was used together with sequence features (Additional file 1: Fig. S4a-d). But on its own, it was not very informative. This suggests its interaction with the sequence features is informative. When we visualized the sequence feature importance, although not as obvious as that of the CTCF models, we observed that the important sequence features were on different strands of the two anchors according to the corresponding mean values (Fig. 3d, Additional file 1: Fig. S4e). However, the importance scores of features did not show high correlation on Hi-C datasets (Fig. 3e). All the extended Hi-C models captured the CTCF motif via the kernels of the most important feature on the last convolutional layer (Additional file 1: Fig. S4f), indicating that convergent CTCF motifs were important for the prediction of Hi-C data chromatin interactions. The results trained with NN model using sequence and distance feature were also shown for reference (Additional file 1: Fig. S4g).

### Predicting chromatin interactions from open chromatin regions

The above models were trained and evaluated on known chromatin interactions. Without knowledge of chromatin interactions, as is the case for many clinical samples and cell types, the locations of the anchors would not be known. To be able to predict chromatin interactions between open chromatin regions, the models need to be able to predict chromatin interactions between paired genomic regions (anchors) of open chromatin regions.

**Fig. 4:**
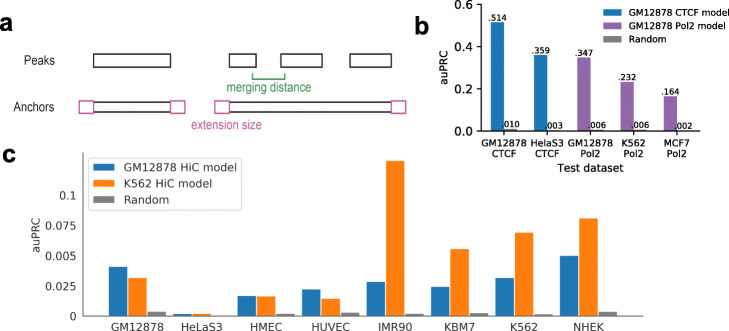
Performances of the final “from-open chromatin” models and validations. The “Pol2” in the figure represents “RNA Pol II”. **a** Illustration of the two parameters, merging distance and extension size, used in constructing putative chromatin interactions anchors from open chromatin regions. **b** Area-under precision-recall curves of the “from-open ChIA-PET chromatin” models. **c** Area-under precision-recall curves of the Hi-C “from-open chromatin” models

We tested different combinations of merging distances and extension sizes (Fig. 4a) based on validation datasets and determined that the merging distance of 3000 bp and extension size of 1000 bp for the construction of anchors in GM12878 cells (Additional file 1: Fig. S5a).

The pairs generated between anchors constructed from open chromatin regions in GM12878 were used to train gradient boosted trees for both CTCF and RNA Pol II models (see Methods). The positive-to-negative ratios were about 1:122 for CTCF chromatin interaction labeled samples and 1:186 for RNA Pol II chromatin interaction labeled samples. The CTCF model achieved within-sample auPRC of 0.514 and the RNA Pol II model achieved auPRC of 0.347 (Fig. 4b). In cross-sample evaluation, the CTCF model achieved auPRC of 0.359 on HelaS3 dataset and the RNA Pol II model achieved auPRCs of 0.232 and 0.164 on K562 and MCF-7 datasets, respectively (Fig. 4b). We were able to validate some of the predicted chromatin interactions in MCF-7 cells using 4C-seq (Additional file 1: Fig. S5b-d). Some of the validated chromatin interactions were not captured by the MCF-7 RNA Pol II ChIA-PET dataset, thus ChINN is able to identify bona fide chromatin interactions that might have been previously missed out due to insufficient sequence coverage.

We also generated pairs between anchors constructed from open chromatin regions in GM12878 and K562 Hi-C datasets with different combinations of merging distances and extension sizes (Additional file 1: Fig. S6a). We kept to the same parameters as the CTCF model, i.e., merging size of 3000 and extension size of 1000, to train gradient boosted trees due to the insignificant difference in auROC achieved by different parameters. The GM12878 and K562 Hi-C model had relatively low auPRC in the within-sample and cross-sample evaluation (Fig. 4c). However, we found that the auPRC of our ChINN method showed at least 4 times improvement over that of the random classifier. In cell line IMR90 tested by K562 model, ChINN showed as high as 57 times improvement. As there are a lot of data in the datasets (for example, the IMR90 dataset has 979,699 samples), these improvements in the auPRC could lead to many chromatin interactions being predicted correctly. Moreover, some of the predicted chromatin interactions in MCF-7 cells using 4C-seq were able to be validated by our Hi-C models (Additional file 1: Fig. S6b-d).

Other methods that predict Hi-C-like data and chromatin interactions are available, namely DeepTACT [25], SEPT [26], Akita [24], and DeepC [27]. However, except for SEPT, the other three machine learning methods are very different from ChINN in terms of the data. For example, DeepTACT uses promoter capture Hi-C input data, which is quite different from the use of Hi-C and ChIA-PET input data. For Akita and DeepC, their output consists of Hi-C contact matrices on lists of user-specified genomic regions, while our output is chromatin loops and probabilities of interaction across the whole genome. As a consequence, we cannot call loops from these partial Hi-C matrices of Akita and DeepC, because we would not know the background genomic interaction distribution. Therefore, direct comparison between ChINN output and Akita/DeepC output is not possible.

As for the SEPT, following its pipeline, we extended the input sequences or cut to 3 or 2 kb flanking regions from the center. But SEPT performs worse as compared with ChINN on our dataset (AUPRC = 0.0016 evaluated on K562 Hi-C test datasets with HeLaS3 as source data), as the sequences in our dataset are longer than these input sequences. Therefore, cutting the sequence to 3 or 2 kb according to what SEPT does would not let the model learn much useful information.

We concluded that each method is designed to investing ate different questions, and in Additional file 1: Fig. S7, we summarized the decision making process for researchers who wish to use the different methods. For example, if the researcher is interested in promoter-promoter or promoter-enhancer interactions identified by PCHi-C (Promoter Capture Hi-C), they should use DeepTACT. If the researcher is interested in an output that is shown as a Hi-C heatmap, they should use Akita. If the researcher is interested to look at general chromatin interactions predicted from Hi-C data, or RNA Pol II and CTCF chromatin interactions predicted from ChIA-PET data, they should use ChINN. ChINN is the only machine learning method currently available for predicting Hi-C and ChIA-PET chromatin interactions from sequences with outputs specified as open chromatin associated chromatin interactions instead of Hi-C matrices.

### Exploring chromatin interactions in patient samples

Next, we wished to apply our machine learning methods to patient samples to understand if our method could predict chromatin interactions in a completely new dataset. We obtained 6 chronic lymphocytic leukemia (CLL) patient samples. The clinical characteristics are described in Additional file 1: Table S6.

We prepared integrated Hi-C, ATAC-Seq, and RNA-Seq libraries from these 6 samples. We used Juicer to call topologically associated domains and loops from these patient samples. Our CLL samples showed many TADs and loops (Additional file 1: Table S7), thus indicating that we were able to perform Hi-C in these patient samples.

Next, we applied GM12878 and K562 Hi-C models to six new CLL samples. We used GM12878 and K562 Hi-C models for this prediction because CLL, GM12878, and K562 all come from hematopoietic lineages, and therefore GM12878 and K562 predictions would be likely to have captured both general chromatin interaction mechanisms and tissue-specific mechanisms that are relevant to hematopoietic cells.

**Fig. 5:**
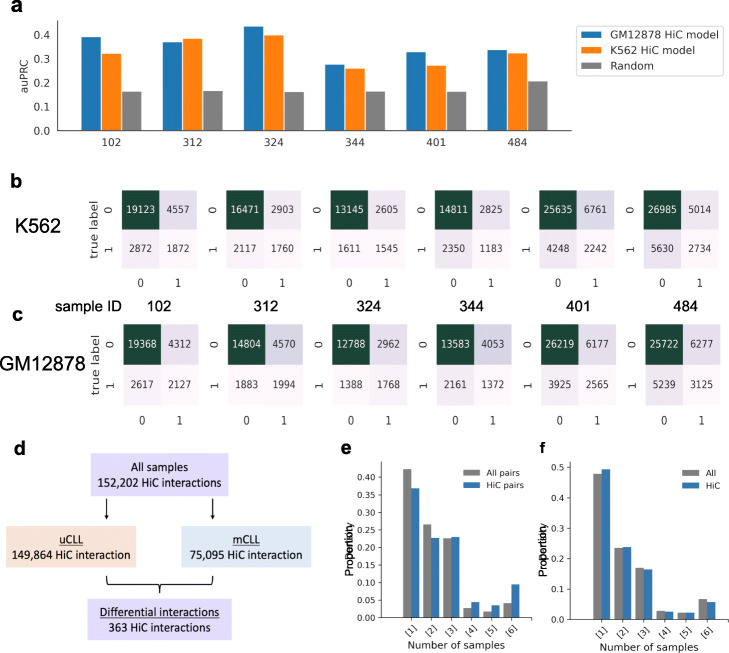
Applying Hi-C model on new CLL samples. **a** The auPRC values achieved by GM12878 and K562 Hi-C model, x-axis: new CLL samples. **b**, **c** The confusion matrices for 6 new CLL samples using K562 Hi-C model with threshold of 0.016 and GM12878 Hi-C model with threshold of 0.025. *x*-axis, true label; *y*-axis, predicted label; 0, negative; 1, positive. **d** Summary of the predicted chromatin interactions in the 6 new CLL samples and the differential chromatin interactions between uCLL and mCLL samples. **e** Conservation analysis of predicted chromatin interactions in new CLL samples. All pairs, all possible pairs used for prediction; *y*-axis, the proportion of total chromatin interactions that can be found in a particular number of samples. **f** Uniqueness analysis of open chromatin regions that overlap with Hi-C peaks from GM12878 cells in new CLL samples. All, all open chromatin regions; *y*-axis, the proportion of total chromatin interactions that can be found in a particular number of samples

The auPRC achieved by GM12878 Hi-C model range from 0.2772 to 0.4362, which are a bit higher than that of K562 Hi-C model, whose auPRC range from 0.2607 to 0.3996 (Fig. 5a). We calculated the *F*-score with different thresholds and finally determined the threshold of 0.025 for GM12878 model and 0.016 for K562 model to make the prediction on new CLL samples (Additional file 1: Fig. S8a-b), where the corresponding confusion matrix was shown as Fig. 5b and c.

One question we asked was whether there is patient heterogeneity in Hi-C data. We use “heterogeneity” to indicate that the chromatin interactions are different across patients. Clinical samples differ from each other due to a wide variety of factors including different driver mutations and different underlying genetics and epigenetics of each patient.

Here, we asked whether the subtype of the CLL samples could be one factor giving rise to patient heterogeneity. The CLL samples could be divided into two subtypes based on IGHV mutation status. In our data, two samples (102 and 344) are IGHV-unmutated CLL (uCLL) type and 4 samples (312, 324, 401, and 484) are IGHV-mutated CLL (mCLL) type. IGHV mutation status is an important prognostic biomarker in CLL, with mCLL being less aggressive [44].

Genomic sequences are almost identical across different patient samples, except for regions of patient-specific cancer structural variations and single nucleotide variations. Thus, if two anchors are identical in a different cell type, the probability that they are interacting given by the model will be the same. In this study, we have limited the scope of chromatin interaction prediction to only open chromatin regions, and call the predicted chromatin interactions “open chromatin interactions”.

Open chromatin profiles have been used to cluster cell types and cancer subtypes (Rendeiro et al, Nature Commun, 2016). With the assumption that the mechanisms of chromatin interactions are similar between different patient samples with the same cancer and with the patient-specific open chromatin regions, we explored the different chromatin interactions arising due to open chromatin differences between patient samples.

As a first step to investigate this question, we applied our ChINN framework on the six new CLL samples and built models using Hi-C and ATAC-seq data from each CLL sample. Models built using CLL samples would have captured general chromatin interaction mechanisms and tissue-specific mechanisms relevant to hematopoietic cells, as well as CLL-specific mechanisms.

Figure 5d showed the predicted chromatin interactions in 6 new CLL samples and the differences between uCLL and mCLL samples. With the selected threshold, a total of 152,202 Hi-C-associated open chromatin interactions were predicted (Fig. 5d) by GM12878 Hi-C model. We found extensive patient heterogeneity (Fig. 5e, f), as observed from the lack of similarity of chromatin interactions across the new CLL samples and the overlapping peaks between new CLL samples and GM12878 Hi-C peaks. For example, Fig. 5e indicates that 37% of Hi-C identified chromatin interactions can be found in only one sample, but not the other five samples. This indicates that many open chromatin interactions can only be found in one sample, and is an illustration of the level of heterogeneity in terms of the presence and absence of open chromatin regions and their associated chromatin interactions.

**Fig. 6:**
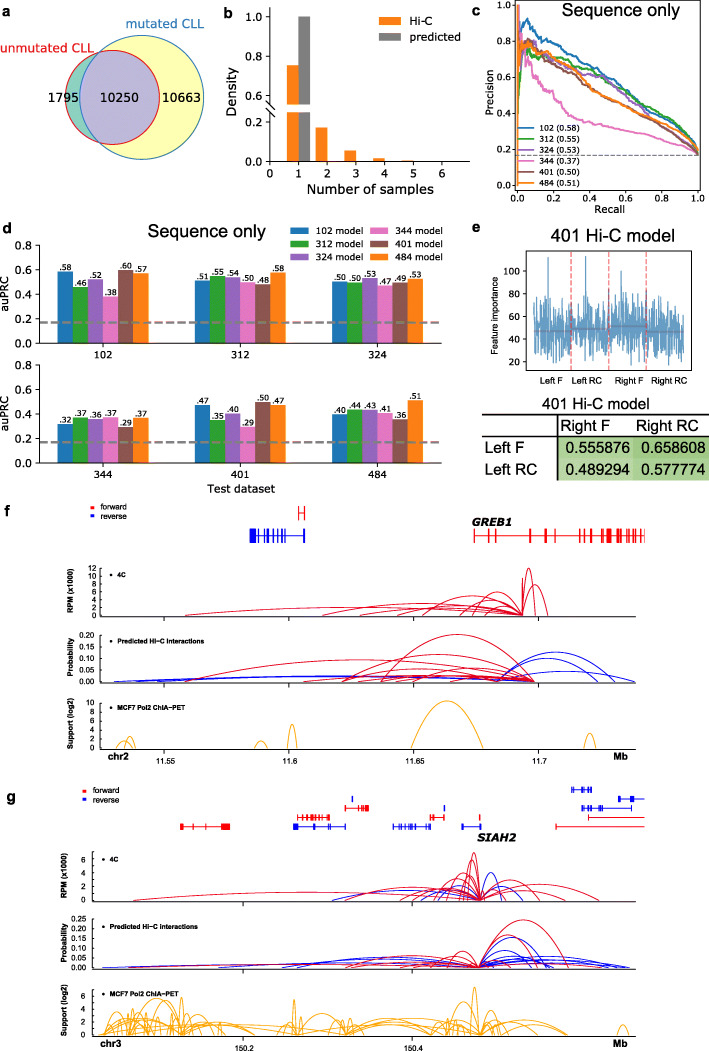
Performances of the sequence-based models in new CLL samples. **a** Venn diagram of chromatin interactions identified by Juicer in unmutated and mutated CLL samples. **b** Uniqueness analysis of real Hi-C and predicted Hi-C chromatin interactions in new CLL samples. Hi-C, real Hi-C interactions; predicted, predicted chromatin interactions using CLL 401 model. **c** Precision-recall curves of the sequence-based models on distance-matched Hi-C datasets using only sequence features. **d** Across-sample performances as measured by area-under precision-recall curve (auPRC) of the models on distance-matched Hi-C datasets using only sequence features. **e** The importance scores of sequence features extracted from both directions (F, forward; RC, reverse complement) of the two anchors (left and right) by models trained on CLL 401 sample. The orange horizontal lines indicate average importance scores of the features from the strand of the anchor. Pearson correlations between feature importance scores of the two anchors are given in the table. **f** Validations of predicted chromatin interactions by 4C-seq at *GREB1* gene region in MCF-7 cells. In the predicted Hi-C interaction panel, only those interactions connected to *GREB1* promoter were shown. **g** Validations of predicted chromatin interactions by 4C-seq at *SIAH2* gene region in MCF-7 cells. In the predicted Hi-C interaction panel, only those interactions connected to *SIAH2* promoter were shown

In addition, we also applied our ChINN framework on the six new CLL samples and built models using Hi-C and ATAC-seq data from each CLL sample. Our Hi-C libraries identified 1795 open chromatin interactions unique to uCLL samples and 10663 open chromatin interactions unique to mCLL samples (Fig. 6a). Moreover, uniqueness analysis of the Hi-C interactions from these six CLL samples similarly showed high patient heterogeneity (Fig. 6b). Thus, both predicted open chromatin interactions and Hi-C identified interactions indicate high patient heterogeneity.

These models have auPRC range from 0.37 to 0.58 (Fig. 6c). In addition, across-sample testing of these CLL models on other datasets from other CLL sample suggests a comparable performance (Fig. 6d). Inclusion of distance did not result in dramatic increase of the model performance (Additional file 1: Fig. S9a-9b). Moreover, the first convolutional layers of all CLL models were able to capture the CTCF and AP-1 transcription family member (FOS, JUN, JUNB, JUND) binding motif (Additional file 1: Fig. S9c), similar to the Hi-C models we showed earlier (Additional file 1: Fig. S4e; Additional file 1: Table S4-5).

After that, we trained gradient boosted trees with the corresponding extended datasets of the CLL samples. We observed that similar correlation of the important sequence features on different strands of the two anchors (Fig. 6e; Additional file 1: Fig. S9d-9e), although the within-sample and cross-sample auPRC were decreased (Additional file 1: Fig. S9f-9g).

We also generated open chromatin pairs using ATAC-seq to train the gradient boosted trees (merging size, 3000 bp; extension size, 1000 bp). Although the performances decreased compared with using Hi-C anchor region pairs as input, they were still higher than the random auPRC values (Additional file 1: Fig. S9h-9k). We further used the 401 CLL sample model to predict open chromatin interactions in MCF7 cells, as the 401 CLL model has the highest within-sample and across-sample performance. The predicted interactions correlate quite well with the real 4C-seq interactions (Fig. 6f, g, Additional file 1: Fig. S9l-9o, threshold = 0.016).

One question we asked was whether there is patient heterogeneity in Hi-C data. We first tried to associate the real and predicted Hi-C interactions with differentially expressed genes identified from RNA-seq data. The results showed that although the trend of different IFC scores (the fold change of the average number of open chromatin interactions observed at the gene promoter in uCLL samples over that in mCLL samples) could be observed, these differences were not significant (Additional file 1: Fig. S9p-9q). We also observed that the Hi-C interactions and ATAC-seq peaks in the new CLL samples showed high patient heterogeneity (Additional file 1: Fig. S9r). These patient heterogeneities may be a reason for the limited sample size in the IFC score analysis after we collapsed all six samples into mutated and unmutated categories (Additional file 1: Fig. S9p-9q).

Taken together, our results demonstrate across-sample prediction capability for the ChINN model. In addition, we observed high patient heterogeneity in the new CLL samples from the predicted open chromatin interactions as well as the Hi-C identified chromatin interactions.

### Exploring open chromatin interactions in a cohort of patient samples

Next, we used our machine learning method to predict open chromatin interactions in a cohort of patient samples and then analyzed the data. We applied the above models to 84 chronic lymphocytic leukemia (CLL) samples whose open chromatin profiles were available by ATAC-seq [28]. Among 84 CLL samples, 34 of them are uCLL type and 50 of them are mCLL type.

**Fig. 7:**
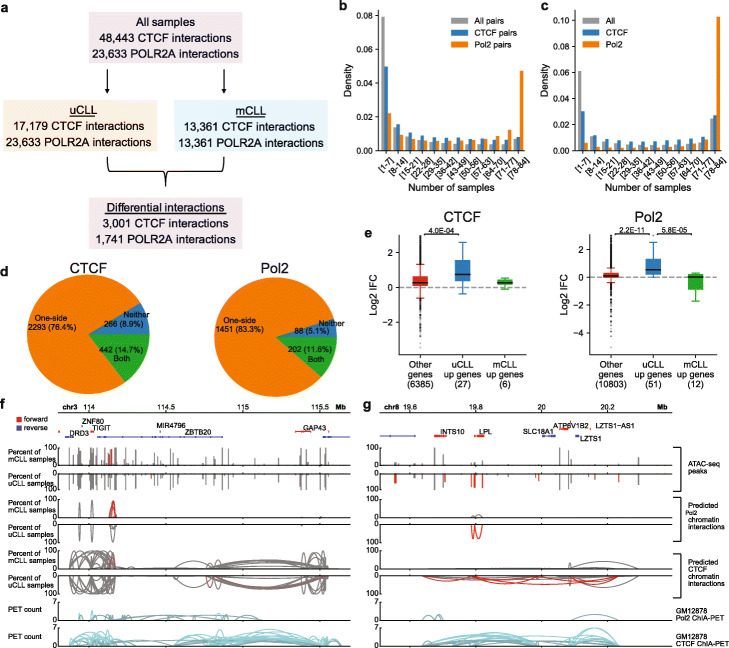
Predicted chromatin interactions in CLL samples. The “Pol2” in the figure represents “RNA Pol II”. **a** Summary of the predicted chromatin interactions in the 84 CLL samples and the differential chromatin interactions between uCLL and mCLL samples. **b** Conservation analysis of predicted chromatin interactions in the CLL samples. All pairs, all possible pairs used for prediction. **c** Uniqueness analysis of open chromatin regions that overlap with CTCF or RNA Pol II peaks from GM12878 cells in the CLL samples. All, all open chromatin regions. **d** Distribution of differential CTCF and RNA Pol II chromatin interactions based on whether both anchors (both), one anchor (one-side), or neither anchors (neither) showed the same level of differences between uCLL and mCLL samples as the associated chromatin interaction. **e** Association of differences in chromatin interactions between uCLL and mCLL samples with differentially expressed genes identified from a set of microarray samples. IFC, the fold change of the average number of chromatin interactions observed at the gene promoter in uCLL samples over that in mCLL samples. *p*-values were calculated using the Kruskal-Wallis test. **f**, **g** Examples of genes, *ZBTB20* and *LPL*, whose different connectivity are associated with differences in distal regions. The red bars and curves indicate significantly different open chromatin regions and chromatin interactions based on Fisher’s exact test

A total of 48,443 CTCF-associated open chromatin interactions and 23,633 RNA Pol II-associated open chromatin interactions were predicted based on the pooled open chromatin regions of all samples (Fig. 7a). RNA Pol II-associated chromatin interactions were better conserved across the CLL samples than CTCF-associated chromatin interactions (Fig. 7b), which could be attributed to that open chromatin regions in the CLL samples that overlapped with GM12878 RNA Pol II peaks were better conserved than those overlapping with GM12878 CTCF peaks (Fig. 7c). Using this set of ATAC-seq data in CLL samples, it was reported that regions with higher open chromatin signals in uCLL samples showed strong enrichment of binding sites of CTCF, RAD21 and SMC3 [28], which could also contribute to the high variability of CTCF chromatin interactions. Moreover, we again observed extensive patient heterogeneity of CTCF and RNA Pol II-associated predicted open chromatin interactions in these clinical samples.

**Fig. 8:**
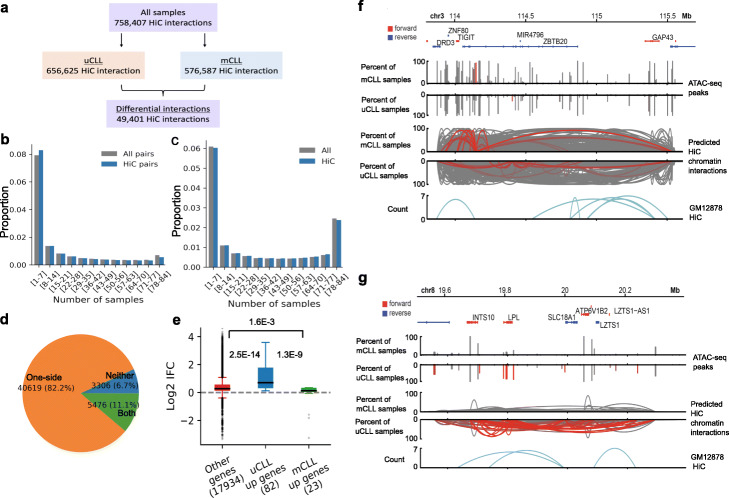
Predicted chromatin interactions in CLL samples using GM12878 Hi-C model. **a** Summary of the predicted chromatin interactions in the 84 CLL samples and the differential chromatin interactions between uCLL and mCLL samples. **b** Conservation analysis of predicted chromatin interactions in the CLL samples. All pairs, all possible pairs used for prediction; *y*-axis, the proportion of total chromatin interactions that can be found in a particular number of samples. **c** Uniqueness analysis of open chromatin regions that overlap with Hi-C peaks from GM12878 cells in the CLL samples. All, all open chromatin regions; *y*-axis, the proportion of total chromatin interactions that can be found in a particular number of samples. **d** Distribution of differential Hi-C chromatin interactions based on whether both anchors (both), one anchor (one-side), or neither anchors (neither) showed the same level of differences between uCLL and mCLL samples as the associated chromatin interaction. **e** Association of differences in chromatin interactions between uCLL and mCLL samples with differentially expressed genes identified from a set of microarray samples. IFC, the fold change of the average number of chromatin interactions observed at the gene promoter in uCLL samples over that in mCLL samples. *p*-values were calculated using the Kruskal-Wallis test. **f**, **g** Examples of genes, *ZBTB20* and *LPL*, whose different connectivity are associated with differences in distal regions. The red bars and curves indicate significantly different open chromatin regions and chromatin interactions based on Fisher’s exact test

When applying the GM12878 Hi-C model to the CLL samples, a total of 758,407 Hi-C-associated open chromatin interactions were predicted (Fig. 8a). The phenomenon observed from the CTCF model also can be observed from the Hi-C model, for example, the chromatin interactions across the CLL samples and the overlapping peaks between CLL samples and GM12878 Hi-C peaks were not well conserved as that of RNA Pol II (Fig. 8b, c). The predicted open chromatin interactions by Hi-C model were able to separate mCLL and uCLL samples (Additional file 1: Fig. S10a). Most differential chromatin interactions were associated with changes in the occurrence of one anchor (Fig. 8d). Genes that were upregulated in uCLL were associated with uCLL-specific chromatin interactions (Fig. 8e). In the set of differential chromatin interactions whose anchors did not have the same level of changes as the chromatin interactions themselves between the two subtypes, the rate of co-occurrences of the two anchors within the same sample and the levels in chromatin interactions could change (Additional file 1: Fig. S10b). Examples of predicted open chromatin interactions are shown in Fig. 8f and g and Additional file 1: Fig. S10e-h. Thus, we observed extensive patient heterogeneity of Hi-C predicted open chromatin interactions in these clinical samples.

Using the predicted open chromatin interactions, it was possible to separate mCLL and uCLL samples (Additional file 1: Fig. S11a). Variations in occurrences of open chromatin interactions between the two subtypes of CLL were associated with variations in occurrences of anchor regions. Most differential ChIA-PET chromatin interactions were associated with changes in the occurrence of one anchor (Fig. 7d). There was a small portion of differential chromatin interactions whose anchors did not have the same level of changes as the chromatin interactions themselves between the two subtypes. In this set of differential chromatin interactions, the rate of co-occurrences of the two anchors within the same sample could change, contributing to the levels of changes in predicted open chromatin interactions (Additional file 1: Fig. S11b). With the GM12878 Hi-C model, we were also able to see differences in connectivity at transcription start sites associated with differences in the occurrences of the open chromatin regions at the transcription start sites (Additional file 1: Fig. S10d).

Genes with higher expression in uCLL showed higher connectivity at the transcription start sites (Fig. 7e, Additional file 1: Fig. S11c, Fig. 6e, Additional file 1: Fig. S10c). The differences in connectivity at transcription start sites were associated with differences in the occurrences of the open chromatin regions at the transcription start sites between CLL subtypes (Additional file 1: Fig. S10d and Additional file 1: Fig. S11d), and also, differences in connectivity were sometimes associated with differences in distal interacting regions (Additional file 1: Fig. S11e, Fig. 7f). Examples of predicted open chromatin interactions are shown at important CLL prognostic markers, such as *LPL* (Fig. 7g), *ZAP70* (Additional file 1: Fig. S11f), *ZNF667* (Additional file 1: Fig. S11g), and *CD38* (Additional file 1: Fig. S11h) [45–48]. Taken together, our results indicate that different subtypes show different profiles of predicted open chromatin interactions. Different subtypes may be a source of patient heterogeneity in clinical samples.

## Discussion

In this manuscript, we described two methods of predicting chromatin interactions, first, a functional genomics approach which uses local epigenomics data to accurately predict chromatin interactions, and second, a convolutional neural network, ChINN, which can extract sequence features and be coupled to classifiers to predict chromatin interactions between open chromatin regions using DNA sequences and distance.

We showed that at resolutions limited by the experimental techniques, chromatin interactions between open chromatin regions could be predicted from 1-dimensional functional genomics data through the fact that the cross-sample model can capture the chromatin interactions. ChINN only requires the use of open chromatin data and showed good generalizability on the same type of chromatin interactions across different cell types. Thus, it has the potential to be applied to large sets of clinical samples with limited biological materials. In addition, ChINN can discover sequence features that are important for predicting chromatin interactions, including shared features such as the CTCF motif and cell-type specific features such as GATA3 binding motif in MCF-7, which is frequently mutated in breast cancer [49]. Also, we could validate ChINN-identified chromatin interactions by 4C.

In distance-controlled experiments, our prediction method using functional genomics data performed better on RNA Pol II chromatin interactions but worse on CTCF chromatin interactions compared to sequence-based ChINN. Such differences could be attributed to the lower functional genomic complexity at CTCF binding sites and functional genomic data might fail to capture the convergent CTCF binding motifs often observed at CTCF-mediated chromatin interactions.

We also noticed that the models trained using sequence features of CTCF ChIA-PET data perform better than the models trained using functional genomics data from CTCF ChIA-PET in the cross-sample prediction. We reason that the difference of the performance may be explained by the different resolution of the data. ChIP-seq data can yield ChIP-seq peaks of over several hundred bp long (and they are further generalized into count data when preparing the input features), while the CTCF motif is only less than 20 bp (CTCF motif MA0139.1 from JASPAR database). In addition, the ChIP-seq peaks cannot tell the orientation of the CTCF binding, while sequence can tell the direction of CTCF motif. As CTCF orientation is found to be important in chromatin interactions [41], the sequence feature can give more information of the binding site as well as the binding orientation. Therefore, the information of CTCF in these two cross-sample prediction results is different.

On the other hand, RNA Pol II binding sites do not have such distinctive DNA motifs, making it harder to predict RNA Pol II binding sites [13, 14] and consequently harder to predict RNA Pol II-associated chromatin interactions from DNA sequences. However, RNA Pol II binding sites are usually occupied by many other transcription factors, making it easier to predict RNA Pol II-associated chromatin interactions using functional genomic data.

The application of ChINN models with gradient boosted tree classifiers to a set of CLL ATAC-seq samples showed that several of the predicted open chromatin interactions could be validated by Hi-C. However, we note that the auPRC scores of ChINN reported, particularly the GM12878 and K562 models applied to explore chromatin interactions in patient samples, was around 0.26–0.6, which is consistent with cross-sample testing of other epigenomics machine learning prediction methods such as DeepHistone [50]. However, these auPRC scores are not very high, which could be due to several reasons.

First, auPRC is a performance metric that is usually not very high, especially when the number of negative samples hugely overwhelm the positive samples. Because the number of chromatin interactions in the entire genome, relative to the number of genomic regions with no reported chromatin interactions by Hi-C, is not very high; therefore, the number of negative samples in our data hugely overwhelm the positive samples. As such, it is expected that the auPRC score will not be very high when applying GM12878 and K562 Hi-C models to other cell lines or patient samples.

Second, the ChINN method only takes as input the sequences of DNA at open chromatin regions of the genome for prediction. If more types of data are input into the model, the performance of the model is likely to improve, but at the cost of requiring more datatypes which are expensive and labor-intensive to acquire.

Third, while there were also chromatin interactions that were predicted but not validated by Hi-C, our results showing that 4C could validate predicted chromatin interactions in MCF-7 cells that were not identified by Hi-C suggest that these so-called “false positives” might potentially be real chromatin interactions that were simply not captured by Hi-C due to limited sequencing depth of Hi-C libraries.

In future work, further development of Hi-C and other chromatin interaction sequencing methods to comprehensively capture chromatin interactions will allow for a better comparison with chromatin interaction predictions. Additionally, further development and refinement of ChINN to improve the accuracy of chromatin interaction prediction is warranted.

Application of ChINN models in CLL revealed that although there were open chromatin interactions that were ubiquitous in all samples, there were a large number of patient-specific open chromatin interactions and also chromatin interactions that were found in fewer than half the samples. We note that chromatin interactions predicted using cross-sample models are likely to show less cell-type specificity, and the fact that sample heterogeneity can be seen in these predicted open chromatin interactions in spite of the lower likelihood of cell-type specificity due to the nature of the chromatin interaction prediction, suggests that chromatin interaction heterogeneity is widespread throughout the genome. Moreover, the observation of predicted open chromatin interaction heterogeneity agrees with our observations that there exist both ubiquitous chromatin interactions and patient-specific chromatin interactions in the 6 Hi-C libraries from the 6 CLL patient samples that we examined. While we previously observed patient-specific chromatin interactions at particular loci [51], here, we show that this phenomenon is widespread. To the best of our knowledge, this observation of widespread nature of patient-specific chromatin interactions is novel and has not been previously reported in the 3D genome organization field.

One potential reason for these different chromatin interactions could be due to different patient subtypes. Importantly, we found systematic differences in chromatin interactions involving important CLL prognostic genes, such as *LPL* and *CD38,* between the IGHV-mutated and IGHV-unmutated subtypes. These results suggest that differences in chromatin interaction landscapes between CLL subtypes could have important functional implications in CLL biology. Moreover, differences in chromatin interaction presence or absence may lead to different expression of oncogenes in cancers.

Our observation of widespread patient heterogeneity in patient cancer samples highlights the need for precision medicine and the need to understand chromatin interactions in individual patient samples. Machine learning offers one way for us to predict chromatin interactions in a cost-effective manner. The ChINN method may be useful in the future in understanding chromatin interactions in large cohorts of clinical samples and identifying chromatin interaction-based biomarkers that can be used to distinguish between different subtypes of cancer which may help in the development of precise therapies for the different subtypes of cancer.

## Conclusion

A functional genomics approach is able to predict chromatin interactions. The ChINN framework is able to predict chromatin interactions from open chromatin regions in the human genome, using DNA sequences and distances as features. This framework can be applied in other cell lines or clinical samples given the knowledge of open chromatin regions, making it a useful tool to interrogate chromatin interactions when large-scale functional genomics acquisition is not applicable due to limited biological materials.

## Methods

We performed machine learning, Hi-C interaction analysis, ATAC-seq, RNA-seq, and gene expression analyses as described in the following sections. The quality information of generated Hi-C, ATAC-seq, and RNA-seq libraries can be found in Additional file 1: Table S7, Additional file 1: Table S8, and Additional file 1: Fig. S12.

### Machine learning of ChIA-PET data

The development of the sequence models was divided into three stages. In the first stage, the distance-matched datasets were used to train the models consist of convolutional neural network (feature extractor) with fully connected layers as the classier, as shown in Fig. 2a. The first stage deep learning method works as a feature extractor to convert the raw sequence feature to numerical representation that can be used as input of the machine learning models. Stages 2 and 3 aim to train the different machine learning models to make the prediction. In the second and third stage, the feature extractors trained in the first stage were frozen and gradient tree boosting classifiers were used as classifiers. In the second stage, the gradient tree boosting classifiers were trained using the extended datasets. In the third stage, the gradient tree boosting classifiers were trained using all potential pairs of anchors generated from open chromatin data and annotated by existing ChIA-PET data. Thus, the final result was a program that took in a list of open chromatin regions and produced predictions of chromatin interactions between the open chromatin regions.

The feature extractors took DNA sequences of both anchors of a potential interacting pair as input. The classier then took the features generated by the feature extractor and optionally the distance between anchors as input and produced a probability score of interaction. This final model was defined as the “from open chromatin” model. More details can be found from Additional file 1: Supplementary Methods [52–69].

### Machine learning of Hi-C data from cell lines

We collected the Hi-C interactions from 8 cell lines, including GM12878, HeLaS3, HMEC, HUVEC, IMR90, K562, KBM7, and NHEK. The construction of machine learning model using Hi-C data from cell lines follows the same procedures as described in that of ChIA-PET data, where the positive data is annotated according to the Hi-C interactions.

### Machine learning of Hi-C data from clinical samples

We collected the Hi-C interactions from 6 CLL clinical samples, including CLL 102, CLL 312, CLL 324, CLL 344, CLL 401, and CLL 484. The construction of machine learning model using Hi-C data from cell lines follows the same procedures as described in that of ChIA-PET data, where the positive data is annotated according to the Hi-C interactions. The CLL 401 model was used in the across-sample prediction.

### Preparation of clinical samples

Chronic lymphocytic leukemia patient samples (either peripheral blood or bone marrow isolates) were obtained from the Leukemia Cell Bank at the National University Health System (NUHS) with patient consent, under Institute Review Board number H-20-022E. The CLL samples were either bone marrow aspirates (312,324,344,484 and 102) or peripheral blood (401). The samples were immediately frozen after collection and stored in liquid nitrogen until further use.

The samples were taken out of the liquid nitrogen and thawed by dipping in a beaker containing water at 37 °C. Once the sample was thawed completely, the cells were immediately transferred to the 15 ml falcon and resuspended in 10 ml PBS containing 2% fetal bovine serum (FBS) and 2 mM EDTA. The cells were pelleted at 300×*g* for 5 min at room temperature and resuspended in 5 ml PBS containing 2% FBS and 2 mM EDTA. The cells were counted and checked for viability using Trypan Blue.

RNA and genomic DNA were isolated from the CLL patient samples using AllPrep DNA/RNA/miRNA universal kit (Qiagen) according to the manufacturer’s instructions. Briefly, cells lysate were homogenized by a 21-G needle and syringe together with lysis buffer and 1 M DTT. After that, the homogenized lysate were transferred into AllPrep DNA mini spin column for genomic DNA extraction. The genomic DNA were then eluted by water and proceeded for the IGHV mutation test. The flow through after the AllPrep DNA mini spin column was then proceeded into RNease Mini spin column with on-column digestion for RNA extraction. The RNA were eluted in water and further sent for RNA-seq.

IGHV mutation test was performed following the method in Agathangelidis et al. [70]. Briefly, IGHV-IGHD-IGHJ gene rearrangements were amplified by 5′ IGHV leader primers and 3′ IGHJ primers (primer sequences are provided in Additional file 1: Table S9) using genomic DNA (gDNA) from CLL patient samples. The PCR amplification was performed by PCR core kit (Qiagen). Final PCR products were imaged by agarose gel electrophoresis and purified by PCR purification kit (QIAGEN). Purified PCR products were confirmed through Sanger sequencing by 3′ IGHJ primers. The Sanger sequencing results were analyzed by IMGT/V-QUEST tools [71] to get the IGHV identity scores. If the identity score was larger than 98%, the CLL sample was considered an unmutated sample while if the score was lower than 98%, the CLL sample was considered a mutated sample.

### In situ Hi-C

Hi-C libraries were prepared using the Arima Genomics kit (Arima Genomics, San Diego, CA) in conjunction with the Swift Biosciences Accel-NGS 2S Plus DNA Library Kit (Cat # 21024) and Swift Biosciences Indexing Kit (Cat # 26148) following the manufacturer’s recommendations. In brief, 1X 10^6^ cells were fixed with formaldehyde in the nucleus. Fixed cells were permeabilized using a lysis buffer and then digested with a restriction enzyme cocktail supplied in the Arima Hi-C kit. The resulting overhangs were filled in with biotinylated nucleotides followed by ligation. After ligation, crosslinks were reversed, and the DNA was purified from protein. Purified DNA was treated to remove biotin that was not internal to ligated fragments. Hi-C material was then sonicated using a Covaris Focused-Ultrasonicator M220 instrument to achieve 300–500 bp fragment sizes. The sonicated DNA was double-size selected using Ampure XP beads, and the sequencing libraries were generated using low input Swift Biosciences Accel-NGS 2S Plus DNA Library Kit (Cat # 21024) and Swift Biosciences Indexing Kit (Cat # 26148). The Hi-C libraries were loaded on an Illumina flow cell for paired-end 150-nucleotide read length sequencing on the Illumina HiSeq 4000 following the manufacturer’s protocols.

### Cell culture

MCF-7, a breast cancer cell line, was cultured in DMEM/F12 (Gibco) supplemented with 10% FBS and 1% penicillin-streptomycin and maintained at 37 °C, 5% CO_2_ humidified incubator. Before 4C-seq assays, MCF-7 cells were grown in hormone-free media: they were washed with PBS twice to remove any residual FBS or growth factors and incubated in phenol red-free medium (Invitrogen/Gibco) supplemented with 10% charcoal-dextran stripped FBS (Hyclone) and 1% pencillin-streptomycin for a minimum of 72 h. Hormone-depleted MCF-7 cells were then treated with estrogen (Sigma) to a final concentration of 100 nM for 45 min before 4C-seq assay. The control cells were treated with an equal volume and concentration of vehicle, ethanol (Sigma), for 45 min.

### Circular chromosome conformation capture (4C)

4C-seq assays were performed according to Splinter et al [72] with slight modifications. Briefly, 4 × 10^7^ cells were crosslinked with 1% formaldehyde. The nuclei pellets were isolated by cell lysis with cold lysis buffer (10 mM Tris-HCl, 10 mM NaCl, 5 mM EDTA, 0.5% NP 40) supplemented with protease inhibitors (Roche). First step digestion was performed overnight at 37 °C with HindIII enzyme (NEB). Digestion efficiency was measured by RT-qPCR with HindIII site-specific primers. After confirmation of good digestion efficiency, DNA was ligated overnight at 16 °C by T4 DNA ligase (Thermo Scientific) and de-crosslinked. Following de-crosslinking, DNA was extracted by phenol-chloroform and this is the 3C library. The DNA was then processed for second digestion with DpnII enzyme (NEB) overnight at 37 °C. After final ligation, 4C template DNA was obtained, and the concentration was determined using Qubit assays (Thermo Scientific). The 4C template DNA was then amplified using specific primers with Illumina Nextera adapters and sent for sequencing on the MiSeq system. All the 4C genome coordinates are listed in Additional file 1: Table S9.

### RNA-seq

Total RNA was extracted from the CLL samples using the All Prep DNA/RNA kit (Qiagen). The RNA was quantified using the Qubit BR RNA Assay kit. RNA-seq libraries (strand specific and ribo zero) were constructed using Illumina Total RNA Prep kit (Illumina, San Diego, CA, USA) and sequenced 150 bases paired-end on the Illumina HiSeq 4000 following the manufacturer’s instruction.

### ATAC-seq

ATAC-seq library was prepared as described previously [73]. Briefly, 50,000 cells were lysed for nuclei isolation using ATAC-Resuspention Buffer containing 0.1% NP40, 0.1% Tween-20, and 0.01% Digitonin. Transposition reaction was performed for 30 min at 37 °C using Nextera DNA library preparation kit (NEB). Transposed fragments were amplified by eight PCR cycles for library preparation. Primer dimers and long DNA fragments were removed by AMPure XP beads purification step. DNA concentration was measured by Qubit fluorometric assay and library quality was determined by Bioanalyzer. The library was sequenced in Nextseq 500 76 bp paired-end configuration using Illumina platform.

## Supplementary Information

**Additional file 1.** Supplementary text, methods, figures, and tables.

**Additional file 2.** Review history.

## Data Availability

All relevant data supporting the key findings of this study are available within the article and its Additional files. The datasets of RNA-Seq, ATAC-Seq and Hi-C, which are generated during the current study are available in GEO under accession number GSE163896 [74]. The 4C data has been deposited with GEO accession number GSE135052 [75] and is publicly available. The source codes of ChINN are freely available under Apache License 2.0 at https://github.com/mjflab/chinn [76]. The code used for this paper has also been deposited at Zenodo with DOI 10.5281/zenodo.5139249 [77].
