# Supplementary material for: Chromatin interaction neural network (ChINN): a machine learning-based method for predicting chromatin interactions from DNA sequences
Source: Genome Biol. 2021 Aug 16;22:226. doi: 10.1186/s13059-021-02453-5 (PMC8365954; doi:10.1186/s13059-021-02453-5)
Supplement: Supplementary file 1 — Additional file 1. Supplementary text, methods, figures, and tables. [file 13059_2021_2453_MOESM1_ESM.pdf]

Chromatin Interaction Neural Network (ChINN): A machine learning-based method for predicting chromatin interactions from DNA sequences - Supplementary Materials

Fan Cao[1*], caofan@outlook.com
Yu Zhang[2*], yu007@e.ntu.edu.sg
Yichao Cai[1*], csicaiy@nus.edu.sg
Sambhavi Animesh[1], csisam@nus.edu.sg
Ying Zhang[1], csiyz@nus.edu.sg
Semih Can Akincilar[3], semiha@imcb.a-star.edu.sg
Yan Ping Loh[1], lohyanping@gmail.com
Xinya Li[4], LIXI0068@e.ntu.edu.sg
Wee Joo Chng[1,5,6], csicwj@nus.edu.sg
Vinay Tergaonkar[3], vinayt@imcb.a-star.edu.sg
Chee Keong Kwoh[2], asckkwoh@ntu.edu.sg
Melissa J. Fullwood[1,3,4,^], mfullwood@ntu.edu.sg

[1]Cancer Science Institute of Singapore, National University of Singapore, 14 Medical Dr, Singapore 117599.
[2]School of Computer Science and Engineering, Nanyang Technological University, Block N4, 50 Nanyang Avenue, Singapore 639798.
[3]Institute of Molecular and Cell Biology, Agency for Science, Technology and Research (A*STAR), 61 Biopolis Drive, Proteos, Singapore 138673.
[4]School of Biological Sciences, Nanyang Technological University, 60 Nanyang Drive, Singapore 637551.
[5]Department of Medicine, Yong Loo Lin School of Medicine, National University of Singapore. 1E Kent Ridge Road, Singapore 119228.
[6]Department of Haematology-Oncology, National University Cancer Institute, National university Health System, NUH Zone B, Medical Centre, Singapore 119074.
*Cofirst authors
^Corresponding author

Contents

1 Text S1: An explanation of the performance of distance feature 4

2 Supplementary Methods 4

2.1 Generation of positive and negative chromatin interactions 4
2.2 Overview of the functional genomic models 6
2.3 Generating features for functional genomic models 7
2.4 Training and evaluation of functional genomic models 7
2.5 Overview of the sequence models 8
2.6 Preparing input for sequence models 8
2.7 The sequence model design considerations 8
2.8 Mathematical formation of the components used 9
2.9 Training and evaluation of the sequence models on distance-controlled datasets 10
2.10 Examining sequence features 11
2.11 Training gradient boosted trees using extended datasets 11
2.12 CTCF-only models 12
2.13 Training gradient boosted trees using pairs of open chromatin regions in GM12878 12
2.14 4C-seq library generation 13
2.15 4C-seq data analysis 14
2.16 RT-qPCR experiments 14
2.17 Analysis of chronic lymphocytic leukemia samples 14

3 Supplementary Figure Legends 17

3.1 Fig. S1: Characteristics of positive and negative samples in distance-matched datasets 17
3.2 Fig. S2: Convolutional layer 1 kernels of the five models that resemble known transcription factor binding motifs 17
3.3 Fig. S3: Sequence models on extended datasets 17
3.4 Fig. S4: Hi-C sequence models on extended datasets 17
3.5 Fig. S5: Selection of parameters for from-dnase models and validations by 4C-seq in MCF-7 cells 18
3.6 Fig. S6a-d: Selection of parameters for Hi-C models and validations by 4C-seq in MCF-7 cells 18
3.7 Fig. S7: A diagram of user demands among different chromatin interactions prediction using sequences 18
3.8 Fig. S8: The F-score with different thresholds for K562 and GM12878 Hi-C model on new CLL samples 19

3.9 Fig. S9: Performances of the sequence-based and DNase models in new CLL samples. 19
3.10 Fig. S10: Analyses of predicted chromatin interactions in CLL samples using GM12878 Hi-C model 20
3.11 Fig. S11: Analyses of predicted chromatin interactions in CLL samples 20
3.12 Fig. S12: Per base quality of CLL RNA-seq libraries 21

4 Supplementary Table Legends **22**
4.1 Table S1: Description and comparison among Akita, DeepC, DeepTACT, and SEPT. 22
4.2 Table S2: Negative-to-positive ratios of samples on each chromosome of distance-matched datasets. 22
4.3 Table S3: List of ChIP-seq peaks downloaded from ENCODE. . . . 22
4.4 Table S4: Convolutional layer 1 kernels of the eight Hi-C models from cell lines that resemble known transcription factor binding motifs. 22
4.5 Table S5: The number of cell lines for each motif that was detected in eight Hi-C models 22
4.6 Table S6: Clinical characteristics of the CLL samples 22
4.7 Table S7: Hi-C libraries summary of CLL samples 22
4.8 Table S8: ATAC-seq libraries summary of CLL samples 22
4.9 Table S9: Primer information 22
4.10 Table S10: F1-score calculated with different cut-off values from pairs of open chromatin regions in GM12878 cells. 22

5 Supplementary Figures **23**

6 Supplementary Tables **40**

1 Text S1: An explanation of the performance of distance feature

The reason why genomic features along with distance feature can achieve significantly better performance than that using only genomic features can be explained from the Gradient boosted trees (GB) model that we used. The core of GB is decision tree. In a decision tree, the features would be selected as nodes to split the dataset. When using distance feature only, it is certainly impossible to split the dataset to positive or negative as there is only one split and the data are similar. But when using distance feature with other features together, the original datasets may already been split to small subsets by other features before using distance feature. In those small subsets split according to certain rules, distance feature may play some roles to split them to even smaller subsets. Besides, by adding one more feature, the pruning of the tree could also change, which may lead to different prediction results compared to without adding the feature.

2 Supplementary Methods

2.1 Generation of positive and negative chromatin interactions

The GM12878 CTCF, GM12878 RNA Pol II, and Hela-S3 CTCF ChIA-PET datasets were downloaded from supplementary materials of Tang et al[1]. Alignment files of K562 RNA Pol II and MCF-7 RNA Pol II ChIA-PET datasets were obtained from Li et al[2] and processed as described in Li et al to call chromatin interactions.

Chromatin interactions that overlap with ENCODE blacklisted regions[3] at either anchor were discarded. Anchors of remaining chromatin interactions in each dataset were merged by allowing 500 bp gap[1]. The chromatin interactions in each dataset were clustered again using the merged anchors. Clustered chromatin interactions were filtered against DNase-seq data of the same cell line by requiring both anchors to overlap with DNase I hypersensitivity sites. The remaining clustered chromatin interactions were used as positive samples for each dataset.

For each dataset, corresponding negative samples were generated from four sources:

Source 1. Pairs of clustered chromatin interaction anchors that were not directly or indirectly connected were used as negative samples. We build networks of merged anchors where the merged anchors were nodes and the chromatin interactions were edges. Two nodes were considered as directly connected if

there existed an edge between them. Two nodes were considered as indirectly connected if there existed a path of nodes and edges between the two nodes in the network.

Source 2. Random pairs of peaks from ChIP-seq data of the transcription factor from which the ChIA-PET library was built. The ChIP-Seq data and ChIA-PET data are from the same cell line. Peak pairs from CTCF ChIP-Seq data were used for CTCF ChIA-PET datasets and peak pairs from RNA Pol II ChIP-Seq data were used for RNA Pol II ChIA-PET datasets. The peaks pairs were generated as follows:

(a) Transcription factor peaks that overlap with ENCODE blacklisted regions were filtered.

(b) From the remaining peaks, peaks that overlap with DNase I hypersensitivity regions were extracted.

(c) For each remaining peak, sample a size from a normal distribution. If the size of the peak is smaller than the sampled size, extend the peak to match the sampled size. The extended peaks were then merged. The mean and variance of the normal distribution were adjusted empirically to make the distribution of the sizes of merged peaks match the distribution of anchor sizes of the positive samples.

(d) All pairs of merged peaks with distance ranging from 5 kb to 2 Mb and were not directly or indirectly connected were kept.

Source 3. Random pairs of peaks from DNase-I data of the cell line from which the ChIA-PET library was built. The peak pairs were generated as follows:

(a) DNase-I peaks that overlap with ENCODE blacklisted regions were filtered.

(b) For each remaining peak, sample a size from a normal distribution. If the size of the peak is smaller than the sampled size, extend the peak to match the sampled size. The extended peaks are then merged. The mean and variance of the normal distribution were adjusted empirically to make the distribution of the sizes of merged peaks match the distribution of anchor sizes of the positive samples.

(c) All pairs of merged peaks with distance ranging from 5 kb to 2 Mb and were not directly or indirectly connected were kept. In addition, this set of negative samples were filtered against the negative samples generated using transcription factor peaks to reduce redundancy among negative samples.

Source 4. Pairs of clustered chromatin interaction anchors that were indirectly connected.

We generated two types of negative datasets: distance-matched negative datasets and extended negative datasets.

For each positive dataset, a distance-matched negative dataset was generated by sampling negative samples from the first three sources such that the positive-to-negative ratio was about 1:5 for every chromosome and the distance distributions were matched (Table S2). We split the 5kb-2Mb distance range into 50 bins on the logarithm scale and counted the number of positive samples in each bin for each chromosome. Negative samples were then sampled such that there were about 5 negative samples for 1 positive sample in each bin of every chromosome. If the number of negative samples was less than 5 times of positive samples in a bin of a chromosome, more negative samples might be sampled from the same bin of another chromosome to make up the difference. Different types of negative samples were prioritized differently. Anchor pairs (Source 1) and transcription factor peak pairs (Source 2) were sampled first, and if there were not enough negative samples in these two sources, additional negative samples were sampled from DNase-I peak pairs (Source 3).

For each positive dataset, an extended negative dataset was generated by including pairs from Source 4, the corresponding distance-matched negative dataset, and the remaining negative samples from Source 1 and Source 2. This dataset is called the "extended dataset" in the main text.

Samples on chromosomes 5 and 14 were used for validation. Samples on chromosomes 4, 7, 8, and 11 were used for test. The rest samples were used for training. The validation sets were used to guide the selection of the trained models. The test sets were used to obtained the final performance metrics and were strictly not used in training or selection of models.

Hi-C datasets were generated similarly as ChIA-PET. The cell line Hi-C interactions of GM12878, HeLa, HMEC, HUVEC, IMR90, K562, KBM7 and NHEK were downloaded from Rao et al[4].

We note that here, "bin" does not refer to a segment of a chromosome; it is a segment of the distance distribution histogram.

2.2 Overview of the functional genomic models

The functional genomic models were based on gradient tree boosting classifiers with features generated from functional genomic data such as ChIP-seq data and DNase-seq data. We and others have shown that many of the "state-of-art" methods based on functional genomic data had highly exaggerated performances and thus whether chromatin interaction could be accurately predicted from functional genomic data

became unclear. This made our premise, chromatin interactions could be predicted from functional genomic data and functional genomic data could be predicted from DNA sequences, for developing predictive models based on DNA sequences broken. The purpose of developing the functional genomic models here was to confirm that chromatin interactions could be predicted from functional genomic data. For this purpose, we only trained and evaluated the models on distance-matched datasets.

2.3 Generating features for functional genomic models

ChIP-seq peaks of histone marks and transcription factors were downloaded from ENCODE[3]. Histone marks and transcription factors that were common to K562, GM12878, and HelaS3 cells and DNase-seq peaks were used (Table S1). For each sample, the feature values were calculated for both anchor independently. For an anchor a, let F_a be its feature vector. Then the anchor's feature value of a transcription factor/histone modification/DNase f is calculated as

$$F_{af} = \sum_i o_i^{af} v_i^f / s^a,$$

where s^a is the size of the anchor, o_i^{af} is the overlap size between the anchor a and peak i of factor f, and v_i^f is the average signal value of peak i of factor f. In short, a feature value of an anchor is the weighted sum of the signals of overlapping peaks of a factor, where the weights are the proportions of anchors in the overlaps.

The distance feature was calculated as the distance between the centers of two anchors.

We also evaluated models based on peak counts instead of signal values. When peak counts were used as features instead of signal values, we counted the peaks of a factor that overlap with an anchor and used the counts as feature values. In this case, a peak was only considered as overlapping with the anchor if the overlap size was at least 100 bp or at least 50% of the size of either the peak or the anchor.

2.4 Training and evaluation of functional genomic models

We built functional genomic models for GM12878 CTCF, GM12878 RNA Pol II, HelaS3 CTCF, and K562 RNA Pol II datasets individually. The functional genomic models were constructed using gradient boosted trees from the XGBoost library[5]. Parameters used were: $max\_depth = 6$, $eta = 0.1$, $eval\_metric = logloss$. The models were trained for 1000 iterations with early stopping when the performances on validation sets were not improved for 40 consecutive iterations. The iteration of the models that produced best performance on validation sets were used as the final trained model for each dataset. The performance metrics were then obtained by applying the final trained models on the test sets.

2.5 Overview of the sequence models

The development of the sequence models was divided into three stages. In the first stage, the distance-matched datasets were used to train the models consist of convolutional neural network (feature extractor) with fully-connected layers as the classifier, as shown in Figure 2a. In the second and third stage, the feature extractors trained in the first stage were frozen and gradient tree boosting classifiers were used as classifiers. In the second stage, the gradient tree boosting classifiers were trained using the extended datasets. In the third stage, the gradient tree boosting classifiers were trained using all potential pairs of anchors generated from open chromatin data and annotated by existing ChIA-PET data. Thus, the final result was a program that took in a list of open chromatin regions and produced predictions of chromatin interactions between the open chromatin regions.

The feature extractors took DNA sequences of both anchors of a potential interacting pair as input. The classifier then took the features generated by the feature extractor and optionally the distance between anchors as input and produced a probability score of interaction.

2.6 Preparing input for sequence models

We used the human genome assembly *hg19* as our reference. The sequences of all anchors were extracted and converted to $4 \times L$ one-hot coded matrices, where L was the length of the sequence. The four rows of the matrices represents the occurrences of nucleotides A, G, C, and T, respectively. Thus, nucleotide A was represented as $[1, 0, 0, 0]^T$, nucleotide G was represented as $[0, 1, 0, 0]^T$, nucleotide C was represented as $[0, 0, 1, 0]^T$, and nucleotide T was represented as $[0, 0, 0, 1]^T$. If N was encountered in the sequence, it was represented as $[0.25, 0.25, 0.25, 0.25]^T$. For each sequence, we split it into 1000 bp subregions with 500 bp overlap between consecutive subregions.

The distance feature was calculated as the distance between the centers of two anchors.

2.7 The sequence model design considerations

A typical model based on convolutional neural network consists of two parts, the feature extracting convolutional layers (feature extractor) and the fully-connected layers (classifier) that aggregate the extract features and produce predictions or regressions. A convolutional layer is a stack of kernels, where each kernel is cross-correlated (convoluted) with the input independently, producing measures of the local similarities between the input and kernel. Non-linearities such as rectifier linear unit (ReLU) are applied on the outputs of convolutional layers to break the

linearity. Convolutional layers are usually followed by max pooling layers to both reduce the spatial dimension of the output and allow small translations of the features on spatial dimensions. Max pooling layers take local maxima of convolutional outputs of each kernel independently. The output of the last convolutional layer is usually flattened and fed into the fully-connected layers to make predictions or regressions.

In this study, we used a weight-sum layer[6] after the last convolutional layer to obtain an aggregated score of each kernel in the last convolutional layer. The weighted-sum layer learns a weight distribution for each channel in the input independently and calculates the weighted sum of each channel along the spatial dimensions. Intuitively, the weighted-sum layer learns to weight the detected features at different spatial regions. For example, a weight-sum layer could have high weights for features detected near the center of the input sequence while penalizes features detected in surrounding regions by giving them negative weights. The weight-sum layer is followed by a non-linear activation function such as *tanh* or *sigmoid*. We used *tanh* for CTCF datasets and *sigmoid* for RNA Pol II datasets.

The sizes of chromatin interactions anchors vary substantially, ranging from kilo basepairs to tens of kilo basepairs. Thus, the network needs to accommodate the different anchor sizes. For each anchor, we split it into 1000bp subregions with 500bp overlap between consecutive subregions. The sequences of the subregions were fed into the convolutional layers independently. Following the non-linear function after weight-sum layer, maxima of each kernel across all subregions of an anchor were taken as the feature value for the anchor. Consequently, each anchor, regardless of its size, is represented by the same number of features.

2.8 Mathematical formation of the components used

An anchor can be represented by a matrix X of shape $N \times L \times C$, where N is the number of subregions, L is the length of the subregions and C is the number of channels (or nucleotides in this application). For each kernel k in a convolutional layer, let S be the width of the kernel and W^k of size $S \times C$ be the weight matrix of the kernel, b^k be the bias, then

$$conv(X)_{n,m,k} = leakyReLU(\sum_{i=1}^{S}\sum_{j=1}^{C} X_{n,m+i,j}W^k_{i,j} + b^k);$$

$$leakyReLU(x) = \begin{cases} x & \text{if } x \geq 0 \\ \alpha x & \text{otherwise} \end{cases},$$

where α is a small constant and is set to be 1/5.5[7].

For a pooling layer, let S be the size of the pool and T be the stride, then for input X of shape $N \times L \times C$,

$$pool(X)_{n,m,k} = \max_{i=1}^{S} X_{n,m*T+i,k}.$$

For input X of $N \times L \times C$, the output of *weightsum* is of shape $N \times C$. For a weight-sum layer, let W^{ws} be the weight matrix of shape $L \times C$, then

$$weightsum(X)_{n,k} = \sum_{i=1}^{L} X_{n,i,k} W^{ws}_{i,k}.$$

For RNA Pol II datasets, each value in the output of weight-sum layer is transformed by

$$sigmoid(x) = \frac{e^x}{1 + e^x}.$$

For CTCF datasets, each value in the output of weight-sum layer is transformed by

$$tanh(x) = \frac{e^x - e^{-x}}{e^x + e^{-x}}$$

The features values of the anchor is then generated by taking the maximum of each kernel k across all subregions by

$$maxfeature(X)_k = \max_{n=1}^{N} X_{n,k}.$$

2.9 Training and evaluation of the sequence models on distance-controlled datasets

For each dataset, a model was trained using samples from its training set. Two rounds of training were performed for each model and each round was run for 50 epochs and early stopped if the loss on validation data was not improved in consecutive 10 epochs. The first round of training used larger learning rates and weighted positive samples by the negative-to-positive ratio in the training set, which is around 5. The second round of training used smaller learning rates. For CTCF models, the weights for positive samples were removed in the second round of training. The performances of the models on validation datasets were calculated after each epoch and the model at the epoch that produced the best performance were selected. The final performance metrics were then calculated using the test sets.

2.10 Examining sequence features

The sequence patterns that kernels on the first and third convolutional layers detect were examined. For each kernel on first convolutional layer, as in DeepBind[8], we fed all test samples to the network and extracted a subsequence of each sample that pass the activation threshold of 0. If an anchor has multiple subsequences passing the activation threshold, the subsequence that has the highest activation is retained. Sequences that have no subsequences passing the activation threshold were discarded. The obtained subsequences for each kernel were then aligned to obtain a position-weight matrix for the kernel. The position-weight matrices of the kernels were compared against the transcription factor motif databases (HOCOMOCO version 11[9] and JASPAR core 2014 vertebrates collection[10]) using TomTom[11] with E-value threshold of 0.05.

For each kernel on the third convolutional layer, we generated an input matrix that maximize the output from the kernel. An input matrix was initialized randomly and fed into the network. The activation from the kernel under examination was obtained and the negation of its output was used as the loss to calculate the gradients on the input. The input matrix was then updated with stochastic gradient descent. The values of each column in the input matrix, which represents a base pair, were first clamped to the range of $[0, 1]$ and then normalized so that the sum of the values was 1. For each kernel, 20,000 iterations were performed. The resulting input matrices were then used as position-weight matrices. As these position-weight matrices are 163 bp and TomTom takes long time to compare them to transcription factor motif databases, we split them into 30 bp smaller matrices with 20 bp stride. These smaller matrices were compared to transcription factor motif databases (HOCOMOCO version 11[9] and JASPAR core 2014 vertebrates collection[10]) using TomTom[11] with E-value threshold of 0.01.

2.11 Training gradient boosted trees using extended datasets

The samples in the extended datasets were converted to matrices as described above. The convolutional layers trained using the distance-matched datasets were frozen and were used to produce feature vectors for the samples in the extended datasets. The generated feature vectors were used to train gradient tree boosting classifiers together with the distances between anchors. The splitting of samples into training, validation, and test sets were the same as described above.

We used gradient boosted trees from XGBoost library[5]. The *max_depth* setting was set to be 10, and trained for 1000 iterations and early stopped of if the loss on validation dataset is not improved in 40 consecutive iterations. The reported performance metrics were based on the test sets.

The classifiers on the five datasets achieved within-sample auPRCs ranging

from 0.4 to 0.6 (Supplementary Figure 3a). If distance was not used, the performances of the classifiers dropped significantly (Supplementary Figure 3b). However, using distance alone could not predict chromatin interactions (Supplementary Figure 3c). These results suggested that the interplay between distance and sequence features were important in predicting chromatin interactions in general. The across-sample performances of the models on extended datasets were comparable to the corresponding within-sample performances, demonstrating the generalizability of the predictive models to new cell types using only sequence features and distance (Supplementary Figure 3d).

2.12 CTCF-only models

We obtained all occurrences of CTCF motif in the genome using FIMO[12]. For each strand of each anchor, sum of matching scores of strand-specific occurrences of the CTCF motif within the anchor was calculated. The values were then normalized by the size of the anchors. Thus, for each chromatin interaction, a feature vector of four values were generated, each representing a strand of an anchor. Using these features together with distance, we trained another set of gradient boosted trees on the extended CTCF datasets.

2.13 Training gradient boosted trees using pairs of open chromatin regions in GM12878

Two parameters, merging distance and extension size, were considered in determining how to generate anchors from DNase I hypersensitivity sites. As shown in Figure 4a, merging distance determines the gap allowed between neighboring peaks to merge them and extension size determines how much to extend on both ends of the merged peaks. To decide the two parameters, we used different combinations of the two parameters to process the DNase I peaks to generate anchors. Anchor pairs were then generated from the list of anchors such that the distance between centers of the two anchors were in the range of 5 kb to 2 Mb. Anchor pairs overlapping with ChIA-PET chromatin interactions were labelled as positive and the rest were labelled as negative. We then evaluated the performances of the models trained on extended datasets achieved on these anchor pairs for all combinations of the two parameters. The combination of the two parameters that achieved best area under ROC curve was selected. The reason to use auROC is because merging using different merging distances could lead to different number of anchor pairs generated and potentially different positive-to-negative ratios. Positive-to-negative ratios could affect auPRC measures and F1 measures as their baselines are sensitive to positive-to-negative ratio.

The DNase-I hypersensitivity sites were processed using the selected merging distance and extension size to produce anchors. Pairs of anchors were generated and labelled as described above. The anchor pairs were passed through the feature extractor to extract sequence features and generate feature vectors of sequence features. The sequence feature vectors and distance between anchors were then used to train gradient boosted tree classifiers. The parameters were then same as used for the extended datasets. The splitting of samples into training, validation and test sets followed the same rule as described above.

The trained classifiers produced a probability for each anchor pair. We noted that using 0.5 as the cutoff to call positive pairs did not produce optimal F1 scores. Thus, based on the F1 scores on the validation sets, we selected the cut-offs that produced the best F1 scores for each dataset. We obtained the predicted probabilities scores for validation datasets, the samples with scores larger or equal than the cut-off value were assigned as predicted to be positive; the samples with scores smaller than the cut-off value were assigned as predicted to be negative. The F1 scores were calculated using Python function *sklearn.metrics.f1_score* with different selection of cut-off values (Table S10). The selected probability cutoffs were 0.22 for CTCF and 0.2 for RNA Pol II chromatin interaction predictions, respectively.

2.14 4C-seq library generation

MCF-7, a breast cancer cell line, was cultured in DMEM/F12 supplemented with 10% FBS and 1% penicillin-streptomycin and maintained at 37°C, 5% CO_2 humidified incubator.

MCF-7 cells were grown in hormone-free media: they were washed with PBS and incubated in phenol red-free medium (Invitrogen/Gibco) supplemented with 10% charcoal-dextran stripped FBS (Hyclone) and 1% pencillin-streptomycin for a minimum of 72 hours. Hormone-depleted MCF-7 cells were treated with oestrogen to a final concentration of 100 nM for 45 mins before 4C assay. The control cells were treated with an equal volume and concentration of vehicle, ethanol (ET, Merck), for 45 min. In Supplementary Figure 5b-df, only 4C interactions in control cells were shown. The interactions detected by 4C were similar between oestrogen-treated and control MCF-7 cells (Supplementary Figure 5e-g).

For both MCF-7 and K562 cell lines, 4C-seq assays were performed as previously described in Splinter et al.[13] with slight modifications. Briefly, 4×10^7 cells were cross-linked with 1% formaldehyde and the nuclei pellets were isolated after cell lysis with cold lysis buffer supplemented with protease inhibitors. First step digestion was performed overnight at 37°C with HindIII enzyme. Digestion efficiency was measured by RT-qPCR with HindIII site-specific primers. After phenol-chloroform extraction, DNA was ligated overnight at 16°C by T4 DNA lig-

ase. Following de-crosslinking, DNA was processed for second digestion with DpnII enzyme overnight at 37°C. After final ligation, 4C template DNA concentration was determined using fluorescence assay (picogreen, Invitrogen) and proceeded for library preparation for MiSeq sequencing using specific primers with Illumina Nextera adapters.

2.15 4C-seq data analysis

Primer sequences at 5' ends of reads were trimmed using Tagdust2[14]. Extracted reads were then mapped to reference genome (hg19) using Bowtie2 (2.2.6)[15]. Unaligned reads were collected. The first 50 base pairs of the unaligned reads were extracted and realigned to the genome using Bowtie2 (2.2.6). The uniquely aligned reads (MAPQ$\geq$ 30) were combined. R Package r3Cseq[16] was used to call significant interactions using non-overlapping window approach. 5kb windows were used.

2.16 RT-qPCR experiments

Total RNA were isolated from the cells using RNeasy Mini Kit (Qiagen) with on-column DNase digestion (Qiagen). 1μg of total RNA was then reverse transcribed to cDNA using the SuperScript III first-strand synthesis system using oligodT (Invitrogen). The expression levels of various genes were analysed by real-time PCR. Quantitative real-time PCR (qPCR) was performed on Applied Biosystems QuantStudio 3 Real-Time PCR system using SYBR Green PCR Master Mix and appropriate primers. The transcript levels of genes were analysed by $2^{-\Delta\Delta Ct}$ method.

2.17 Analysis of chronic lymphocytic leukemia samples

ATAC-seq peaks of chronic lymphocytic leukemia (CLL) samples were collected from Gene Expression Omnibus (GSE81274). Peaks from the CLL samples were pooled and merged with merging distance of 3000 bp. The merged peaks were then extended by 1000 bp at both sides. All potential pairs of merged peaks that were on the same chromosome and had separation (as measured by the distance between centers of the two peaks) in the range of 5 kb to 2 Mb were used. The pairs were then fed into the CTCF and RNA Pol II CHINN models with gradient boosted trees trained on genome-wide open chromatin pairs to obtain probabilities of interaction. The probability cutoffs were 0.22 for CTCF chromatin interactions and 0.2 for RNA Pol II chromatin interactions, which were selected for the gradient boosted trees trained on genome-wide open chromatin pairs as described above. For each CLL sample, its CTCF chromatin interactions and RNA Pol II chromatin

interactions were the predicted chromatin interactions whose both anchors overlap with peaks in the ATAC-seq library of the sample.

Random forest classifiers were trained using predicted CTCF and RNA Pol II chromatin interactions, respectively, to classify the samples into IGHV-mutated (mCLL) and IGHV-unmutated (uCLL) samples. Each sample is represented by a binary feature vector of the predicted chromatin interactions, where each value indicates whether the predicted chromatin interaction is present in the sample. We used the scikit-learn[17] implementation of random forest classifier with the parameter *n_estimator* set to 500. The performances were obtained by using 4-fold cross-validation with samples from the same patient always in the same group. Top 1000 important features for each trained random forest classifiers were then used for hierarchical clustering. Label-shuffled performances were obtained by training random forest classifiers on label-shuffled samples with the rest remain the same.

Differential chromatin interactions were obtained by using Fisher's Exact test to evaluate whether a chromatin interaction was more associated with any of the two CLL subtypes. The p-value threshold used was 0.01. The same Fisher's Exact test was applied to anchor peaks of the predicted chromatin interactions to test whether they were also more associated with any subtype of CLL. Differential chromatin interactions with both anchor peaks having p-values smaller than 0.01 were categorized as 'Both', those with only one anchor peak having p-value smaller than 0.01 were categorized as 'One-side', and those having neither anchor peak with p-value smaller than 0.01 were categorized as 'Neither'.

Two sets of CLL gene expression data were used: 1) a microarray dataset containing 76 mCLL samples and 58 uCLL samples from Gene Expression Omnibus (GSE69034); 2) a RNA-seq dataset containing 5 mCLL and 5 uCLL samples as an expression matrix downloaded from Gene Expression Omnibus (GSE81274). The RNA-seq dataset was from the same patient cohort as the ATAC-seq data. For the microarray dataset, limma[18] was used for differential gene expression analysis and genes with adjusted p-value smaller than 0.01 were selected. For the RNA-seq dataset, DESeq2[19] was used for differential gene expression analysis and genes with adjusted p-value smaller than 0.05 were selected.

Each predicted chromatin interaction i and each open chromatin region o was assigned a number p_i^s or p_o^s that indicates the percentage of samples of a subtype s it appeared in. The connectivity c_t^s of a transcription start site t in a subtype s was measured by taking the average of the percentages of samples in which its associated chromatin interactions appeared, which is

$$c_t^s = \sum_{i \in I} p_i^s / |I|$$

, where I is the set of predicted chromatin interactions that overlapped with the transcription start site s. The presence of a transcription start site in a subtype

was measured by the open chromatin region that was closest to the transcription start site. We then checked the fold changes of connectivity and presence of each transcription start site between uCLL and mCLL subtypes.

The Hi-C, RNA-seq, and ATAC-seq data of the six new CLL samples were deposited in GSE163896. For RNA-seq, kallisto (0.46.0)[20] was used to quantify the abundance of transcripts. The reference transcriptome used was Ensembl release 75. kallisto reports transcripts per million (TPM) for the transcripts. The TPM values were not nromalized across samples. For ATAC-seq, reads were aligned as paired-ends reads to hg19 genome assembly using BWA (0.7.17-r1188)[21]. The command used was bwa mem -M. Duplicates were marked and removed using MarkDuplicates function of Picard (2.22.2)[22] from GATK. Paired-end reads with either end unmapped were filtered using samtools (1.6)[23]. macs2 (2.2.6)[24] was used for peak calling with -BAMPE option. For Hi-C, BWA (0.7.17-r1188)[21] was used for aligning the paired-end reads to hg19 genome assembly. The command used was bwa mem -SP5M. The ENCODE Hi-C pipeline was used for filtering Hi-C alignments. Unmapped pairs and pairs with low quality mapping were removed. The pairs were deduplicated based on the mapping location of both ends. HiCCUPS from Juicer Tools (1.9.12)[25] was used for calling loops. Contact domains were detected using Arrowhead from Juicer Tools (1.9.12)[25].

3 Supplementary Figure Legends

3.1 Fig. S1: Characteristics of positive and negative samples in distance-matched datasets

Fig. S1: Characteristics of positive and negative samples in distance-matched datasets. The 'Pol2' in the figure represents 'RNA Pol II'. a, Comparison of the support of chromatin interactions based on whether both anchors (Both), only one anchor (XOR), or neither anchors (Neither) overlap withopen chromatin regions. The number in brackets indicates the number of chromatin interactions in the category. b, Distance distributions of positive and negative samples in distance-matched datasets.

3.2 Fig. S2: Convolutional layer 1 kernels of the five models that resemble known transcription factor binding motifs

Fig. S2: Convolutional layer 1 kernels of the five models that resemble known transcription factor binding motifs. The 'Pol2' in the figure represents 'RNA Pol II'.

3.3 Fig. S3: Sequence models on extended datasets

Fig. S3: Sequence models on extended datasets. The 'Pol2' in the figure represents 'RNA Pol II'. a-c, Precision-recall curves of sequence models on extended datasets using (a) sequence features and distance; (b) only sequence features; and (c) only distance. d, Across-sample performancesof the sequence models on extended datasets. e-g, The importance scores of sequence features extracted from both directions (F: forward; RC: reverse complement) of the two anchors (left and right) by models trained on different datasets. The orange horizontal lines indicate average importance scores of the features from the strand of the anchor. h, Top layer 3 kernels of CTCF models captured the CTCF motif. i, Performances of using only CTCF motif occurrences, motif orientations, and distance for prediction. j, Precision-recall curves of NN model (same structure as ChINN sequence-based models) on extended datasets using sequence features and distance.

3.4 Fig. S4: Hi-C sequence models on extended datasets

Fig. S4: Hi-C sequence models on extended datasets. a-c, Precision-recall curves of sequence models on extended datasets using (a) sequence features and distance;

(b) only sequence features; and (c) only distance. d, A cross-sample performances of the sequence models on extended datasets. e, The importance scores of sequence features extracted from both directions (F: forward; RC: reverse complement) of the two anchors (left and right) by models trained on Hi-C datasets. The orange horizontal lines indicate average importance scores of the features from the strand of the anchor. f, Top layer 3 kernels of CTCF models captured the CTCF motif. g, Precision-recall curves of NN model (same structure as ChINN sequence-based models) on extended Hi-C datasets using sequence features and distance.

3.5 Fig. S5: Selection of parameters for from-dnase models and validations by 4C-seq in MCF-7 cells

Fig. S5: Selection of parameters for from-dnase models and validations by 4C-seq in MCF-7 cells. The 'Pol2' in the figure represents 'RNA Pol II'. a, auROC of models trained on extended datasets based on different combinations of merging distances and extension sizes. b, Validations of predicted chromatin interactions by 4C-seq at GREB1 gene gions in MCF-7 cells. c, Validations of predicted chromatin interactions by 4C-seq at SIAH2 gene gions in MCF-7 cells. d, Validations of predicted chromatin interactions by 4C-seq at EVOVL2 gene gions in MCF-7 cells. e-g: Comparison of 4C-seq chromatin interactions between oestrogen -treated and control MCF-7 cells at ELOVL2, GREB1 and SIAH2 gene.

3.6 Fig. S6a-d: Selection of parameters for Hi-C models and validations by 4C-seq in MCF-7 cells

Fig. S6a-d: Selection of parameters for Hi-C models and validations by 4C-seq in MCF-7 cells. a, auROC of models trained on Hi-C extended datasets based on different combinations of merging distances and extension sizes. b, Validations of predicted chromatin interactions by 4C-seq at GREB1 gene gions in MCF-7 cells. c, Validations of predicted chromatin interactions by 4C-seq at SIAH2 gene gions in MCF-7 cells. d, Validations of predicted chromatin interactions by 4C-seq at EVOVL2 gene gions in MCF-7 cells.

3.7 Fig. S7: A diagram of user demands among different chromatin interactions prediction using sequences

Fig. S7: A diagram of user demands among different chromatin interactions prediction using sequences.

3.8 Fig. S8: The F-score with different thresholds for K562 and GM12878 Hi-C model on new CLL samples

Fig. S8: The F-score with different thresholds for K562 and GM12878 Hi-C model on new CLL samples. a, K562 Hi-C model. b, GM12878 Hi-C model.

3.9 Fig. S9: Performances of the sequence-based and DNase models in new CLL samples.

Fig. S9: Performances of the sequence-based and DNase models in new CLL samples. a, Precision-recall curves of the sequence-based models on distance-matched Hi-C datasets using sequence features with distance. b, Across-sample performances as measured by area-under precision-recall curve (auPRC) of the models on distance-matched Hi-C datasets using sequence features with distance. c, Significant motifs identified from the CLL models by the convolutional layer 1 kernels. Identified motifs were ranked according to the p-value from the smallest (top) to the largest (bottom). E-value ¡ 0.01. d, The importance scores of sequence features extracted from both directions (F: forward; RC: reverse complement) of the two anchors (left and right) by models trained on CLL samples. The orange horizontal lines indicate average importance scores of the features from the strand of the anchor. e, Pearson correlations between feature importance scores of the two anchors of CLL samples. f, Performances of the sequence models on extended datasets compared with random model in CLL samples. g, Across-sample performances of the sequence models on extended datasets in CLL samples. h, Performances of the DNase model compared with random model in CLL samples. i, Confusion matrixes of the models in h, with threshold of 0.016. x-axis: ture label, y-axis: predicted label. 0: negative, 1: positive. j, Across-sample performances of the DNase model in CLL samples. k, Confusion matrixes of the results of using CLL 401 model to predict chromatin interactions in other samples with threshold of 0.016. x-axis: ture label, y-axis: predicted label. 0: negative, 1: positive. l-o, Validations of predicted chromatin interactions by 4C-seq at *ELOVL2*, *CAP2*, *P2RY2*, and *TP53* gene regions in MCF-7 cells. p-q, Association of differences in real Hi-C (p) and predicted Hi-C (q) chromatin interactions between uCLL and mCLL samples with differentially expressed genes from RNA-seq. IFC: the fold change of the average number of chromatin interactions observed at the gene promoter in uCLL samples over that in mCLL samples. r, ATAC-seq peaks, Hi-C interactions, and RNA-seq signal around KCNJ11 gene.

3.10 Fig. S10: Analyses of predicted chromatin interactions in CLL samples using GM12878 Hi-C model

Fig. S10: Analyses of predicted chromatin interactions in CLL samples using GM12878 Hi-C model. a, Heatmap of hierarchical clustering of CLL samples based on the 1000 most important chromatin interactions of the Hi-C random forest classiers. Yellow indicates the presence of a chromatin interaction and black indicates the absence of a chromatin interaction. Red color represents mCLL samples and blue represents uCLL samples. b, Differences in agreement between anchors of differential Hi-C chromatin interactions in the 'Neither' category between mCLL and uCLL samples. c, Association of differences in chromatin interactions between uCLL and mCLL samples with differentially expressed genes identifed using RNA-seq data. IFC: the fold change of the average number of chromatin interactions observed at the gene promoter in uCLL samples over that in mCLL samples. p-values were calculated using Kruskal-Wallis test. d, The association between differences in chromatin interactions and differences in open chromatin peaks at gene promoter between uCLL and mCLL samples. Left: mutated, right: unmutated. PFC: the fold change of the proportions of samples the open chromatin peak at gene promoter is observed in uCLL samples over that in mCLL samples. The color barindicates the -log(pvalue) of the most significantly differential chromatin interaction at the promoter. e-h, Examples of genes whose different connectivity are associated with differences in distal regions. The red bars and curves indicate significantly different open chromatin regions and chromatin interactions based on Fisher's Exact test.

3.11 Fig. S11: Analyses of predicted chromatin interactions in CLL samples

Fig. S11: Analyses of predicted chromatin interactions in CLL samples. The 'Pol2' in the figure represents 'RNA Pol II'. a, Heatmaps of hierarchical clustering of CLL samples based on the 1000 most important chromatin interactions of the CTCF and RNA Pol II random forest classifiers, respectively. Yellow indicates the presence of a chromatin interaction and black indicates the absence of a chromatin interaction. Red color represents mCLL samples and blue represents uCLL samples. b, Differences in agreement between anchors of differential CTCF and RNA Pol II chromatin interactions in the 'Neither' category between mCLL and uCLL samples. c, Association of differences in chromatin interactions between uCLL and mCLL samples with differentially expressed genes identified using RNA-seq data. IFC: the fold change of the average number of chromatin interactions observed at the gene promoter in uCLL samples over that in mCLL samples. p-values were calculated using Kruskal-Wallis test. d, The association between differences in

chromatin interactions and differences in open chromatin peaks at gene promoter between uCLL and mCLL samples. PFC: the fold change of the proportions of samples the open chromatin peak at gene promoter is observed in uCLL samples over that in mCLL samples. The color bar indicates the -log(pvalue) of the most significantly differential chromatin interaction at the promoter. e-h, Examples of genes whose different connectivity are associated with differences in distal regions. The red bars and curves indicate significantly different open chromatin regions and chromatin interactions based on Fisher's Exact test.

3.12 Fig. S12: Per base quality of CLL RNA-seq libraries

Fig. S12: Per base quality plot from fastQC of the CLL RNA-seq libraries. The sample name are indicated in each panel. The per base quality plot of read1 and read2 are located at the left and right side, respectively.

4 Supplementary Table Legends

4.1 Table S1: Description and comparison among Akita, DeepC, DeepTACT, and SEPT.

4.2 Table S2: Negative-to-positive ratios of samples on each chromosome of distance-matched datasets.

4.3 Table S3: List of ChIP-seq peaks downloaded from ENCODE.

4.4 Table S4: Convolutional layer 1 kernels of the eight Hi-C models from cell lines that resemble known transcription factor binding motifs.

4.5 Table S5: The number of cell lines for each motif that was detected in eight Hi-C models

4.6 Table S6: Clinical characteristics of the CLL samples

4.7 Table S7: Hi-C libraries summary of CLL samples

4.8 Table S8: ATAC-seq libraries summary of CLL samples

4.9 Table S9: Primer information

4.10 Table S10: F1-score calculated with different cutoff values from pairs of open chromatin regions in GM12878 cells.

5 Supplementary Figures

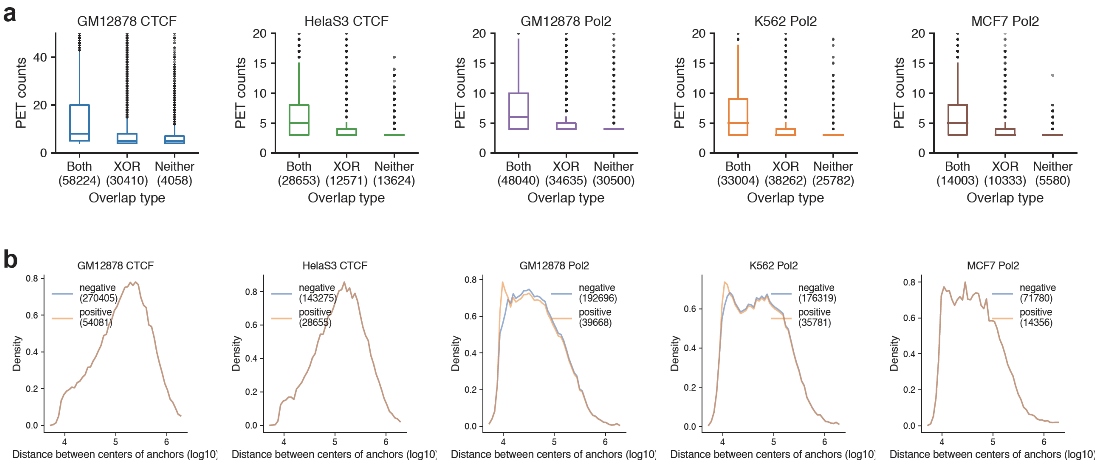

a
GM12878 CTCF
HelaS3 CTCF
GM12878 Pol2
K562 Pol2
MCF7 Pol2
PET counts
Both (58224) XOR (30410) Neither (4058)
Both (28653) XOR (12571) Neither (13624)
Both (48040) XOR (34635) Neither (30500)
Both (33004) XOR (38262) Neither (25782)
Both (14003) XOR (10333) Neither (5580)
Overlap type
b
Density
negative (270405) positive (54081)
negative (143275) positive (28655)
negative (192696) positive (39668)
negative (176319) positive (35781)
negative (71780) positive (14356)
Distance between centers of anchors (log10)

Fig. S1

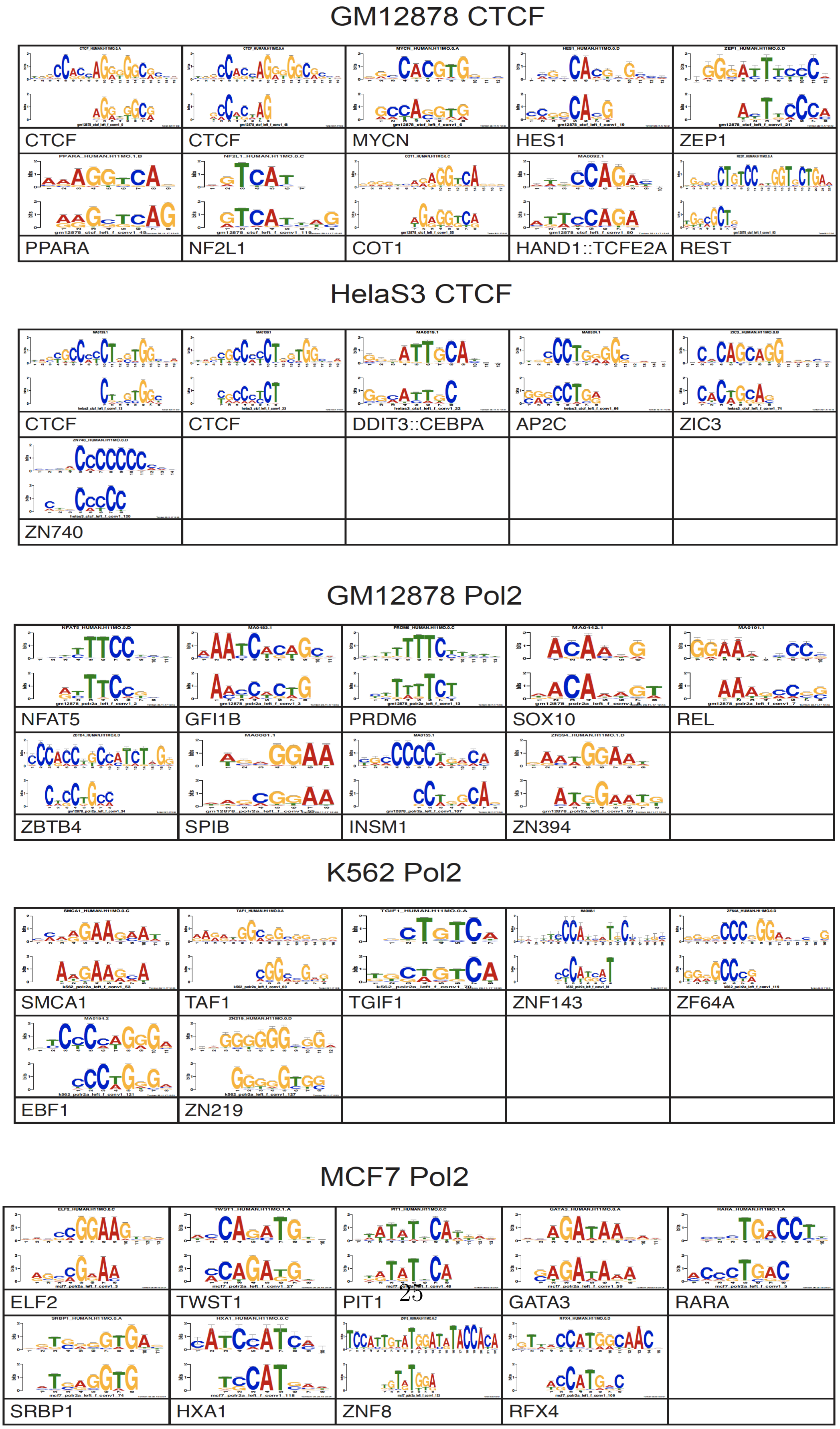
GM12878 CTCF
CTCF
CTCF
MYCN
HES1
ZEP1
PPARA
NF2L1
COT1
HAND1::TCFE2A
REST
HelaS3 CTCF
CTCF
CTCF
DDIT3::CEBPA
AP2C
ZIC3
ZN740
GM12878 Pol2
NFAT5
GFI1B
PRDM6
SOX10
REL
ZBTB4
SPIB
INSM1
ZN394
K562 Pol2
SMCA1
TAF1
TGIF1
ZNF143
ZF64A
EBF1
ZN219
MCF7 Pol2
ELF2
TWST1
PIT1
GATA3
RARA
SRBP1
HXA1
ZNF8
RFX4

Fig. S2

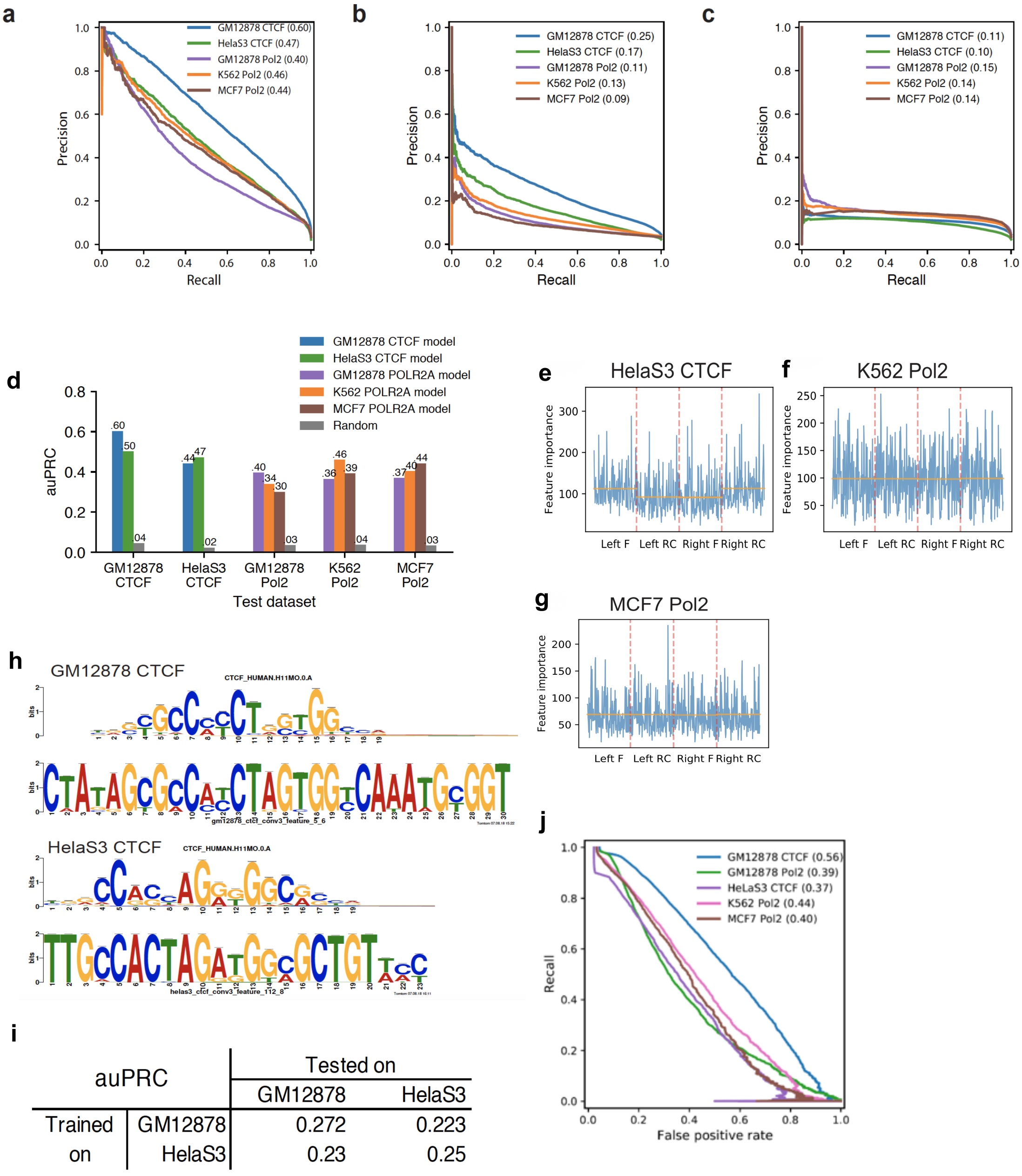

auPRC		Tested on GM12878	Tested on HelaS3
Trained on	GM12878	0.272	0.223
	HelaS3	0.23	0.25

Fig. S3

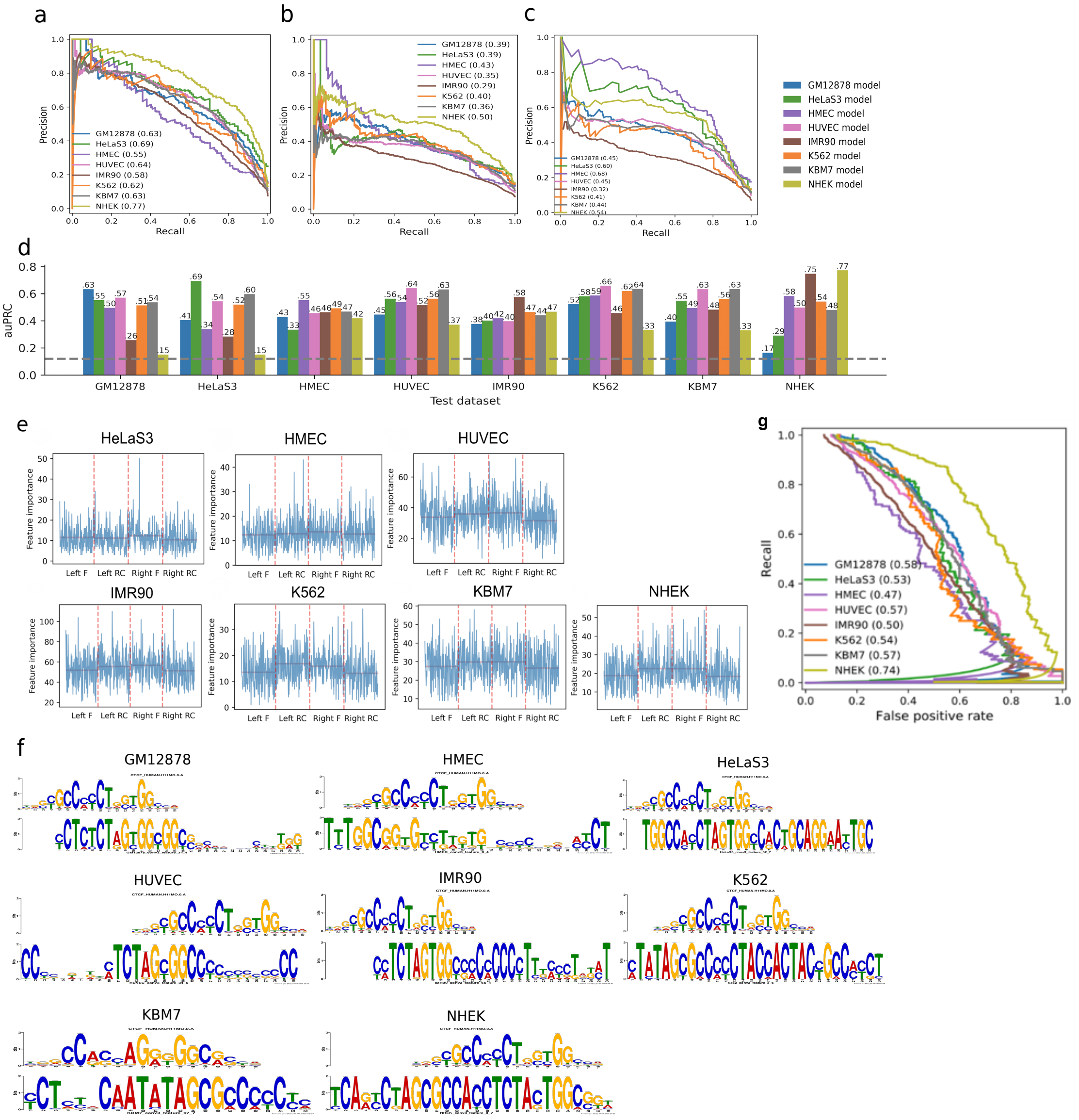
a
b
c
d
e
f
g
Precision
Recall
GM12878 (0.63)
HeLaS3 (0.69)
HMEC (0.55)
HUVEC (0.64)
IMR90 (0.58)
K562 (0.62)
KBM7 (0.63)
NHEK (0.77)
GM12878 (0.39)
HeLaS3 (0.39)
HMEC (0.43)
HUVEC (0.35)
IMR90 (0.29)
K562 (0.40)
KBM7 (0.36)
NHEK (0.50)
GM12878 model
HeLaS3 model
HMEC model
HUVEC model
IMR90 model
K562 model
KBM7 model
NHEK model
auPRC
Test dataset
GM12878
HeLaS3
HMEC
HUVEC
IMR90
K562
KBM7
NHEK
Feature importance
Left F
Left RC
Right F
Right RC
Recall
False positive rate
GM12878 (0.58)
HeLaS3 (0.53)
HMEC (0.47)
HUVEC (0.57)
IMR90 (0.50)
K562 (0.54)
KBM7 (0.57)
NHEK (0.74)

Fig. S4

a

CTCF auROC	Extension size (bp)					
Merging size (bp)	500	1000	1500	2000	2500	3000
500	0.933	0.942	0.948	0.952	0.953	0.952
1000	0.944	0.946	0.95	0.954	0.954	0.954
1500	0.95	0.952	0.951	0.953	0.953	0.953
2000	0.953	0.955	0.954	0.954	0.953	0.953
2500	0.954	0.956	0.956	0.954	0.953	0.953
3000	0.955	0.957	0.956	0.955	0.954	0.952

Pol2 auROC	Extension size (bp)					
Merging size (bp)	500	1000	1500	2000	2500	3000
500	0.926	0.932	0.934	0.934	0.934	0.934
1000	0.937	0.94	0.94	0.939	0.939	0.938
1500	0.943	0.946	0.946	0.945	0.945	0.944
2000	0.947	0.948	0.948	0.947	0.947	0.946
2500	0.953	0.954	0.953	0.953	0.952	0.951
3000	0.954	0.954	0.953	0.953	0.952	0.951

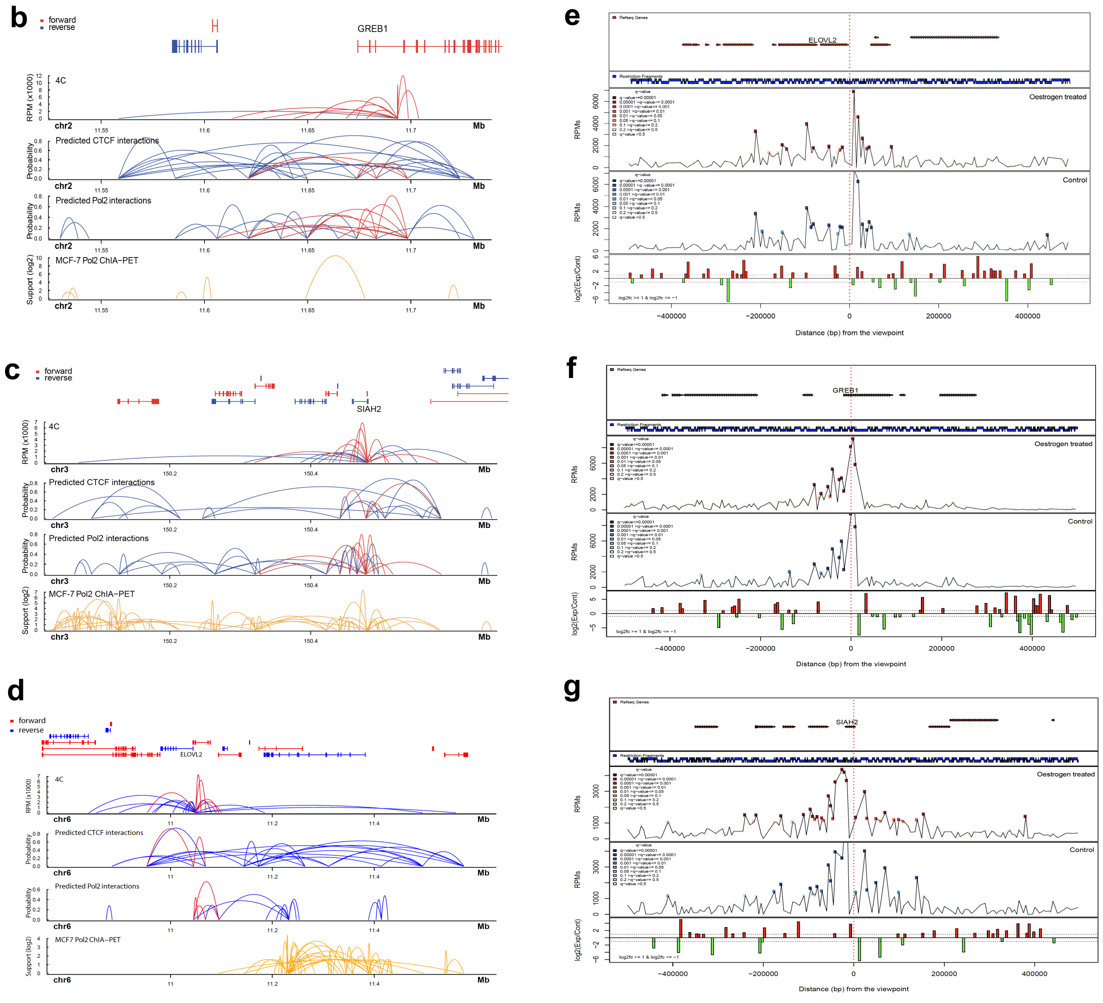

Fig. S5

a

K562 auROC		merge size 500	1000	2000	3000	4000	5000
extension size	500	0.8554	0.8549	0.7691	0.8582	0.8594	0.8543
	1000	0.8668	0.8654	0.8657	0.8693	0.8679	0.8652
	2000	0.8743	0.8769	0.8795	0.8765	0.8766	0.8747
	3000	0.8732	0.8809	0.8816	0.8775	0.8772	0.8758

GM12878 auROC		merge size 500	1000	2000	3000	4000	5000
extension size	500	0.8157	0.8129	0.8192	0.821	0.8257	0.8314
	1000	0.8061	0.8027	0.8069	0.8083	0.8159	0.8214
	2000	0.778	0.7778	0.7895	0.7904	0.7978	0.8039
	3000	0.7502	0.7477	0.7551	0.758	0.762	0.7687

b

c

d

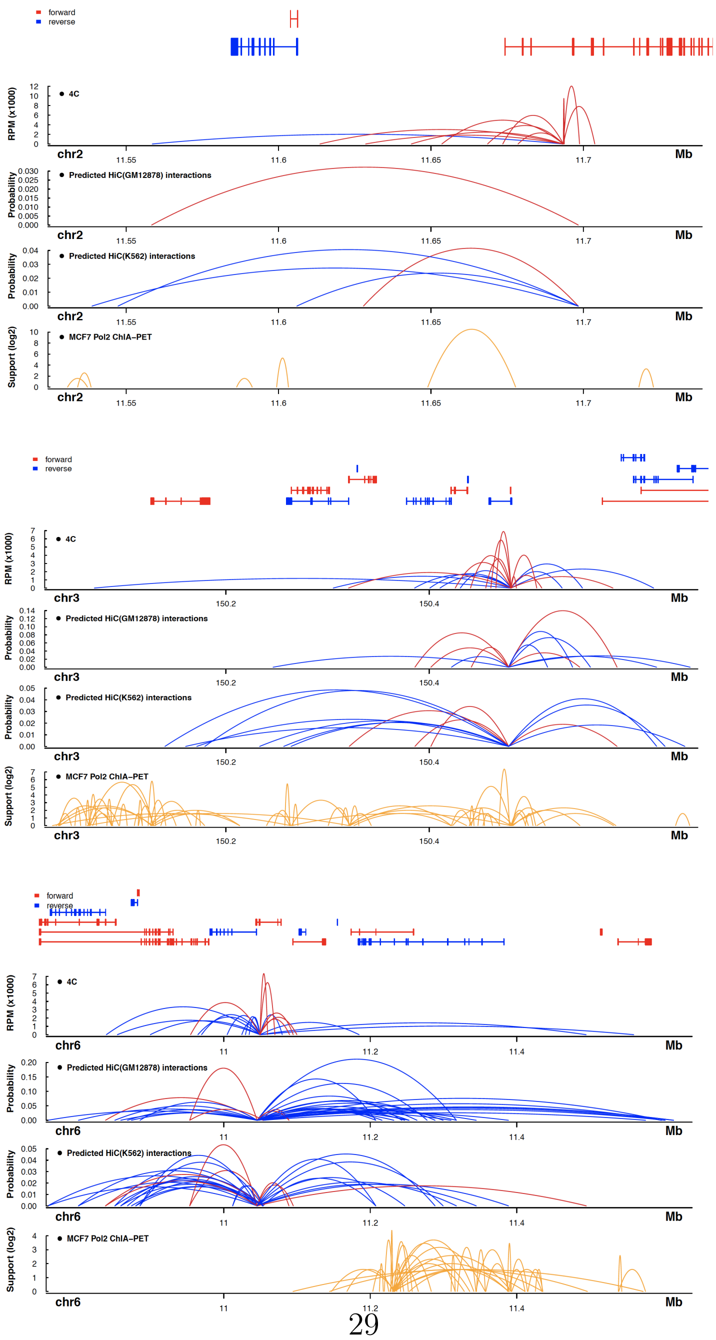

Fig. S6

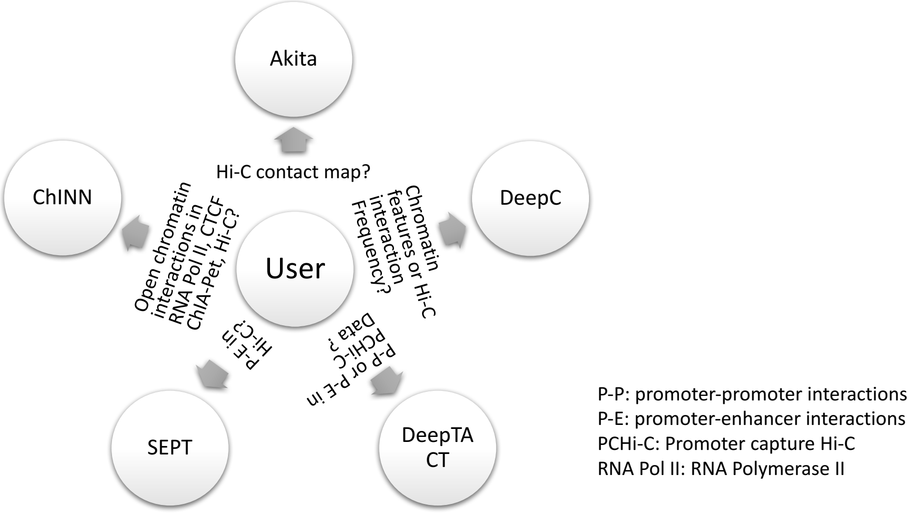

Fig. S7

a

K562 F-score	threshold												
cll sample	0.5	0.4	0.3	0.2	0.1	0.03	0.025	0.02	0.017	0.016	0.015	0.014	0.01
102	0.0017	0.0034	0.0076	0.0241	0.0929	0.2989	0.3169	0.3297	0.3351	0.3351	0.3325	0.33	0.3182
312	0.0005	0.0021	0.0077	0.036	0.1315	0.3618	0.3811	0.3964	0.4094	0.4122	0.4121	0.4101	0.3953
324	0.0013	0.0019	0.0076	0.0351	0.1409	0.3797	0.3979	0.419	0.4223	0.4229	0.422	0.4197	0.403
344	0.0006	0.0006	0.005	0.0186	0.0726	0.2426	0.2669	0.2947	0.3067	0.3138	0.3157	0.3162	0.322
401	0.0009	0.0015	0.0043	0.0177	0.0773	0.2516	0.2704	0.2845	0.2866	0.2894	0.29	0.2922	0.2894
484	0.0007	0.0014	0.0036	0.0146	0.0678	0.2567	0.2859	0.3132	0.3316	0.3394	0.3449	0.3484	0.3625

b

GM12878 F-score	threshold												
cll sample	0.5	0.4	0.3	0.2	0.1	0.03	0.028	0.027	0.025	0.024	0.02	0.015	0.01
102	0.0013	0.0025	0.0138	0.0565	0.2025	0.3843	0.385	0.3846	0.3804	0.3779	0.3659	0.3536	0.3338
312	0.0005	0.0036	0.0173	0.0705	0.2388	0.3841	0.3812	0.3816	0.382	0.3818	0.3696	0.3595	0.3406
324	0.0013	0.0044	0.0206	0.0813	0.2656	0.4455	0.4465	0.4466	0.4484	0.4473	0.4354	0.4089	0.3685
344	0	0.0023	0.0134	0.0478	0.163	0.2958	0.3027	0.3034	0.3063	0.3087	0.3143	0.3179	0.318
401	0.0006	0.0025	0.0152	0.0535	0.1674	0.335	0.3365	0.3373	0.3368	0.3354	0.3284	0.326	0.319
484	0.0007	0.0036	0.0126	0.0453	0.1546	0.3328	0.3417	0.3448	0.3518	0.355	0.37	0.3856	0.3931

Fig. S8

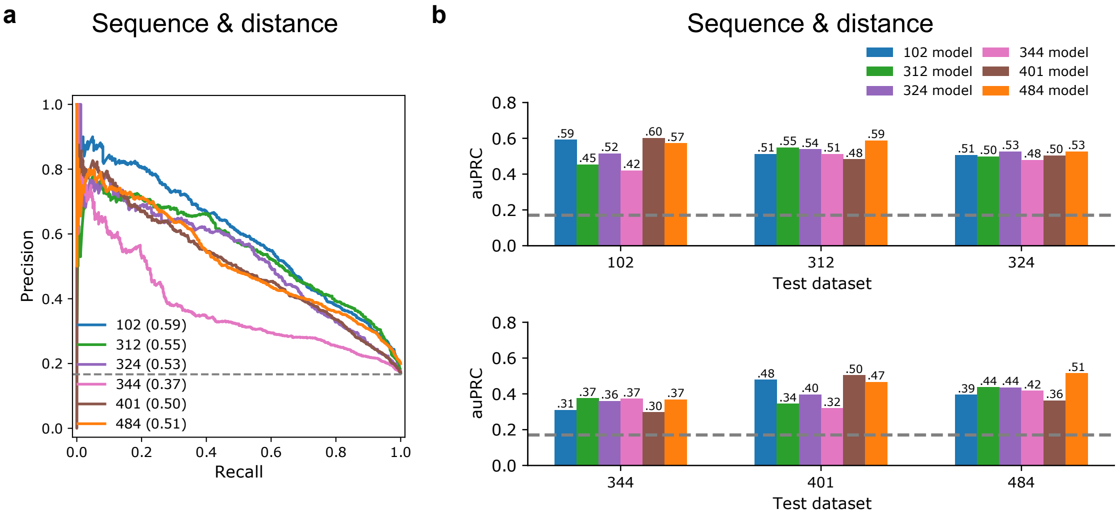

c

	102	312	324	344	401	484
	CTCF	CTCF	CTCF	CTCF	CTCF	CTCF
	CTCFL	CTCFL	CTCFL	CTCFL	CTCFL	CTCFL
	FOS	FOS	FOS	FOS	FOS	FOS
	JUNB	JUNB	JUNB	JUNB	JUNB	JUNB
	FOSL1	FOSL1	FOSL1	FOSL1	FOSB	FOSL1
	JUN	JUN	JUN	JUN	FOSL1	JUN
	JUND	JUND	JUND	JUND	JUN	JUND
	NFE2	FOSL2	FOSB	FOSL2	JUND	FOSL2
	FOSB	FOSB	FOSL2	FOSB	NFE2	FOSB
	BACH2	NF2L2	NFE2	NFE2	BACH2	NFE2
	MAFK	NFE2	ZN436	NF2L2	MAFK	NF2L2
	FOSL2	BACH2	NF2L2	BACH2	BACH1	BACH2
	BACH1	MAFK	BACH2	ZN436	FOSL2	BACH1
	NF2L2	BACH1	MAFK	MAFK	ZN436	MAFK
	ZN436	ZN436	BACH1	BACH1	NF2L2	ZN436
Motif	INSM1	TFE3	BMAL1	BMAL1	INSM1	IRF4
		TFEB	CR3L1	CR3L1		IRF8
		USF1	HES5	HES5		CR3L1
		USF2	HES7	HES7		MYCN
		CR3L1	HEY1	HEY1		BMAL1
		HES5	HEY2	MITF		HES5
		HES7	MITF	MYC		HES7
		HEY1	MYC	MYCN		HEY1
		MITF	MYCN	TFE3		HEY2
		MYC	TFE3	USF1		MITF
		MYCN	USF1	HEY2		MXI1
		BMAL1	INSM1	TFEB		MYC
		MAFG	MAX	USF2		TFE3
		INSM1	MXI1	MXI1		USF1
			TFEB	MAX		
			USF2			

Fig. S9 a-c

d

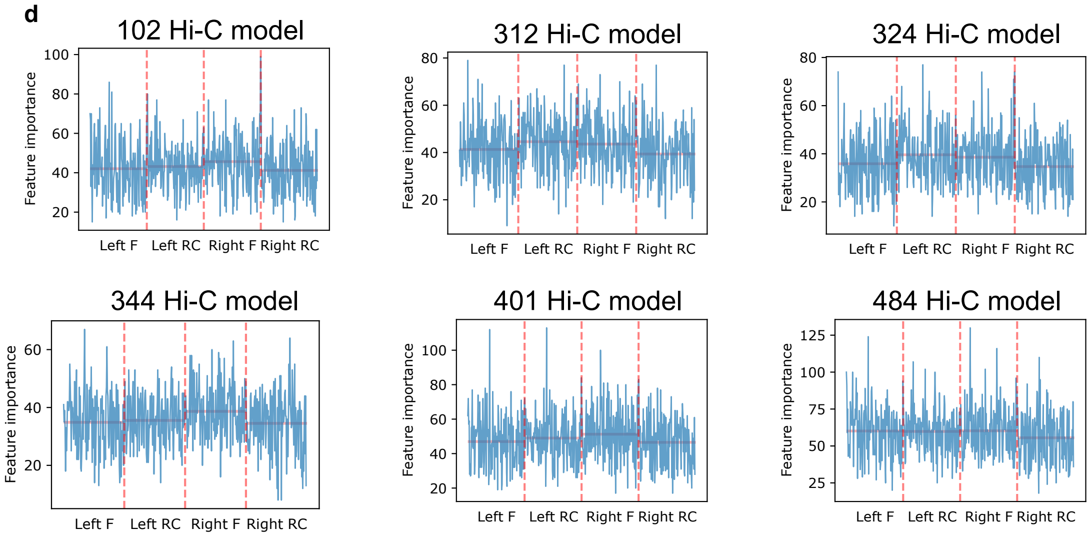

e

102 Hi-C model

	Right F	Right RC
Left F	0.562181	0.584517
Left RC	0.510532	0.480287

312 Hi-C model

	Right F	Right RC
Left F	0.380838	0.52689
Left RC	0.452727	0.366889

324 Hi-C model

	Right F	Right RC
Left F	0.444549	0.51084
Left RC	0.472598	0.387157

344 Hi-C model

	Right F	Right RC
Left F	0.384301	0.571981
Left RC	0.386465	0.416936

401 Hi-C model

	Right F	Right RC
Left F	0.555876	0.658608
Left RC	0.489294	0.577774

484 Hi-C model

	Right F	Right RC
Left F	0.606085	0.720893
Left RC	0.58604	0.628004

Fig. S9 d-e

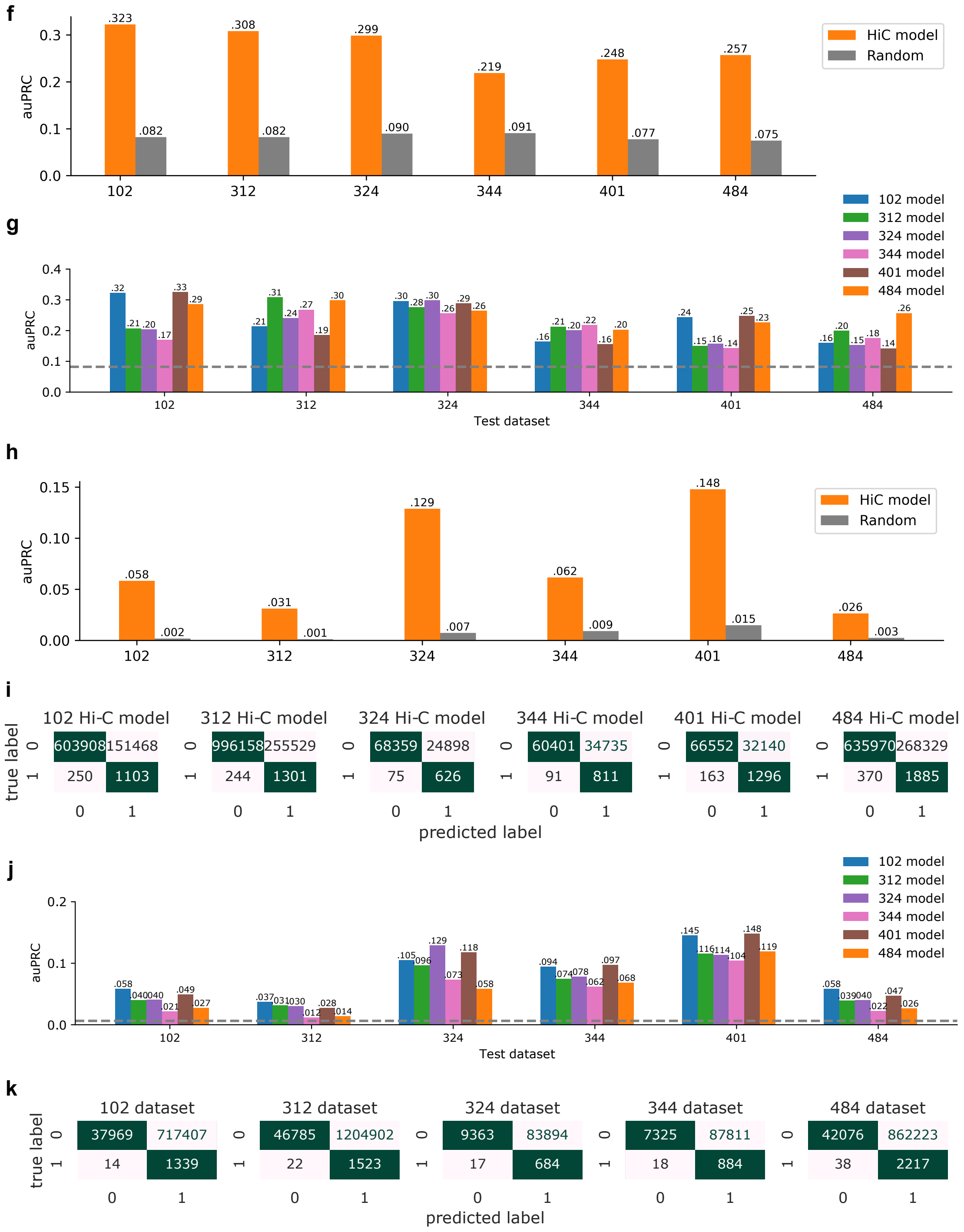

Fig. S9 f-k

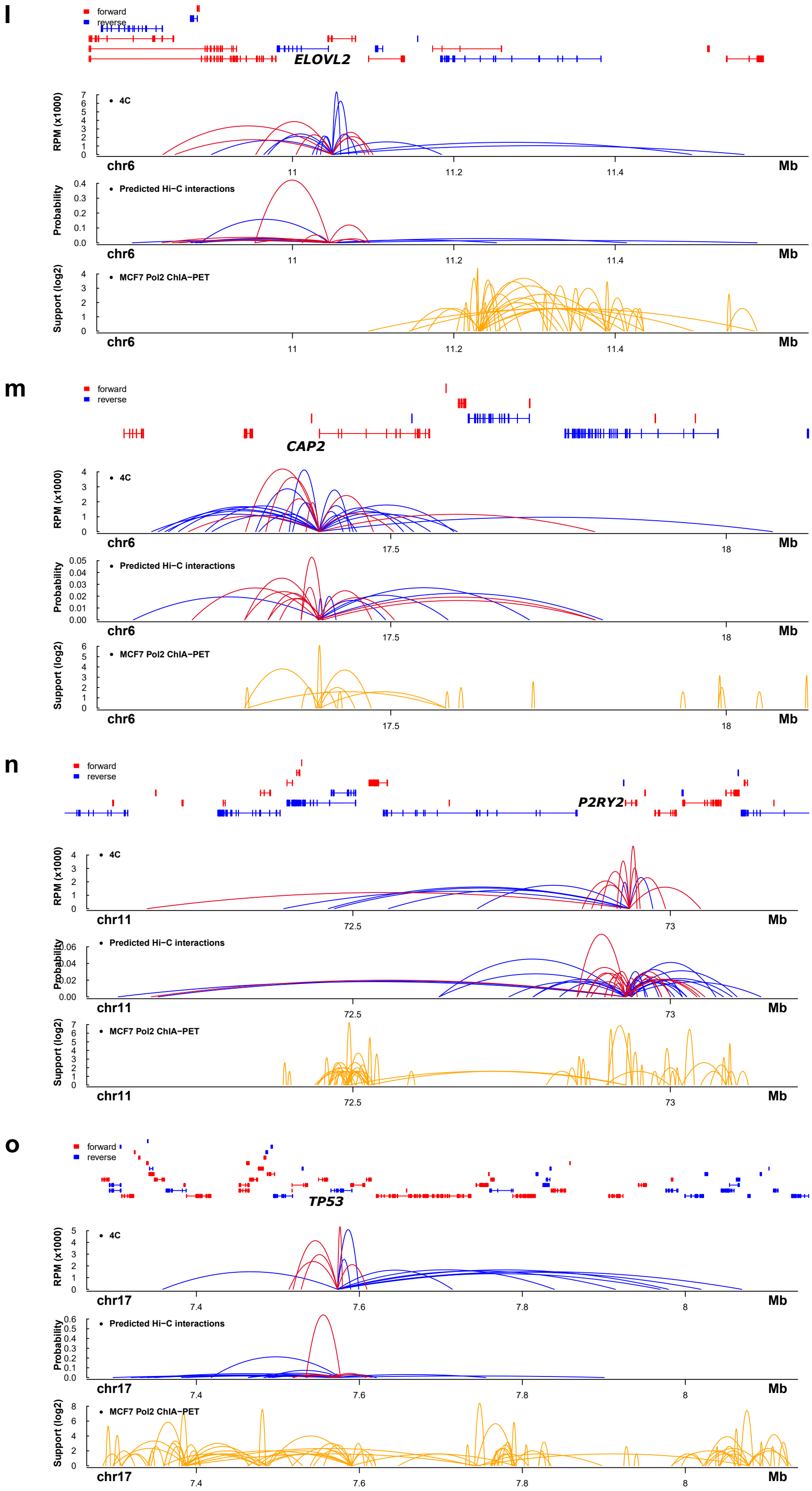
l
forward
reverse
ELOVL2
4C
RPM (x1000)
chr6
11
11.2
11.4
Mb
Predicted Hi−C interactions
Probability
MCF7 Pol2 ChIA−PET
Support (log2)
m
CAP2
17.5
18
n
P2RY2
chr11
72.5
73
o
TP53
chr17
7.4
7.6
7.8
8

Fig. S9 l-o

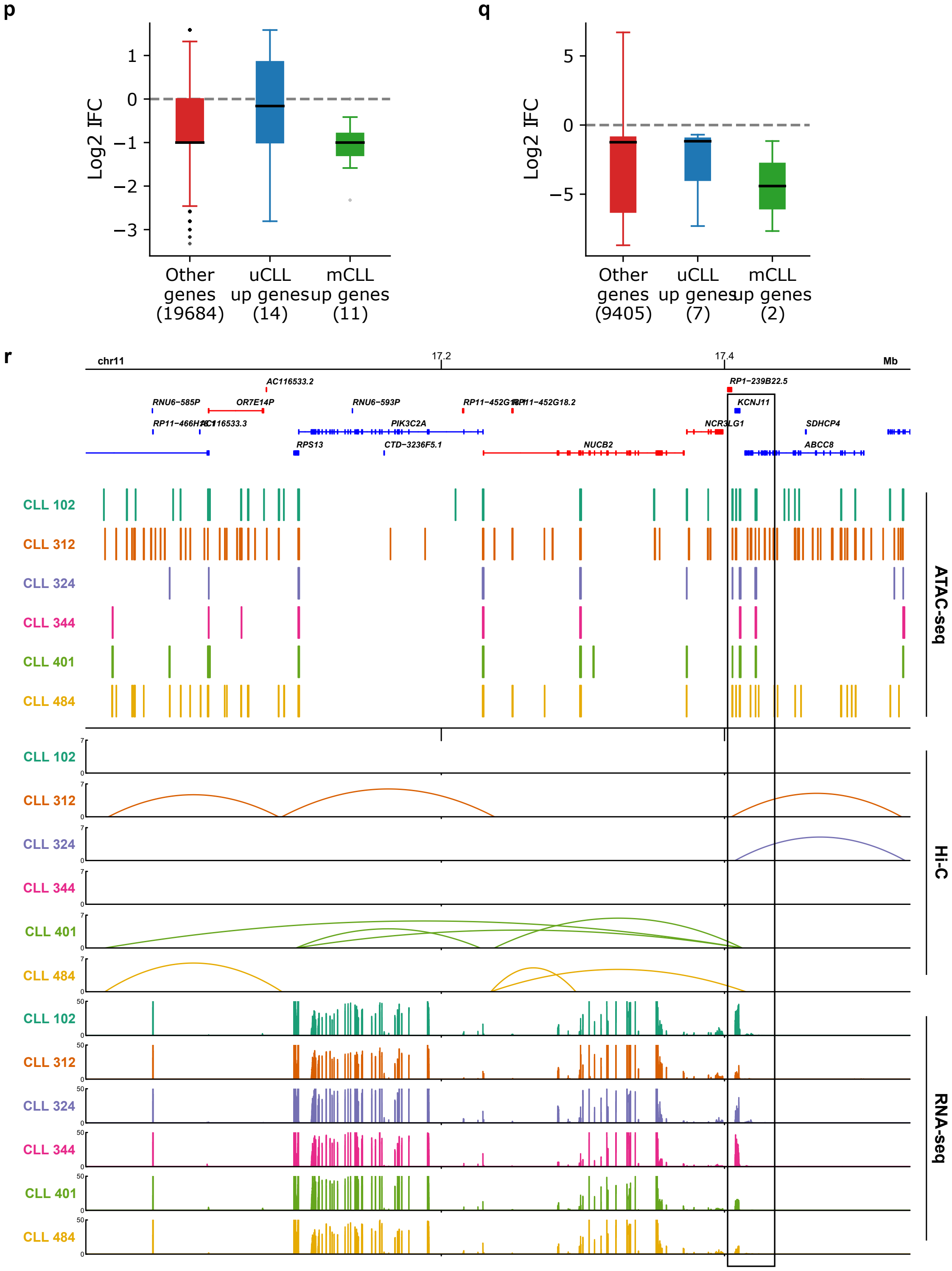
p
Log2 FC
Other genes (19684)
uCLL up genes (14)
mCLL up genes (11)
q
Log2 FC
Other genes (9405)
uCLL up genes (7)
mCLL up genes (2)
r
chr11
17.2
17.4
Mb
AC116533.2
RNU6-585P
OR7E14P
RNU6-593P
RP1-239B22.5
KCNJ11
PIK3C2A
SDHCP4
RPS13
CTD-3236F5.1
NUCB2
ABCC8
CLL 102
CLL 312
CLL 324
CLL 344
CLL 401
CLL 484
ATAC-seq
Hi-C
RNA-seq

Fig. S9 p-r

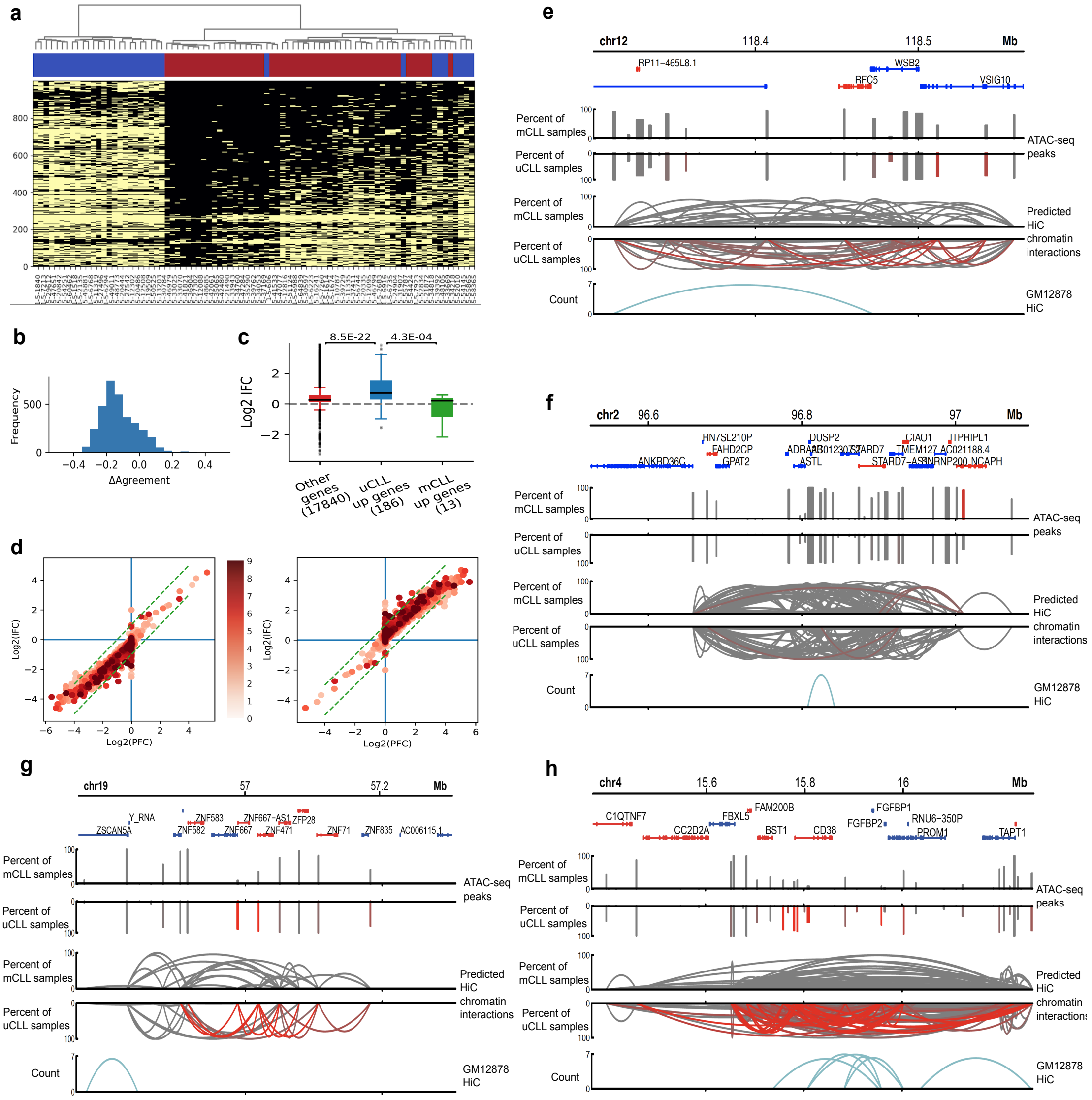
a
b
Frequency
ΔAgreement
c
Log2 IFC
8.5E-22
4.3E-04
Other genes (17840)
uCLL up genes (186)
mCLL up genes (13)
d
Log2(IFC)
Log2(PFC)
e
chr12
118.4
118.5
Mb
Percent of mCLL samples
Percent of uCLL samples
ATAC-seq peaks
Predicted HiC chromatin interactions
Count
GM12878 HiC
f
chr2
96.6
96.8
97
Mb
g
chr19
57
57.2
Mb
h
chr4
15.6
15.8
16
Mb

Fig. S10

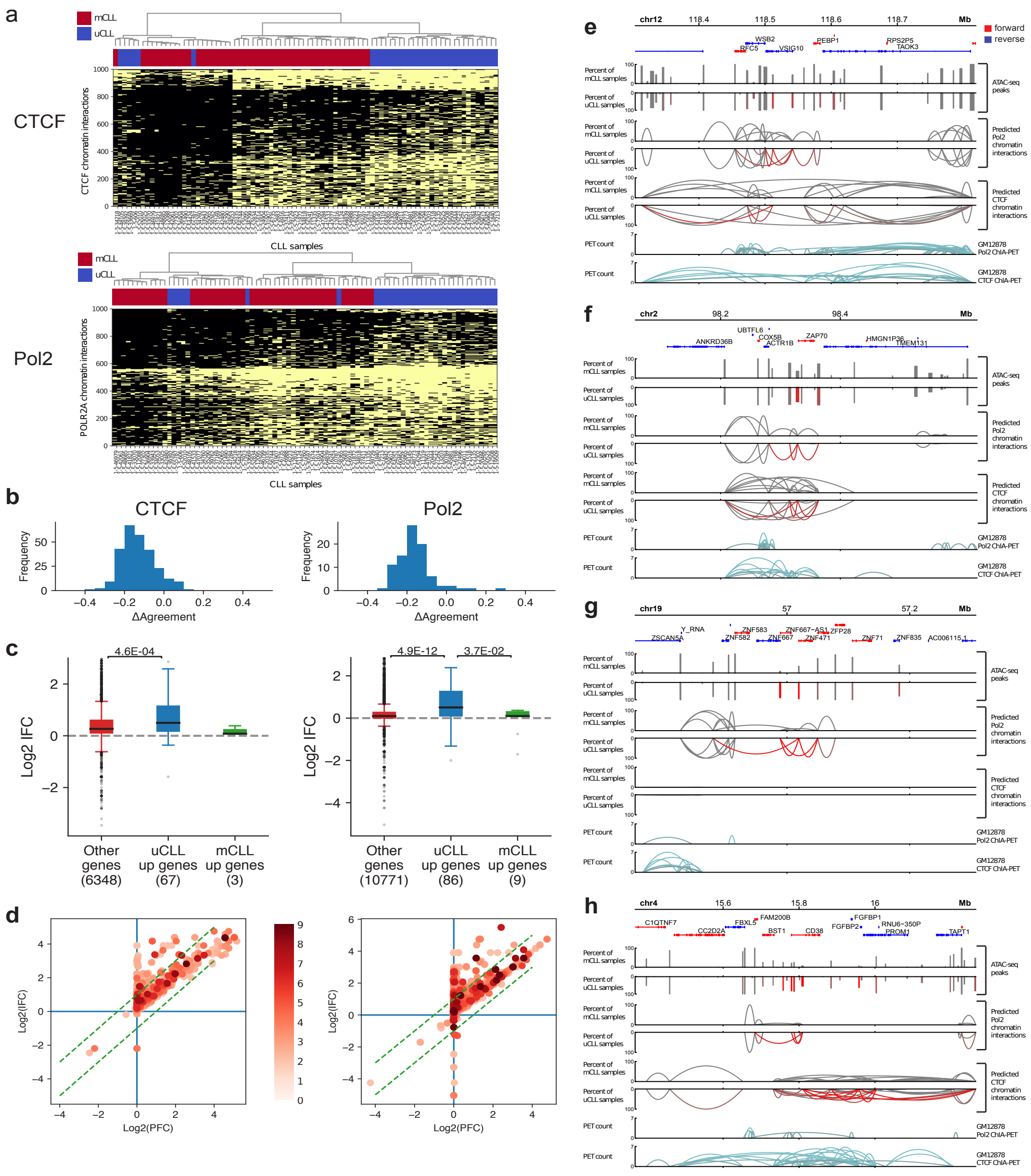

a
CTCF
Pol2
mCLL
uCLL
CLL samples
CTCF chromatin interactions
POLR2A chromatin interactions
b
CTCF
Pol2
Frequency
ΔAgreement
c
4.6E-04
4.9E-12
3.7E-02
Log2 IFC
Other genes (6348)
uCLL up genes (67)
mCLL up genes (3)
Other genes (10771)
uCLL up genes (86)
mCLL up genes (9)
d
Log2(IFC)
Log2(PFC)
e
chr12
Mb
forward
reverse
Percent of mCLL samples
Percent of uCLL samples
ATAC-seq peaks
Predicted Pol2 chromatin interactions
Predicted CTCF chromatin interactions
PET count
GM12878 Pol2 ChIA-PET
GM12878 CTCF ChIA-PET
f
chr2
g
chr19
h
chr4

Fig. S11

Sample: 102

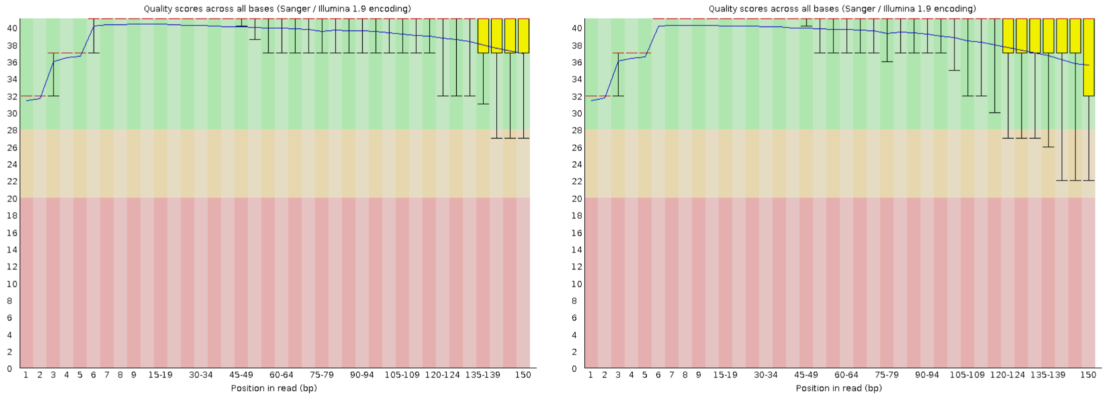

Sample: 312

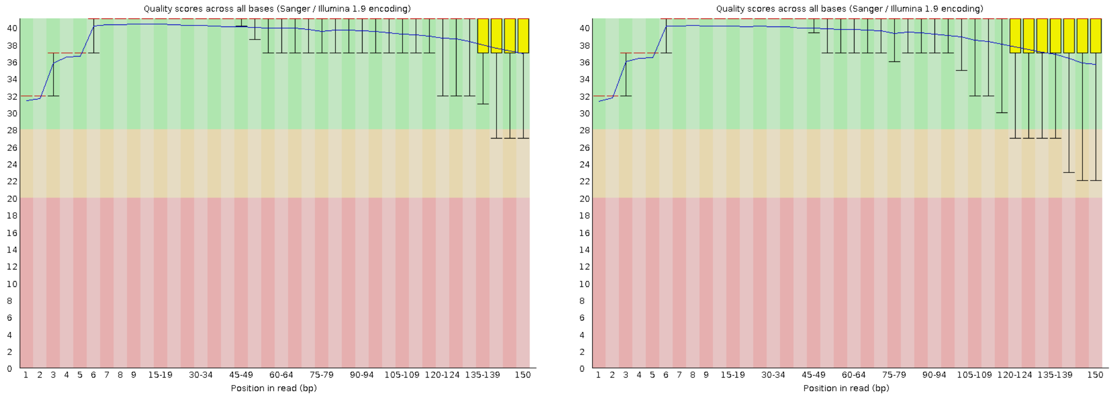

Sample: 324

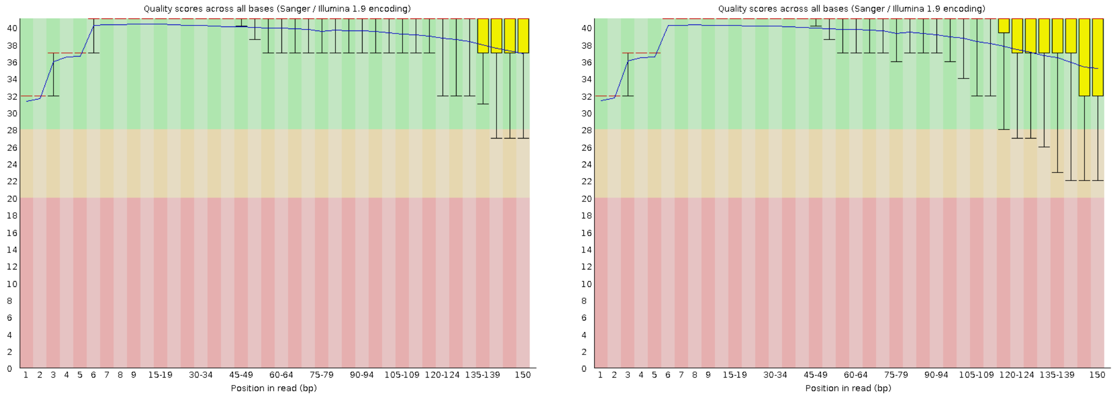

Fig. S12

Sample: 344

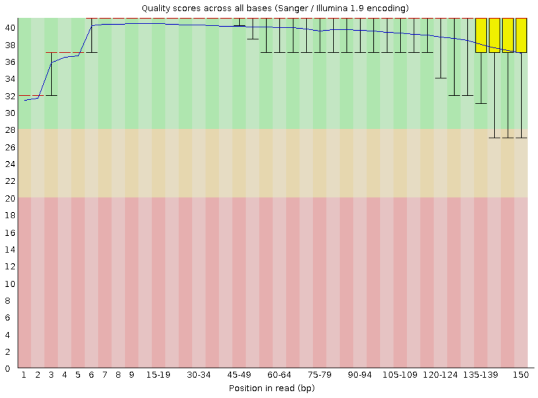

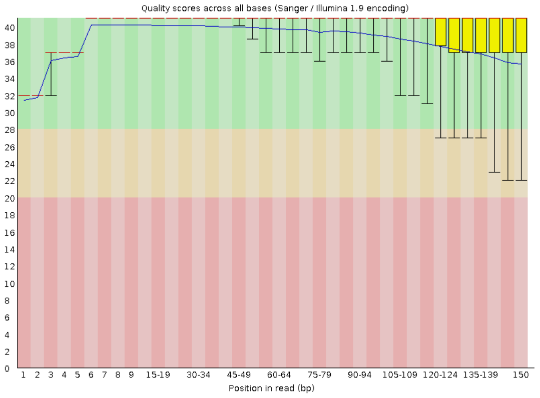

Sample: 401

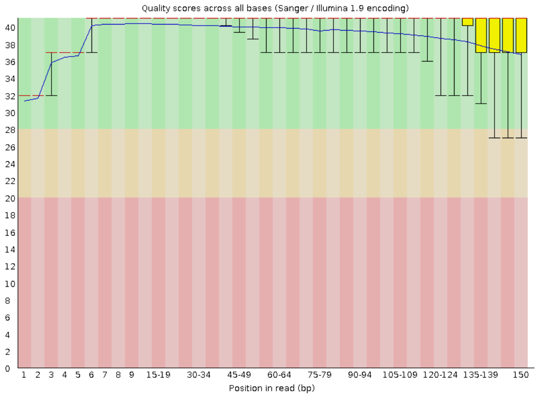

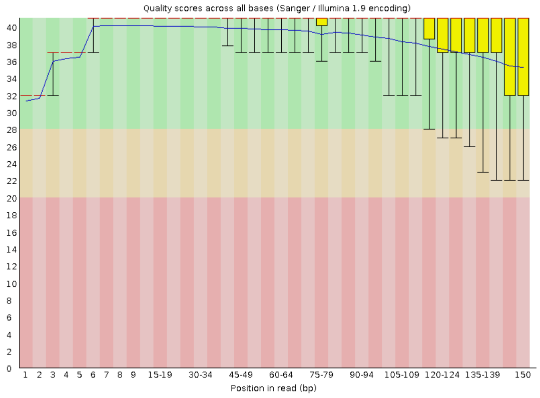

Sample: 484

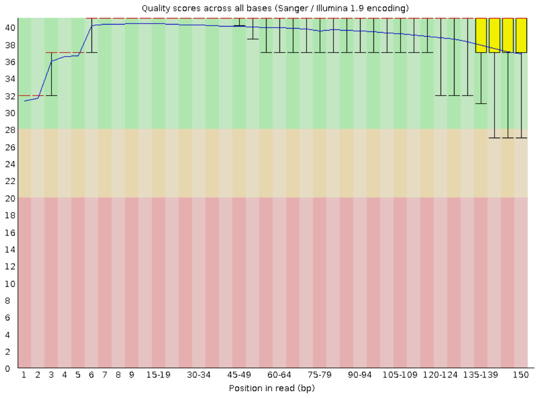

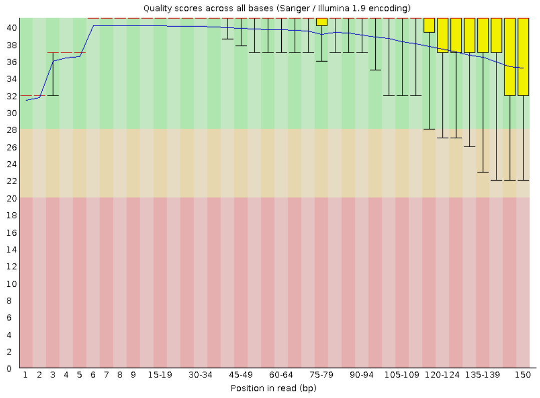

Fig. S12 (continue)

6 Supplementary Tables

Table S1

Method	Input	Output	Framework	Train/test/validation	Comments
Akita	1 Mb DNA sequences converted into bins (2048bp) (including distance in the input vector); Target: Hi-C matrix of 448*448 bin (2048 bp bin size; ~917 kb)	Hi-C contact map (2048 bp bin size; non-overlapping regions/contigs)	Convolutional neural network (CNN)	80/10/10 training/validation/test split	Only bed format regions are taken as input.
DeepC	1) Predict chromatin features (ATAC-seq, CTCF ChIP-seq, histone modifications, TF) from 1 kb of DNA sequence; 2) DNA sequence of 1Mb; Target: Hi-C interaction frequency of (binSize +- 1Mb) as target vector	Encoded Hi-C vector (Hi-C matrix)	Transfer learning	1) chromatin feature network: chr11, chr12 for validation; and chr15, chr16 and chr17 for testing; 2) chromatin interaction network: chr11, chr12 for validation; and chr16 and chr17 for testing	Only bed format region are take as input. (output only at this region); They also downsampled 1 billion, 100 million and 10 million valid Hi-C contacts from ~2.6 billion. 10 mil failed, while 100 mil and 1bil can capture (~0.4 pearson correlation)
DeepTACT	One-hot encoding for 2 kb or 1kb DNA sequences surrounding middle site; Target: Promoter capture Hi-C (PCHi-C)	predicted score	Convolutional neural network (CNN) + Recurrent Neural Network (RNN)	PCHi-C data and matching DNase-seq data in six cell types (total B cells (tB), monocytes (Mon), fetal thymus (FoeT), total CD4+ T cells (tCD4), naive CD4+ T cells (nCD4), total CD8+ T cells (tCD8)) to train and evaluate	Developed to predict promoter-promoter interactions (P-P) and promoter-enhancer interactions (P-E) in PCHi-C data; extending the length of each enhancer to 2 kb surrounding its middle site, and extending the length of each promoter to 1 kb surrounding its middle site.
SEPT	One-hot encoding for 3 kb or 2kb DNA sequences surrounding middle site; Target: Promoter capture Hi-C (PCHi-C)	predicted score	Transfer learning	Train: the mixed data of labeled Enhancer-Promoter (EP) pairs of cell line #B and cell line #C are used as the source domain data, and the data of unlabeled EP pairs of cell line #A (50% random split) are used as the target domain data. Test: EP pairs of cell line #A (50% random split)	Developed for Enhancer-Promoter prediction. Sequences of enhancers are extended or cut to 3 kb flanking regions on location center of enhancers, and promoter are extended or cut to 2 kb fanking regions on location center of promoters.

Table S2

Chromosome		chr1	chr10	chr11	chr12	chr13	chr14	chr15	chr16	chr17	chr18	chr19	chr2	chr20	chr21	chr22	chr3	chr4	chr5	chr6	chr7	chr8	chr9	chrX
Sample negative-to-positive ratio	GM12878 CTCF	4.99	4.95	4.98	4.98	4.95	4.96	4.96	4.98	5.00	4.95	5.00	4.95	4.99	4.95	5.00	4.96	4.92	4.97	4.99	4.95	4.94	4.98	4.81
	GM12878 Polr2a	4.88	4.96	4.85	4.77	5.08	4.80	4.87	4.68	4.72	5.32	4.40	4.93	4.83	4.97	4.79	4.96	5.10	4.94	4.90	4.95	4.96	4.95	4.86
	HeLaS3 CTCF	4.97	4.98	4.83	4.99	4.98	5.00	4.98	4.97	4.99	4.99	4.96	4.99	5.00	5.00	5.00	4.98	4.98	4.96	4.99	4.97	4.97	4.99	4.97
	K562 Polr2a	4.80	4.99	4.83	4.93	5.51	4.99	4.92	4.83	4.75	5.24	4.49	5.05	4.89	5.02	4.79	4.96	5.05	4.94	4.80	4.95	4.97	4.78	4.89
	MCF7 Polr2a	4.92	4.98	4.96	4.98	5.00	4.94	4.97	4.98	4.69	5.00	4.97	4.94	4.49	5.00	5.00	4.97	4.98	4.98	4.96	4.95	4.97	4.97	5.00

Table S3

GM12878	K562	Helas3
AwgTfbsHaibGm12878Cebpbsc150V0422111UniPk	AwgTfbsHaibK562Cebpbsc150V0422111UniPk	AwgTfbsSydhHelas3CebpbIggrabUniPk
AwgTfbsSydhGm12878CfosUniPk	AwgTfbsSydhK562CfosUniPk	AwgTfbsSydhHelas3CfosUniPk
AwgTfbsSydhGm12878Chd2ab68301IggmusUniPk	AwgTfbsSydhK562Chd2ab68301IggrabUniPk	AwgTfbsSydhHelas3Chd2IggrabUniPk
AwgTfbsUtaGm12878CmycUniPk	AwgTfbsUtaK562CmycUniPk	AwgTfbsUtaHelas3CmycUniPk
AwgTfbsSydhGm12878Corestsc30189IggmusUniPk	AwgTfbsSydhK562Corestsc30189IggrabUniPk	AwgTfbsSydhHelas3Corestsc30189IggrabUniPk
AwgTfbsBroadGm12878CtcfUniPk	AwgTfbsBroadK562CtcfUniPk	AwgTfbsBroadHelas3CtcfUniPk
AwgTfbsSydhGm12878E2f4IggmusUniPk	AwgTfbsSydhK562E2f4UcdUniPk	AwgTfbsSydhHelas3E2f4UniPk
AwgTfbsSydhGm12878Elk112771IggmusUniPk	AwgTfbsSydhK562Elk112771IggrabUniPk	AwgTfbsSydhHelas3Elk112771IggrabUniPk
AwgTfbsBroadGm12878Ezh239875UniPk	AwgTfbsBroadK562Ezh239875UniPk	AwgTfbsBroadHelas3Ezh239875UniPk
AwgTfbsHaibGm12878GabpPcr2xUniPk	AwgTfbsHaibK562GabpV0416101UniPk	AwgTfbsHaibHelas3GabpPcr1xUniPk
BroadHistoneGm12878H3k27acStdAl	BroadHistoneK562H3k27acStdAl	BroadHistoneHelas3H3k27acStdAl
BroadHistoneGm12878H3k27me3StdAl	BroadHistoneK562H3k27me3StdAl	BroadHistoneHelas3H3k27me3StdAl
BroadHistoneGm12878H3k36me3StdAl	BroadHistoneK562H3k36me3StdAl	BroadHistoneHelas3H3k36me3StdAl
BroadHistoneGm12878H3k4me2StdAl	BroadHistoneK562H3k4me2StdAl	BroadHistoneHelas3H3k4me2StdAl
BroadHistoneGm12878H3k4me3StdAl	BroadHistoneK562H3k4me3StdAl	BroadHistoneHelas3H3k4me3StdAl
BroadHistoneGm12878H3k79me2StdAl	BroadHistoneK562H3k79me2StdAl	BroadHistoneHelas3H3k79me2StdAl
BroadHistoneGm12878H3k9acStdAl	BroadHistoneK562H3k9acStdAl	BroadHistoneHelas3H3k9acStdAl
BroadHistoneGm12878H4k20me1StdAl	BroadHistoneK562H4k20me1StdAl	BroadHistoneHelas3H4k20me1StdAl
AwgTfbsSydhGm12878JundUniPk	AwgTfbsSydhK562JundIggrabUniPk	AwgTfbsSydhHelas3JundIggrabUniPk
AwgTfbsSydhGm12878MaxIggmusUniPk	AwgTfbsSydhK562MaxIggrabUniPk	AwgTfbsSydhHelas3MaxIggrabUniPk
AwgTfbsSydhGm12878Mazab85725IggmusUniPk	AwgTfbsSydhK562Mazab85725IggrabUniPk	AwgTfbsSydhHelas3Mazab85725IggrabUniPk
AwgTfbsSydhGm12878Mxi1IggmusUniPk	AwgTfbsSydhK562Mxi1af4185IggrabUniPk	AwgTfbsSydhHelas3Mxi1af4185IggrabUniPk
AwgTfbsSydhGm12878NfyaIggmusUniPk	AwgTfbsSydhK562NfyaUniPk	AwgTfbsSydhHelas3NfyaIggrabUniPk
AwgTfbsSydhGm12878NfybIggmusUniPk	AwgTfbsSydhK562NfybUniPk	AwgTfbsSydhHelas3NfybIggrabUniPk
AwgTfbsSydhGm12878Nrf1IggmusUniPk	AwgTfbsSydhK562Nrf1IggrabUniPk	AwgTfbsSydhHelas3Nrf1IggmusUniPk
AwgTfbsHaibGm12878NrsfPcr1xUniPk	AwgTfbsHaibK562NrsfV0416102UniPk	AwgTfbsHaibHelas3NrsfPcr1xUniPk
AwgTfbsSydhGm12878P300IggmusUniPk	AwgTfbsSydhK562P300IggrabUniPk	AwgTfbsSydhHelas3P300sc584sc584IggrabUniPk
AwgTfbsSydhGm12878Pol2UniPk	AwgTfbsSydhK562Pol2UniPk	AwgTfbsSydhHelas3Pol2UniPk
AwgTfbsSydhGm12878Pol2s2IggmusUniPk	AwgTfbsSydhK562Pol2s2IggrabUniPk	AwgTfbsSydhHelas3Pol2s2IggrabUniPk
AwgTfbsSydhGm12878Rad21IggrabUniPk	AwgTfbsSydhK562Rad21UniPk	AwgTfbsSydhHelas3Rad21IggrabUniPk
AwgTfbsSydhGm12878Rfx5200401194IggmusUniPk	AwgTfbsSydhK562Rfx5IggrabUniPk	AwgTfbsSydhHelas3Rfx5200401194IggrabUniPk
AwgTfbsSydhGm12878Smc3ab9263IggmusUniPk	AwgTfbsSydhK562Smc3ab9263IggrabUniPk	AwgTfbsSydhHelas3Smc3ab9263IggrabUniPk
AwgTfbsHaibGm12878Taf1Pcr1xUniPk	AwgTfbsHaibK562Taf1V0416101UniPk	AwgTfbsHaibHelas3Taf1Pcr1xUniPk
AwgTfbsSydhGm12878TbpIggmusUniPk	AwgTfbsSydhK562TbpIggmusUniPk	AwgTfbsSydhHelas3TbpIggrabUniPk
AwgTfbsSydhGm12878Tr4UniPk	AwgTfbsSydhK562Tr4UcdUniPk	AwgTfbsSydhHelas3Tr4UniPk
AwgDnaseUwdukeGm12878UniPk	AwgDnaseUwdukeK562UniPk	AwgDnaseUwdukeHelas3UniPk
AwgTfbsSydhGm12878Usf2IggmusUniPk	AwgTfbsSydhK562Usf2IggrabUniPk	AwgTfbsSydhHelas3Usf2IggmusUniPk
AwgTfbsSydhGm12878Znf143166181apUniPk	AwgTfbsSydhK562Znf143IggrabUniPk	AwgTfbsSydhHelas3Znf143IggrabUniPk
AwgTfbsSydhGm12878Znf274UniPk	AwgTfbsSydhK562Znf274UcdUniPk	AwgTfbsSydhHelas3Znf274UcdUniPk

Table S4

	GM12878	HeLaS3		HMEC	HUVEC		IMR90	K562		KBM7		NHEK
	CTCF	CTCF	MYOG	CTCF	CTCF	HES7	CTCF	CTCF	MXI1	CTCF	RXRB	CTCF
	CTCFL	CTCFL	HEN1	CTCFL	CTCFL	SP4	CTCFL	CTCFL	MYC	CTCFL	NR1H2	CTCFL
	FOS	FOSL1	ITF2	JUNB	JUNB	KLF14	JUNB	FOS	HEY1	FOS	ERR3	JUNB
	JUNB	FOS	ESR2	FOS	FOS	TFE2	FOS	JUNB	HES7	JUNB	RARG	FOS
	ZN436	JUNB	HMX3	JUND	FOSL1	ITF2	JUND	FOSL1	BMAL1	JUN	RXRG	JUND
	JUN	JUN	MYF6	FOSL1	JUN	HEN1	ZN436	JUN	HES5	FOSL1	MAX	JUN
	JUND	JUND	ZBT18	JUN	JUND	MYOG	JUN	JUND	HES1	JUND	HES1	FOSL1
	FOSL1	FOSL2	KLF13	FOSL2	ZN436	MYF6	FOSB	ZN436	RXRG	ZN436	NR6A1	ZN436
	FOSL2	ZN436	MAX	FOSB	FOSL2	HES1	FOSL1	FOSL2	MAX	FOSL2	ZBT18	FOSB
	FOSB	FOSB	PTF1A	BACH2	FOSB	ZBT18	BACH2	FOSB	SP4	FOSB	SP4	FOSL2
	NFE2	NF2L2	RARA	BACH1	INSM1	MAX	NFE2	NF2L2	KLF14	NF2L2	CLOCK	NFE2
	BACH2	CR3L1	NR6A1	NF2L2	NFE2		MAFK	NFE2	PTF1A	NFE2	KLF14	BACH2
	NF2L2	NFE2	HMX2	NFE2	NF2L2		INSM1	BACH2	ESR2	BACH2	SP2	NF2L2
	MAFK	BACH2	LBX2	MAFK	BACH2		FOSL2	INSM1	CLOCK	INSM1	ESR2	INSM1
	BACH1	INSM1	HES1	INSM1	MAFK		BACH1	MAFK		MAFK	TYY1	MAFK
	INSM1	MAFK	KLF14	HES7	BACH1		NF2L2	BACH1		BACH1		BACH1
	TFEB	BACH1	SP2	HES5	CR3L1		HEY2	CR3L1		CR3L1		MAFG
	TFE3	TFEB	SP4	MYC	TFEB		MYCN	TFEB		TFEB		TFEB
	USF1	USF1	ZN331	HEY1	USF1		CR3L1	USF1		USF1		TFE3
	USF2	TFE3	BHA15	MITF	USF2		HES1	TFE3		USF2		USF2
	CR3L1	USF2	CLOCK	MAX	TFE3		MAFG	USF2		TFE3		USF1
	MAFG	ERR2	TYY1	BMAL1	MAFG		ZBTB4	MAFG		MAFG		CR3L1
	HEY2	NR4A2	ESR1	MYCN	KLF13		TFEB	MITF		MITF		HEY2
	MYCN	NR4A3	ASCL2	USF1	MITF		TFE3	KLF13		BATF		MYCN
motif	KLF13	THB	BHE40	TFE3	BATF		USF2	MYCN		KLF13		MITF
	MITF	ERR1	XBP1	BHE40	MYCN		USF1	HEY2		MYCN		BATF
	TFAP4	NR1H3	SP1	MXI1	ERR2			BATF		ERR2		MYC
	HTF4	MAFG	KLF9	CR3L1	NR4A2			TFAP4		NR4A2		MXI1
	MYOD1	MYCN	TFDP1	RFX3	THB			MYOD1		THB		MAX
	BATF	MITF	NR5A2	MLXPL	NR4A3			HTF4		NR1H3		HES1
	TFE2	BATF	TGIF2	USF2	NR1H3			ERR2		NR4A3		NR4A2
	ITF2	THA	ZIC3	SNAI2	ERR1			NR4A2		HEY2		THB
	SP4	NR1H2	COT2	HEY2	TFAP4			THB		ERR1		ERR2
	SP2	PPARA	PAX3	TFEB	MYOD1			NR1H3		MXI1		
	KLF14	RXRB	PAX7	MLX	HTF4			NR4A3		HEY1		
	MXI1	ERR3	HXA1	BATF	HEY2			ERR1		BMAL1		
	HES1	RXRG		SNAI1	PPARA			TFE2		MYC		
	MYC	RARG		CLOCK	THA			HEN1		HES5		
	HEY1	RXRA		HES1	RXRB			ITF2		HES7		
	BMAL1	HEY1		BHE41	RXRA			MYOG		TFAP4		
	HES5	HEY2		MAFG	ERR3			MYF6		MYOD1		
	HES7	HES5			NR1H2			THA		HTF4		
	MAX	HES7			RXRG			PPARA		TFE2		
	MYOG	BMAL1			NR6A1			RXRB		ITF2		
	HEN1	MYC			RARG			RXRA		HEN1		
	MYF6	MXI1			MXI1			ERR3		MYOG		
	ZBT18	TFAP4			HEY1			NR1H2		MYF6		
	CLOCK	MYOD1			BMAL1			NR6A1		PPARA		
	SP1	HTF4			MYC			RARG		THA		
	TFDP1	TFE2			HES5			ZBT18		RXRA		

Table S5

Motif	Number of samples with the motif detected	GM12878	HeLaS3	HMEC	HUVEC	IMR90	K562	KBM7	NHEK
CTCF	8	2.69E-06	1.08E-05	5.40E-08	4.41E-07	2.88E-08	8.27E-07	1.75E-06	7.02E-08
CTCFL	8	2.90E-06	1.08E-05	2.42E-05	1.53E-05	2.65E-05	9.93E-06	1.07E-05	1.46E-05
FOS	8	2.12E-04	1.30E-04	5.16E-04	1.60E-04	4.42E-04	1.80E-04	1.75E-04	1.24E-04
JUNB	8	2.12E-04	1.30E-04	5.16E-04	1.60E-04	4.42E-04	1.80E-04	1.75E-04	1.24E-04
JUN	8	3.38E-04	1.30E-04	6.09E-04	1.68E-04	1.85E-03	1.80E-04	1.86E-04	4.03E-04
JUND	8	3.38E-04	1.30E-04	5.81E-04	1.68E-04	1.61E-03	1.80E-04	1.86E-04	3.54E-04
FOSL1	8	3.85E-04	1.30E-04	6.09E-04	1.68E-04	2.19E-03	1.80E-04	1.86E-04	4.03E-04
FOSL2	8	7.08E-04	1.72E-04	8.17E-04	4.26E-04	3.85E-03	4.03E-04	3.62E-04	9.05E-04
FOSB	8	7.08E-04	5.56E-04	8.17E-04	5.47E-04	1.85E-03	7.32E-04	6.44E-04	8.09E-04
NFE2	8	1.11E-03	2.43E-03	1.83E-03	1.13E-03	2.22E-03	1.15E-03	1.27E-03	1.15E-03
BACH2	8	1.63E-03	2.43E-03	1.33E-03	1.13E-03	2.22E-03	1.20E-03	1.31E-03	1.15E-03
NF2L2	8	2.01E-03	1.07E-03	1.83E-03	1.13E-03	6.13E-03	1.08E-03	9.61E-04	1.58E-03
MAFK	8	2.68E-03	7.99E-03	1.83E-03	3.40E-03	2.52E-03	3.97E-03	4.30E-03	2.40E-03
BACH1	8	3.34E-03	8.70E-03	1.83E-03	4.59E-03	4.15E-03	5.35E-03	5.24E-03	4.11E-03
INSM1	8	4.16E-03	2.73E-03	8.78E-03	1.01E-03	3.24E-03	1.51E-03	1.69E-03	1.96E-03
TFEB	8	1.30E-02	1.26E-02	3.39E-02	9.49E-03	4.56E-02	9.72E-03	9.10E-03	1.54E-02
TFE3	8	1.30E-02	1.26E-02	1.22E-02	9.49E-03	4.56E-02	9.72E-03	9.10E-03	1.54E-02
USF1	8	1.30E-02	1.26E-02	1.22E-02	9.49E-03	4.97E-02	9.72E-03	9.10E-03	1.54E-02
USF2	8	1.30E-02	1.26E-02	3.39E-02	9.49E-03	4.97E-02	9.72E-03	9.10E-03	1.54E-02
CR3L1	8	1.55E-02	2.11E-03	1.72E-02	7.98E-03	4.29E-02	8.44E-03	6.61E-03	1.77E-02
MAFG	8	1.74E-02	1.91E-02	3.94E-02	1.44E-02	4.33E-02	1.27E-02	1.65E-02	1.34E-02
HEY2	8	2.57E-02	2.08E-02	3.39E-02	3.99E-02	4.29E-02	2.86E-02	3.04E-02	2.94E-02
MYCN	8	2.57E-02	1.99E-02	1.22E-02	3.48E-02	4.29E-02	2.86E-02	2.82E-02	2.94E-02
HES1	8	4.10E-02	3.67E-02	3.62E-02	4.64E-02	4.29E-02	3.89E-02	3.79E-02	4.86E-02
ZN436	7	3.14E-04	2.77E-04	-	3.85E-04	1.76E-03	2.51E-04	2.82E-04	5.38E-04
MITF	7	3.18E-02	1.99E-02	8.85E-03	2.59E-02	-	2.41E-02	2.48E-02	3.45E-02
BATF	7	3.85E-02	2.01E-02	3.43E-02	2.77E-02	-	2.88E-02	2.59E-02	3.86E-02
MXI1	7	4.10E-02	2.08E-02	1.72E-02	4.32E-02	-	3.89E-02	3.34E-02	4.84E-02
MYC	7	4.10E-02	2.08E-02	8.85E-03	4.32E-02	-	3.89E-02	3.34E-02	4.84E-02
MAX	7	4.10E-02	2.75E-02	1.21E-02	4.81E-02	-	4.22E-02	3.79E-02	4.84E-02
HEY1	6	4.10E-02	2.08E-02	8.85E-03	4.32E-02	-	3.89E-02	3.34E-02	-
BMAL1	6	4.10E-02	2.08E-02	1.21E-02	4.32E-02	-	3.89E-02	3.34E-02	-
HES5	6	4.10E-02	2.08E-02	8.85E-03	4.32E-02	-	3.89E-02	3.34E-02	-
HES7	6	4.10E-02	2.08E-02	8.85E-03	4.32E-02	-	3.89E-02	3.34E-02	-
KLF13	5	2.83E-02	2.74E-02	-	2.51E-02	-	2.74E-02	2.76E-02	-
TFAP4	5	3.36E-02	2.35E-02	-	3.92E-02	-	3.29E-02	3.49E-02	-
HTF4	5	3.36E-02	2.35E-02	-	3.92E-02	-	3.29E-02	3.49E-02	-
MYOD1	5	3.36E-02	2.35E-02	-	3.92E-02	-	3.29E-02	3.49E-02	-
TFE2	5	3.87E-02	2.36E-02	-	4.52E-02	-	3.45E-02	3.68E-02	-
ITF2	5	3.89E-02	2.36E-02	-	4.52E-02	-	3.45E-02	3.68E-02	-
SP4	5	3.91E-02	3.74E-02	-	4.46E-02	-	4.41E-02	4.64E-02	-
KLF14	5	3.91E-02	3.74E-02	-	4.46E-02	-	4.41E-02	4.70E-02	-
MYOG	5	4.46E-02	2.36E-02	-	4.52E-02	-	3.45E-02	3.68E-02	-
HEN1	5	4.46E-02	2.36E-02	-	4.52E-02	-	3.45E-02	3.68E-02	-
MYF6	5	4.46E-02	2.64E-02	-	4.52E-02	-	3.45E-02	3.68E-02	-
ZBT18	5	4.46E-02	2.64E-02	-	4.71E-02	-	3.64E-02	4.19E-02	-
CLOCK	5	4.62E-02	3.81E-02	3.53E-02	-	-	4.96E-02	4.65E-02	-
ERR2	5	-	1.55E-02	-	3.68E-02	-	3.30E-02	2.99E-02	4.89E-02
NR4A2	5	-	1.55E-02	-	3.68E-02	-	3.30E-02	2.99E-02	4.89E-02
THB	5	-	1.55E-02	-	3.68E-02	-	3.45E-02	2.99E-02	4.89E-02
NR4A3	4	-	1.55E-02	-	3.68E-02	-	3.45E-02	2.99E-02	-
ERR1	4	-	1.55E-02	-	3.68E-02	-	3.45E-02	3.18E-02	-
NR1H3	4	-	1.58E-02	-	3.68E-02	-	3.45E-02	2.99E-02	-
THA	4	-	2.02E-02	-	4.08E-02	-	3.62E-02	3.73E-02	-
NR1H2	4	-	2.02E-02	-	4.08E-02	-	3.62E-02	3.73E-02	-
PPARA	4	-	2.02E-02	-	4.08E-02	-	3.62E-02	3.73E-02	-
RXRB	4	-	2.02E-02	-	4.08E-02	-	3.62E-02	3.73E-02	-
ERR3	4	-	2.02E-02	-	4.08E-02	-	3.62E-02	3.73E-02	-
RXRG	4	-	2.02E-02	-	4.08E-02	-	4.15E-02	3.73E-02	-
RARG	4	-	2.02E-02	-	4.08E-02	-	3.62E-02	3.73E-02	-

Table S5 (continue)

RXRA	4	-	2.02E-02	-	4.08E-02	-	3.62E-02	3.73E-02	-
NR6A1	4	-	3.18E-02	-	4.08E-02	-	3.62E-02	4.10E-02	-
SP2	3	3.91E-02	3.74E-02	-	-	-	-	4.70E-02	-
ESR2	3	-	2.40E-02	-	-	-	4.68E-02	4.74E-02	-
SP1	2	4.64E-02	4.23E-02	-	-	-	-	-	-
TFDP1	2	4.64E-02	4.44E-02	-	-	-	-	-	-
PTF1A	2	-	3.09E-02	-	-	-	4.62E-02	-	-
TYY1	2	-	3.90E-02	-	-	-	-	4.93E-02	-
BHE40	2	-	4.03E-02	1.72E-02	-	-	-	-	-
HMX3	1	-	2.57E-02	-	-	-	-	-	-
RARA	1	-	3.18E-02	-	-	-	-	-	-
HMX2	1	-	3.58E-02	-	-	-	-	-	-
LBX2	1	-	3.58E-02	-	-	-	-	-	-
ZN331	1	-	3.80E-02	-	-	-	-	-	-
BHA15	1	-	3.80E-02	-	-	-	-	-	-
ESR1	1	-	3.92E-02	-	-	-	-	-	-
ASCL2	1	-	4.02E-02	-	-	-	-	-	-
XBP1	1	-	4.04E-02	-	-	-	-	-	-
KLF9	1	-	4.23E-02	-	-	-	-	-	-
NR5A2	1	-	4.49E-02	-	-	-	-	-	-
TGIF2	1	-	4.54E-02	-	-	-	-	-	-
ZIC3	1	-	4.54E-02	-	-	-	-	-	-
COT2	1	-	4.64E-02	-	-	-	-	-	-
PAX3	1	-	4.83E-02	-	-	-	-	-	-
PAX7	1	-	4.83E-02	-	-	-	-	-	-
HXA1	1	-	4.83E-02	-	-	-	-	-	-
RFX3	1	-	-	2.46E-02	-	-	-	-	-
MLXPL	1	-	-	2.87E-02	-	-	-	-	-
SNAI2	1	-	-	3.39E-02	-	-	-	-	-
MLX	1	-	-	3.39E-02	-	-	-	-	-
SNAI1	1	-	-	3.53E-02	-	-	-	-	-
BHE41	1	-	-	3.62E-02	-	-	-	-	-
ZBTB4	1	-	-	-	-	4.51E-02	-	-	-

Table S6

Sample	Percentage of similarity to *IGHV* reference sequence
AD102	98.61%
AD312	93.06%
AD324	97.22%
AD344	100.00%
AD401	64.91%
AD484	90.28%

Table S7

	Sample	102	312	324	344	401	484
Read alignment statistics	Total reads	948,029,258	952,568,936	962,597,696	928,396,764	921,586,154	943,118,124
	Normal paired	230,671,868	229,040,734	227,975,418	190,027,764	241,214,740	226,157,764
	Ligation motif present	535,901,842	479,621,330	493,454,930	515,211,736	480,234,368	509,891,762
	Alignable	858,223,558	867,092,356	883,427,000	844,824,800	832,474,774	854,272,762
Hi-C analysis statistics by Juicer	Unique reads	348,873,216	367,167,312	376,033,377	347,467,945	349,407,755	357,147,702
	Hi-C contacts	314,771,996	330,897,673	341,339,626	314,806,260	314,697,476	321,307,304
	Inter-chromosomal	72,179,180	85,636,523	85,782,728	62,185,458	59,258,481	64,967,149
	Intra-chromosomal	242,592,816	245,261,150	255,556,898	252,620,802	255,438,995	256,340,155
	Short range (<20kb)	110,379,302	92,854,062	110,974,633	101,510,722	112,777,329	120,382,809
	Long range(>20kb)	132,212,196	152,406,643	144,581,318	151,109,799	142,660,579	135,956,351
	# contact domains (10k)	1,339	1,133	1,591	1,649	1,796	1,583
	# loops identified	8,329	5,156	6,981	8,197	12,710	12,700

Table S8

ATAC-seq library	AHC4809	AHC4810	AHC4811	AHC4812	AHC4813	AHC4814
Sample alias	102	312	324	344	401	484
UNPAIRED_READS_EXAMINED	637,800	658,264	276,132	340,129	264,290	734,421
READ_PAIRS_EXAMINED	88,292,089	98,800,818	34,619,143	45,490,811	33,915,769	108,316,771
SECONDARY_OR_SUPPLEMENTARY_RDS	133,408	146,511	45,905	67,360	45,477	155,295
UNMAPPED_READS	1,534,238	1,779,714	552,602	711,579	544,162	1,943,299
UNPAIRED_READ_DUPLICATES	370,801	321,072	173,056	192,765	168,947	387,175
READ_PAIR_DUPLICATES	20,603,500	12,592,345	9,374,384	10,137,492	9,362,620	17,001,417
READ_PAIR_OPTICAL_DUPLICATES	52,705	48,141	17,300	32,559	14,974	46,095
PERCENT_DUPLICATION	0.234609	0.128648	0.2722	0.224128	0.277465	0.158211
ESTIMATED_LIBRARY_SIZE	158,714,350	355,042,962	51,831,896	86,457,182	49,559,803	308,568,921

Table S9

IGHV mutation test primers		
5’ IGHV leader primers	IGHV1L	AAATCGATACCACCATGGACTGGACCTGGAGG
	IGHV1bL	AAATCGATACCACCATGGACTGGACCTGGAG(C/A)
	IGHV2aL	AAATCGATACCACCATGGACACACTTTGCT(A/C)AC
	IGHV2bL	AAATCGATACCACCATGGACATACTTTGTTCCAC
	IGHV3aL	AAATCGATACCACCACCATGGAGTTTGGGCTGAGC
	IGHV3bL	AAATCGATACCACCACCATGGA(A/G)(C/T)T(G/T)(G/T)G(G/A)CT(G/C/ 5 T)(A/C/T)GC
	IGHV4L	AAATCGATACCACCATGAAACACCTGTGGTTCTT
	IGHV5L	AAATCGATACCACCATGGGGTCAACCGCCATC
	IGHV6L	AAATCGATACCACCATGTCTGTCTCCTTCCTC
3’ IGHJ primers	IGHJ1-2	TGAGGAGACGGTGACCAGGGTGCC
	IGHJ3	TGAAGAGACGGTGACCATTGTCCC
	IGHJ4-5	TGAGGAGACGGTGACCAGGGTTCC
	IGHJ6	TGAGGAGACGGTGACCGTGGTCCC

4C viewpoints and primers in MCF-7 cells			
	CAP2	ELOVL2	GREB1
Viewpoint	chr6:17390954-17391643	chr6:11047037-11047317	chr2:11691065-11691295
Outer Forward	GGGCTGATAACACAGTAGACTCA	GTGCTTTGCAAGGTTTAGATGACAT	AGAGGACTCGGGTAGAATGAGAA
Outer Reverse	GACAAAGGAGATGACGGGAGAAA	ACTTCCACATTACCATTTAGCCTGA	ATAGCTCAGGTCAATGTCCCAG
Nested Forward	GAATCTCAACAGAGGAGGTGACC	TTCTTAGAGGTCGGTTGTCACTT	TCAACTAGAATAAGTGCAGGGC
Nested Reverse	CACAGTTATCAGCCTCCTTGTCT	TACACATCAACCCATAAGGCCAG	TGGGTCAGTTGGCTTCATCT

	SIAH2	P2RY2
Viewpoint	chr3:150478089-150478323	chr11:72933550-72934990
Outer Forward	GCCTTTCTGTACATGTCAGTGGTT	CCTTGTGAACTCAGACTCAGATT
Outer Reverse	AGGTTGATTTCAGTAGCCTGTTTC	TGCTCCTCCTCAAATCTTATCAC
Nested Forward	GCTATTACTTTGCAAGCTGA	GGAGCCTGAGGAATTCACTTGAA
Nested Reverse	CCTGTTTCACGTTAGGAAAGAA	ATTGGAACACTAGACCTTGAGTAAG

Table S10

Cut-off value	0.1	0.15	0.18	0.2	0.22	0.25	0.3	0.4	0.5
GM12878_CTCF	0.4439	0.4798	0.4909	0.4956	0.5027	0.4998	0.4987	0.4715	0.4312
GM12878_Polr2a	0.3479	0.3865	0.4005	0.3990	0.3983	0.3954	0.3866	0.3407	0.2832

References

1. Tang, Z. *et al.* CTCF-Mediated Human 3D Genome Architecture Reveals Chromatin Topology for Transcription. *Cell* **163,** 1611–1627. ISSN: 0092-8674 (2015).

2. Li, G. *et al.* Extensive promoter-centered chromatin interactions provide a topological basis for transcription regulation. *Cell* **148,** 84–98. ISSN: 0092-8674 (2012).

3. Consortium, T. An integrated encyclopedia of DNA elements in the human genome. *Nature* **489,** 57. ISSN: 1476-4687 (2012).

4. Rao, S. S., Huntley, M. H., Durand, N. C. & Stamenova, E. K. A 3D Map of the Human Genome at Kilobase Resolution Reveals Principles of Chromatin Looping. *Cell* **159,** 1665–1680. ISSN: 0092-8674 (2014).

5. Chen, T. & Guestrin, C. *XGBoost: A Scalable Tree Boosting System* in *Proceedings of the 22Nd ACM SIGKDD International Conference on Knowledge Discovery and Data Mining* (ACM, San Francisco, California, USA, 2016), 785–794. ISBN: 978-1-4503-4232-2. doi:10.1145/2939672.2939785. http://doi.acm.org/10.1145/2939672.2939785.

6. Shrikumar, A., Greenside, P. & Kundaje, A. Reverse-complement parameter sharing improves deep learning models for genomics. *Biorxiv,* 103663 (2017).

7. Xu, B., Wang, N., Chen, T. & Li, M. Empirical evaluation of rectified activations in convolutional network. *arXiv preprint arXiv:1505.00853* (2015).

8. Alipanahi, B., Delong, A., Weirauch, M. T. & Frey, B. J. Predicting the sequence specificities of DNA- and RNA-binding proteins by deep learning. *Nat Biotechnol* **33,** 831–838. ISSN: 1087-0156 1546-1696 (2015).

9. Kulakovskiy, I. *et al.* HOCOMOCO: expansion and enhancement of the collection of transcription factor binding sites models. *Nucleic Acids Res* **44,** D116–D125. ISSN: 0305-1048 (2016).

10. Mathelier, A. *et al.* JASPAR 2014: an extensively expanded and updated open-access database of transcription factor binding profiles. *Nucleic Acids Research* **42,** D142–D147. ISSN: 0305-1048 (2014).

11. Gupta, S., Stamatoyannopoulos, J. A., Bailey, T. L. & Noble, W. Quantifying similarity between motifs. *Genome Biol* **8,** 1–9. ISSN: 1474-760X (2007).

12. Grant, C. E., Bailey, T. L. & Noble, W. FIMO: scanning for occurrences of a given motif. *Bioinformatics* **27,** 1017–1018. ISSN: 1367-4803 (2011).

13. Splinter, E., de Wit, E., van de Werken, H. J., Klous, P. & de Laat, W. Determining long-range chromatin interactions for selected genomic sites using 4C-seq technology: from fixation to computation. *Methods* **58,** 221–30. ISSN: 1046-2023 (2012).
14. Lassmann, T. TagDust2: a generic method to extract reads from sequencing data. *BMC Bioinformatics* **16,** 24. ISSN: 1471-2105 (2015).
15. Langmead, B. & Salzberg, S. L. Fast gapped-read alignment with Bowtie 2. *Nat. Methods* **9,** 357–9. ISSN: 1548-7091 (2012).
16. Thongjuea, S., Stadhouders, R., Grosveld, F. G., Soler, E. & Lenhard, B. r3Cseq: an R/Bioconductor package for the discovery of long-range genomic interactions from chromosome conformation capture and next-generation sequencing data. *Nucleic Acids Res.* **41,** e132. ISSN: 0305-1048 (2013).
17. Pedregosa, F. *et al.* Scikit-learn: Machine Learning in Python. *Journal of Machine Learning Research* **12,** 2825–2830 (2011).
18. Ritchie, M. E. *et al.* limma powers differential expression analyses for RNA-sequencing and microarray studies. *Nucleic Acids Res* **43,** e47–e47. ISSN: 0305-1048 (2015).
19. Love, M. I., Huber, W. & Anders, S. Moderated estimation of fold change and dispersion for RNA-seq data with DESeq2. *Genome Biol* **15,** 550. ISSN: 1474-760X (2014).
20. Bray, N. L., Pimentel, H., Melsted, P. & Pachter, L. Near-optimal probabilistic RNA-seq quantification. *Nature biotechnology* **34,** 525–527 (2016).
21. Li, H. Aligning sequence reads, clone sequences and assembly contigs with BWA-MEM. *arXiv preprint arXiv:1303.3997* (2013).
22. Picard toolkit. *Broad Institute, GitHub repository* (2019).
23. Li, H. *et al.* The sequence alignment/map format and SAMtools. *Bioinformatics* **25,** 2078–2079 (2009).
24. Zhang, Y. *et al.* Model-based analysis of ChIP-Seq (MACS). *Genome biology* **9,** 1–9 (2008).
25. Durand, N. C. *et al.* Juicer provides a one-click system for analyzing loop-resolution Hi-C experiments. *Cell systems* **3,** 95–98 (2016).
