# Supplementary material for: Chromatin interaction neural network (ChINN): a machine learning-based method for predicting chromatin interactions from DNA sequences
Source: Genome Biol. 2021 Aug 16;22:226. doi: 10.1186/s13059-021-02453-5 (PMC8365954; doi:10.1186/s13059-021-02453-5)
Supplement: Supplementary file 2 — Additional file 2. Review history. [file 13059_2021_2453_MOESM2_ESM.docx]

Review History

**First round of review**

**Reviewer 1**

**Were you able to assess all statistics in the manuscript, including the appropriateness of statistical tests used?**

There are no statistics in the manuscript.

**Were you able to directly test the methods?**

Yes.

**Comments to author:**

The manuscript entitled "Chromatin Interaction Neural Network (ChINN): A machine learning-based method for predicting chromatin interactions from DNA sequences" by Fan et al. introduces a model that predicts chromatin interactions between open chromatin regions by using only DNA sequences of the interacting open chromatin regions. The authors evaluated this model cross samples and applied it to a new clinical patient data, and claimed that ChINN can be applied to other cell lines or clinical samples given the knowledge of open chromatin regions, making it a useful tool to interrogate chromatin interactions when large-scale DNA sequencing is not applicable due to limited biological materials. The downstream analysis is interesting and important. However, there are several major flaws that limit the value of the presented analysis.

Major Comments

1. The authors demonstrate the performance of ChINN is comparable to functional genomic models, even worse in some cases within the same cell line. The authors should compare the performance of the functional genomic models when it is applied in other cell lines or clinical samples with ChINN. Particularly, in the first subsection of Results (Page 3), the prediction using only distance shows poor performance. This is reasonable, because the negative samples and the positive ones are distance-matched. But why genomic features along with distance feature can achieve significantly better performance than that using only genomic features?

2. I am confused about the distance-matched negative datasets with this statement "If the number of negative samples was less than 5 times of positive samples in a bin of a chromosome, more negative samples might be sampled from the same bin of another chromosome to make up the difference". What is the percentage of the number of negative samples was less than 5 times of positive samples in a bin in each cell line? Most of the chromatin interactions are within the same chromosome, I am confused why the authors choose the same bin of another chromosome? Is it the reason that the distance feature performs well even in the distance-matched negative datasets?

3. In the first subsection of Results, models trained on CTCF ChIA-PET data did not generalize well to each other in the cross-sample prediction. But in the next section, the model using sequences as features performs well in CTCF ChIA-PET cross-sample prediction. Usually, the genomic signals should be consistent whether derived from ChIP-seq experiments or sequences. Can the authors explain the difference of cross-sample performance in the two models?

4. Deep learning model is an end-to-end framework, why the authors split the training process of ChINN into 3 stages and feed the sequence features into the gradient boosted trees? There are many methods to obtain the feature importance scores in deep learning framework. In the second and third stage, the feature extractors trained in the first stage can be frozen and the fully-connected layer can be fine-tuned using the extend negative dataset. Please compare the machine-learning based model and fully deep learning based model.

5. "Thus, based on the F1 scores on the test sets, we selected the cutoffs that produced the best F1 scores for each dataset". The method for selecting F1 scores based on the test sets is not appropriate.

6. There have been many other methods designed to predict chromatin interactions using sequences as feature. For example, Li et al proposed a method called DeepTACT to predict 3D interactions, which learns features from the sequences of two genomic regions (Li et al, Nucleic acids research,2019). Fang et al proposed SEPT to predict the enhancer-promoter interactions in new cell line by using the cross-cell information (Fang et al, BMC Bioinformatics, 2020). The authors should discuss the state-of-art models in Introduction and compare with some of these models.

7. In page 4, ChINN discovered many motifs in its first layer. Most of the TFs are different in different cell lines. What is the relationship between the identified TFs and the corresponding cell lines? Are the identified TFs cell-type specific? Please discuss the shared TFs and diverse ones.

8. The sequence models including distance as a feature only slightly improved the performance. In Line 10 of Page 5, why does distance become the most important feature in all models? The positive samples and negative samples are distance-matched. Please explain how the distance feature contributes to the prediction.

9. In Line 12-20 of Page 5, the authors described their observations of importance scores of features, but do not have any discussion about these observations. It is confusing what point they want to put here.

10. What is the definition and calculation process of patient heterogeneity? How do the authors obtain the "Density" in Figure 5e-f? Every statistic should be clarified in the main text or clearly linked to the Supplementary.

11. Usually, the interaction predicted using cross-sample model should be more conserved and shows less cell-type specificity. But the authors studied the sample heterogeneity based the interactions predicted using cross-sample model. Please discuss why the cross-sample model can capture the heterogeneity of new samples.

Minor remarks:

1. The first subsection of Results entitled "Open chromatin interactions can be predicted from functional genomic features" might be moved to Supplementary. The results in this part do not contribute to the conclusion of this manuscript.

2. The subfigures of importance scores in Figure 3 is not informative. These figures should be moved to Supplementary or substituted with a more informative figure.

3. What is the definition of "from-DNAse models" in the legend of Figure 4? It seems that this term is not declared in the manuscript.

4. "uCLL" and "mCLL" were not defined in the main text. Please clarify the description of Figure 5d in the main text. In Figure 5b-c, please mark the sample ID of each donor. Why are the total true numbers different for the same sample?

5. All texts, figures, and tables in Supplementary should be properly cited in the main text.

6. All abbreviations should be declared at their first appearance. For example, "IFC" in line 29 of page 7 is not declared before using.

7. What is the definition for "extended datasets"? The "extended" was referred to the extension of sequence lengths or the augmentation of sample size? At least make a clear explanation in Supplementary.

**Reviewer 2**

**Were you able to assess all statistics in the manuscript, including the appropriateness of statistical tests used?**

Yes, and I have assessed the statistics in my report.

**Were you able to directly test the methods?**

Yes.

**Comments to author:**

Cao et al. develop a convolutional neural network, Chromatin Interaction Neural Network (ChINN) to predict open chromatin interactions from DNA sequences, regardless of distance restriction. ChINN can be used to predict CTCF-, RNA polymerase II- or HiC-associated interactions between open chromatin regions based on different training data. Applying ChINN to chronic lymphocytic leukemia (CLL) samples, authors find widespread heterogeneity of chromatin interaction in different subtypes of CLL. The article is in general well-written and easy to read. My main concern lies in the performance of ChINN. My suggestion is therefore to provide more convincing evidence of the good performance of ChINN.

Major comments:

-	According to the auPRC values provided by authors in the text, the performance of ChINN may be not convincing enough, especially the GM12878 and K562 HiC models applied to explore chromatin interactions in patient samples. Authors may want to explain why models can be used with low auPRC and F1 values, given that the predictions would be convincing only if the good performance of method is validated.

-	Why don't the authors compare the performances of ChINN with existing similar methods, such as 'Akita' mentioned in the text and 'DeepC' (Schwessinger et al. Nat Methods, 2020.). It may help to validate the performance of ChINN and show its advantage.

-	To motivate the application of ChINN by biological researchers, it may help to provide detailed user guide in which readers can clearly know how to apply ChINN in a new situation other than CLL samples. The URL of Github provided in the text is invalid, so it is not known whether ChINN is easy to use.

-	Authors prepare integrated Hi-C, ATAC-Seq and RNA-Seq libraries from 6 CLL samples, while the quality of these data are not described clearly enough, despite the description of Hi-C libraries summary of CLL samples presented in Supplementary Table 5.

Minor suggestions:

-	P2L1: DNA sequence may be one of the key words of this article.

-	P2L38: The authors may want to discuss 'DeepC' (Nat Methods. 2020 Nov;17(11):1118-1124.).

-	P2L47-50: The authors could test the ability of Akita to predict chromatin interaction using the data of CLL samples and compare the performance between two methods.

-	P3L26-27: Authors should define more clearly how large is the chromatin interaction anchor in general and provide related reference.

-	P3L46: The description of functional genomics is not clearly.

-	P3L48: A mathematical definition of auPRC will allow to understand what it is.

-	P4L1: The abbreviation of 'Pol2' may be ambiguous, it's also used to represent the subunit A of DNA polymerase epsilon catalytic. 'RNAPⅡ' or 'RNA PolⅡ' may be better.

-	P5L50-52: It would help to introduce how to define or recognize chromatin anchor more clearly.

-	P6L20-24: 'The GM12878 and K562 HiC model ... were acceptable when compared to the random auPRC values.', why? The performance of model should be acceptable when it achieves expected accuracy or function other than performs better than random prediction.

-	P7L9: The definition of IFC score is not clearly.

-	P8L2: 'RNA Polymerase II' should use abbreviation.

-	There are a few misspellings here and there, but the text is overall easy to follow. P11L3: The word 'analyses' is misspelled.

-	Authors may want to discuss the potential biological meaning of heterogeneity in chromatin interaction in CLL patients and how this findings will boost future study on cancer or 3D genome.

-	The figures should be checked more carefully. Figure 1 and 7 are not clear enough. In figure 4b, the numbers are overlapped with the bars. In figure 8e, the p values are overlapped with the lines.

-	Please confirm the format requirement of the reference. The style of reference in supplementary material is not consistent with the main text. Besides, reference 26 in the main text may be in the wrong format.

**Authors Response**

**Point-by-point responses to the reviewers’ comments:**

Reviewer #1:

Major Comments

1. The authors demonstrate the performance of ChINN is comparable to functional genomic models, even worse in some cases within the same cell line. The authors should compare the performance of the functional genomic models when it is applied in other cell lines or clinical samples with ChINN. Particularly, in the first subsection of Results (Page 3), the prediction using only distance shows poor performance. This is reasonable, because the negative samples and the positive ones are distance-matched. But why genomic features along with distance feature can achieve significantly better performance than that using only genomic features?

*Reply: Thanks for your comment. We did not compare the performance of the functional genomic models when it is applied in other cell lines or clinical samples with ChINN because functional genomics data is very resource intensive to obtain and for a lot of clinical samples we do not have all these functional genomics datasets available. This is the reason why, once we determined that prediction of chromatin interactions was possible through functional genomics methods, we proceeded to investigate whether it is possible to predict chromatin interactions from the underlying DNA sequence in combination with open chromatin information (the ChINN method).*

*The reason why genomic features along with distance feature can achieve significantly better performance than that using only genomic features can be explained from the Gradient boosted trees (GB) model that we used. The core of GB is decision tree. In a decision tree, the features would be selected as nodes to split the dataset. When using distance feature only, it is certainly impossible to split the dataset to positive or negative as there is only one split and the data are similar. But when using distance feature with other features together, the original datasets may already been split to small subsets by other features before using distance feature. In those small subsets split according to certain rules, distance feature may play some roles to split them to even smaller subsets. Besides, by adding one more feature, the pruning of the tree could also change, which may lead to different prediction results compared to without adding the feature.*

*We implemented the above explanation to Supplementary Text I and revised the sentence in the original manuscript as “These results showed that although distance alone cannot predict chromatin interactions, combining distance feature with other features together can help to distinguish the positive and negative chromatin interaction considering the working mechanisms of the GB model (Supplementary Text I)” to make it clear.*

2. I am confused about the distance-matched negative datasets with this statement "If the number of negative samples was less than 5 times of positive samples in a bin of a chromosome, more negative samples might be sampled from the same bin of another chromosome to make up the difference". What is the percentage of the number of negative samples was less than 5 times of positive samples in a bin in each cell line? Most of the chromatin interactions are within the same chromosome, I am confused why the authors choose the same bin of another chromosome? Is it the reason that the distance feature performs well even in the distance-matched negative datasets?

*Reply: Thanks for your comment. Here, we do not refer to “bin” as a segment of a chromosome; it is a segment of the distance distribution histogram. We computed the distance distribution of the positive samples in log scale and divided the distribution into 50 bins. For each positive sample, we try to generate 5 negative samples with distances similar to the positive sample (in the same bin of the distance distribution histogram) on the same chromosome.*

*But this is not always possible, and in such cases, we try to compensate by generating more negative samples of this particular distance bin on a different chromosome. The result is that the overall distance distributions of the positive samples and negative samples are matched. We did not include any inter-chromosome interactions in the negative samples.*

*To answer “What is the percentage of the number of negative samples was less than 5 times of positive samples in a bin in each cell line?”: The overall distance distribution for distance-matched negative samples can be found in Figure S1b. The “density” on the y-axis the frequency density (frequency divided by bin size). In total, out of 50 distance bins in each cell line, there are 4 bins in GM12878 Polr2a and 3 bins in K562 Polr2a datasets not reaching the 1:5 positive-to-negative ratio.*

*To answer “Is it the reason that the distance feature performs well even in the distance-matched negative datasets?”: We do not think the distance feature performs well in isolation, this is shown in Figure 1d for distance-matched negative datasets. Therefore, we disagree that the distance feature performs well in the distance-matched negative dataset. In addition, in Supplementary Figure 3c we showed that distance alone did not work well on the extended datasets. The extended datasets included more negative samples in additional to the distance-matched negatives. In this case, the distance distribution are not matched between positive and negative samples. If distance alone can work well, the performance of distance alone should be higher for these datasets.*

*To reduce the confusion, in the revised manuscript Supplementary Information: Methods: 1.1. Generation of positive and negative chromatin interactions, we added the sentence to clarify this as: “We note that here, “bin” does not refer to a segment of a chromosome; it is a segment of the distance distribution histogram”.*

3. In the first subsection of Results, models trained on CTCF ChIA-PET data did not generalize well to each other in the cross-sample prediction. But in the next section, the model using sequences as features performs well in CTCF ChIA-PET cross-sample prediction. Usually, the genomic signals should be consistent whether derived from ChIP-seq experiments or sequences. Can the authors explain the difference of cross-sample performance in the two models?

*Reply: Thanks for your comments. The difference of the performance of the cross-sample prediction using functional genomic data and sequence feature data may be explained by the different resolution of the data. ChIP-seq data can yield ChIP-seq peaks of over several hundred bp long (and they are further generalized into count data when preparing the input features), while the CTCF motif is only less than 20 bp (CTCF motif MA0139.1 from JASPAR database). In addition, the ChIP-seq peaks cannot tell the orientation of the CTCF binding, while sequence can tell the direction of CTCF motif. As CTCF orientation has been found to be important in chromatin interactions (PMID: 26276625), the sequence feature can give more information of the binding site as well as the binding orientation. Therefore, the information of CTCF in these two cross-sample prediction results is different. We added the above analysis to the Discussion section, as ” We also noticed that the models trained using sequence features of CTCF ChIA-PET data performs better than the models trained using functional genomics data from CTCF ChIA-PET in the cross-sample prediction. We reason that the difference of the performance may be explained by the different resolution of the data. ChIP-seq data can yield ChIP-seq peaks of over several hundred bp long (and they are further generalized into count data when preparing the input features), while the CTCF motif is only less than 20 bp (CTCF motif MA0139.1 from JASPAR database). In addition, the ChIP-seq peaks cannot tell the orientation of the CTCF binding, while sequence can tell the direction of CTCF motif. As CTCF orientation has been found to be important in chromatin interactions (PMID: 26276625), the sequence feature can give more information of the binding site as well as the binding orientation. Therefore, the information of CTCF in these two cross-sample prediction results is different.”.*

4. Deep learning model is an end-to-end framework, why the authors split the training process of ChINN into 3 stages and feed the sequence features into the gradient boosted trees? There are many methods to obtain the feature importance scores in deep learning framework. In the second and third stage, the feature extractors trained in the first stage can be frozen and the fully-connected layer can be fine-tuned using the extend negative dataset. Please compare the machine-learning based model and fully deep learning based model.

*Reply: We agree with what the reviewer said. The first stage deep learning method only works as a feature extractor to convert the raw sequence feature to numerical representation that can be used as input of the machine learning models. The stage 2 and 3 aim to train the different machine learning models to make the prediction. It is only a matter of which kind of model to use. We applied Deep Neural Network (NN) model as the reviewer suggested on both ChIA-PET and HiC extended dataset (second stage) using the same structure as that of stage one with all features. The PRC for NN models are plotted as below at left, while our original results using GB model are shown at right. As we can see, using GB model works better than using NN model by achieving higher AUPRC values. Hence we kept using the GB model on stage 2 and 3. The comparison was implemented in Supplementary Figure 3 and 4.*

*Moreover, in the revised manuscript, we said “The first stage deep learning method works as a feature extractor to convert the raw sequence feature to numerical representation that can be used as input of the machine learning models. The stage 2 and 3 aim to train the different machine learning models to make the prediction” in the paper in the Methods section as the second sentence of the Machine Learning of ChIA-PET data section, and “We also showed the results when training with NN model (same NN structure as ChINN sequence-based models) on the same datasets using sequence and distance feature to illustrate the superiority of GB model here (Supplementary Figure 3j).” and “The results trained with NN model using sequence and distance feature was also shown for reference (Supplementary Figure 4g).” in the end of the last two paragraphs of Results: Convergent CTCF motifs are important for prediction of CTCF-associated open chromatin interactions section.*

5. "Thus, based on the F1 scores on the test sets, we selected the cut-offs that produced the best F1 scores for each dataset". The method for selecting F1 scores based on the test sets is not appropriate.

*Reply: Thanks for pointing out this error. We are sorry for the mistake. We should use the validation datasets to select the cut-offs. We re-did the experiments, and we found that the cut-off threshold values remain the same as that when we using the test datasets. Hence we kept these values in the following experiments. In the revised supplementary methods, we changed the sentence to “Thus, based on the F1 scores on the validation sets, we selected the cut-offs that produced the best F1 scores for each dataset”.*

6. There have been many other methods designed to predict chromatin interactions using sequences as feature. For example, Li et al proposed a method called DeepTACT to predict 3D interactions, which learns features from the sequences of two genomic regions (Li et al, Nucleic acids research,2019). Fang et al proposed SEPT to predict the enhancer-promoter interactions in new cell line by using the cross-cell information (Fang et al, BMC Bioinformatics, 2020). The authors should discuss the state-of-art models in Introduction and compare with some of these models.

*Reply: Thanks for your comments. To discuss these previous methods, we included a detailed comparison between SEPT and DeepTACT in the introduction and Supplementary Table 1.*

*We implemented the evaluation of the SEPT on our datasets. For the SEPT method, following its pipeline, the sequences are extended or cut to 3 kb or 2 kb flanking regions from the centre. But the performance of SEPT on our dataset is extremely poor (AUPRC=0.0016 evaluated on K562 Hi-C test datasets with HeLaS3 as source data), as the sequences in our dataset are quite long, for example, the longest sequence is longer than 80 kb. Therefore, cutting the sequence to 3 kb or 2 kb according to what SEPT does would not let the model learn much useful information.*

*We could not directly compare our method with DeepTACT, because DeepTACT uses promoter captured Hi-C data of total B cells (tB), monocytes (Mon), foetal thymus (FoeT), total CD4+ T cells (tCD4), naive CD4+ T cells (nCD4), and total CD8+ T cells (tCD8), which is substantially different from the way that we perform our analysis (we use Hi-C and ChIA-PET data instead) and the type of chromatin interactions that we sought to investigate (we sought to capture open chromatin-associated chromatin interactions, whereas the chromatin interactions examined here are promoter-based chromatin interactions).*

*The detailed discussion for both SEPT and DeepTACT is shown in the 3rd paragraph of Introduction and the end of the Results section: Predicting chromatin interactions from open chromatin regions in our manuscript. Specifically, we say:*

*“We noted that there are other methods that predict Hi-C-like data and chromatin interactions, namely DeepTACT [24], SEPT [25], and DeepC [26]. A detailed comparison and description of these methods is discussed in Supplementary Table 1”. (Introduction)*

*“Other methods that predict Hi-C-like data and chromatin interactions are available, namely DeepTACT [24], SEPT [25], Akita [43], and DeepC [26]. However, except for SEPT, the other three machine learning methods are very different from ChINN in terms of the data. For example, DeepTACT uses promoter captured Hi-C input data, which is quite different from our use of Hi-C and ChIA-PET input data. For Akita and DeepC, their output consists of Hi-C contact matrices on lists of user-specified genomic regions, while our output is chromatin loops and probabilities of interaction across the whole genome. As a consequence, we cannott call loops from these partial Hi-C matrices of Akita and DeepC, because we would not know the background genomic interaction distribution. Therefore, direct comparison between ChINN output and Akita/DeepC output is not possible.*

*As for the SEPT, following its pipeline, we extended the input sequences or cut to 3 kb or 2 kb flanking regions from the centre. But SEPT performs worse as compared with ChINN on our dataset (AUPRC=0.0016 evaluated on K562 Hi-C test datasets with HeLaS3 as source data), as the sequences in our dataset are longer than these input sequences. Therefore, cutting the sequence to 3 kb or 2 kb according to what SEPT does would not let the model learn much useful information.” (Results)*

7. In page 4, ChINN discovered many motifs in its first layer. Most of the TFs are different in different cell lines. What is the relationship between the identified TFs and the corresponding cell lines? Are the identified TFs cell-type specific? Please discuss the shared TFs and diverse ones.

*Reply: Thanks for your suggestion. In our revised manuscript, we discuss detected motifs and cell lines in Section “Results Open chromatin interactions can be predicted from DNA sequences” as: 1) in the third paragraph, “Other than the CTCF motif, the remaining known transcription factor binding motifs learned by the data from different cell lines were different, indicating the possible cell-type-specific motifs. Our findings about the cell-type-specific motifs were supported by other evidences: studies show that cell-type-specific CTCF-mediated interactions are important in gene regulation [PMID: 28737770, PMID: 20133600] and CTCF-binding sites vary extensively across cell types [PMID: 19056695, PMID: 22955980]”, and 2) at the end of the last paragraph, “Some motifs, such as FOS, were learned by all models. But other motifs showed diversity, for example, ZN436 was detected by all other models except for HMEC, and ZIC3 was only detected by HeLaS3 (Supplementary Table 3). We noticed that the motifs detected in all cell lines exhibit smaller p-values than the cell-type-specific motifs, indicating that these “general” motifs are very important in predicting chromatin interactions. We speculate a model of chromatin interactions whereby there are general chromatin interactions facilitated by general transcription factors and common across different cell types, as well as cell-type specific chromatin interactions facilitated by cell-type specific transcription factors which can control cell-type specific transcription”.*

8. The sequence models including distance as a feature only slightly improved the performance. In Line 10 of Page 5, why does distance become the most important feature in all models? The positive samples and negative samples are distance-matched. Please explain how the distance feature contributes to the prediction.

*Reply: We thank the reviewer for his/her comments, and apologize that our original expression is not accurate. We should not say that distance feature is the most important feature, but say that combining distance feature with other features potentially contribute to the prediction when using GB classifier, as what we discussed in question 1. In the revised version, we updated our phrasing as “distance was the largest contributor (in terms of feature importance score) when it was used together with sequence features (Supplementary Figure 4a-d). But on its own, it was not very informative. This suggests its interaction with the sequence features are informative”.*

9. In Line 12-20 of Page 5, the authors described their observations of importance scores of features, but do not have any discussion about these observations. It is confusing what point they want to put here.

*Reply: We apologize for the confusion. In this section, we want to discuss the high feature correlation observed in the convergent anchor in CTCF ChIA-PET. As convergent CTCF motif has been observed in the anchor regions of CTCF ChIA-PET or Hi-C loops (PMID: 26276625; PMID: 26686651; PMID: 25497547), this suggests that the other sequence features or binding motifs at CTCF ChIA-PET anchors may also have such convergent orientation. Indeed, we observed a higher correlation between left anchor (reverse complement) and right anchor (forward) for CTCF ChIA-PET model. However, for Pol 2 model, there is no such convergent orientation. We have rewritten this section to add more discussion to this point as followed:*

*“Consequently, we focused on the sequence features that were important for the prediction. As convergent CTCF motif has been observed in the anchor regions of CTCF loops (PMID: 26276625; PMID: 26686651; PMID: 25497547), this suggests that the other sequence features or binding motifs at CTCF ChIA-PET anchors may also have such convergent orientation. Interestingly, in CTCF models the important sequence features were on different strands of the two anchors in a convergent manner (Figure 3a-b), while Pol2 models did not show such pattern (Figure 3c-e). For the CTCF models, importance scores of features on different strands of the two anchors showed good correlation, while importance scores of features on the same strand of the two anchors did not show much correlation (Figure 3f). In contrast, the importance scores of features of Pol2 models were generally highly correlated regardless of the strand. These results are consistent with the previously observed convergent CTCF motifs at CTCF ChIA-PET, and further suggest may be other transcription factors also binds to CTCF loops in a similar manner.”*

*Additionally, we think the reviewer in referring to the part where distance is the most important feature is referring to the paragraph above this: “We obtained the feature importance scores of the gradient boosted trees trained and validated using a set of extended datasets that includes more negative samples than the distance-matched datasets (Methods, Supplementary Figure 3a-d). Distance was the most important feature in all models.” We also note that the reviewer mentioned there was not much discussion. To address this issue, we have revised this section to discuss the observations more thoroughly and correct the confusion about the distance as follows: “We noted that the precision-recall curves of the datasets that used sequence features and distance (Supplementary Figure 3a) was better than that of sequence features alone (Supplementary Figure 3b). However, distance alone was uninformative by itself in predicting chromatin interactions (Supplementary Figure 3c), suggesting that it is the combination of distance as a property in addition with sequence features that provides predictive power.”*

10. What is the definition and calculation process of patient heterogeneity? How do the authors obtain the "Density" in Figure 5e-f? Every statistic should be clarified in the main text or clearly linked to the Supplementary.

*Reply: Thanks for your comments. The heterogeneity was used to indicate that the chromatin interactions are different across patients, which is, to the best of our knowledge, a novel biological finding as there have been very few studies of chromatin interactions that compared multiple patient samples and thus explored the diversity of chromatin interactions in different individuals. As shown in Figure 5e-f, the number of chromatin interactions in only 1 patient is high, indicating there was heterogeneity in patient samples.*

*As we did not have a formal quantification of “heterogeneity”, we have changed from density to proportion in the figures 5e-f to make our definitions clearer. To explain what is proportion, we mention that “y-axis: the proportion of total chromatin interactions that can be found in a particular number of samples” in the legend of Figure 5e-f. Moreover, to further explain our ideas, we revised the manuscript in the 5th paragraph in Section Results: Exploring chromatin interactions in patient samples as: “We use “heterogeneity” to indicate that the chromatin interactions are different across patients. We found extensive patient heterogeneity (Figure 5e-f), as observed from the lack of similarity of chromatin interactions across the new CLL samples and the overlapping peaks between new CLL samples and GM12878 Hi-C peaks. For example, Figure 5e indicates that 37% of Hi-C identified chromatin interactions can be found in only one sample, but not the other five samples. This indicates that many chromatin interactions can only be found in one sample, and is an illustration of the level of heterogeneity in terms of the presence and absence of chromatin interactions.”*

11. Usually, the interaction predicted using cross-sample model should be more conserved and shows less cell-type specificity. But the authors studied the sample heterogeneity based the interactions predicted using cross-sample model. Please discuss why the cross-sample model can capture the heterogeneity of new samples.

*Reply: We thank the reviewer for his/her question. We agree that the chromatin interactions predicted are likely to show less cell-type specificity, and the fact that sample heterogeneity can be seen in these predicted interactions, in spite of their lower likelihood of cell-type specificity, demonstrates that there is abundant sample heterogeneity present in 3D genome organization. We discussed this in the discussion as “Chromatin interactions predicted using cross-sample models are likely to show less cell-type specificity, and the fact that sample heterogeneity can be seen in these predicted chromatin interactions in spite of the lower likelihood of cell-type specificity due to the nature of the chromatin interaction prediction, suggests that chromatin interaction heterogeneity is widespread throughout the genome.”*

Minor remarks:

1. The first subsection of Results entitled "Open chromatin interactions can be predicted from functional genomic features" might be moved to Supplementary. The results in this part do not contribute to the conclusion of this manuscript.

*Reply: Thanks for your comments. When we first started to work on predicting chromatin interactions, there was some controversy in the field as to whether chromatin interactions could be predicted. While there were previous methods, we and others found that they had inflated performance measures (Xi and Beer, “Local epigenomic state cannot discriminate interacting and non-interacting enhancer-promoter pairs with high accuracy.” PLoS computational biology, 2018 and Cao and Fullwood, “Inflated performance measures in enhancer-promoter interaction-prediction methods, Nature Genetics, 2019). Therefore, we decided to investigate whether local epigenomic states could indeed predict chromatin interactions, and we showed that this was indeed possible in the first subsection of the results. In the original manuscript, and this has been kept in our revised manuscript, we say “In light of Xi et al. and our previous study showing that the existing prediction methods have exaggerated performances, we first tried to demonstrate that chromatin interactions could be predicted from functional genomic data.” In the first subsection of the results.*

*Next, motivated by our ability to predict chromatin interactions from functional genomic data, we proceeded to investigate whether it is possible to predict chromatin interactions from sequence data, in the rest of the manuscript. In our original manuscript, and this has been kept in our revised manuscript, we say “Motivated by the results above, we went on to explore whether open chromatin interactions can be predicted from DNA sequences.” In the second subsection of the results.*

*To address the reviewer’s concern that the results in the first subsection are not discussed as a conclusion of the manuscript, we revised the first part of the discussion as follows: “In this manuscript, we described two methods of predicting chromatin interactions, first, a functional genomics approach which uses local epigenomics data to accurately predict chromatin interactions, and second, a convolutional neural network, ChINN, which can extract sequence features and be coupled to classifiers to predict chromatin interactions between open chromatin regions using DNA sequences and distance. ChINN only requires the use of open chromatin data and showed good generalizability on the same type of chromatin interactions across different cell types.”*

*Also, we revised the overall conclusion as follows: “A functional genomics approach is able to predict chromatin interactions. A ChINN framework is able to predict chromatin interactions from open chromatin regions in the human genome, using DNA sequences and distances as features. This framework can be applied in other cell lines or clinical samples given the knowledge of open chromatin regions, making it a useful tool to interrogate chromatin interactions when large-scale functional genomics acquisition is not applicable due to limited biological materials.”*

2. The subfigures of importance scores in Figure 3 is not informative. These figures should be moved to Supplementary or substituted with a more informative figure.

*Reply: Thanks for your comments. We agree that the subfigures are not very informative and some should be moved to the Supplement. Therefore, in our revised manuscript, we kept the subfigures a, c, f, g, and o, which showed the feature importance of GM12878 CTCF, Pol II, and Hi-C model, and the Pearson correlations between feature importance scores of the two anchors, and move all the other subfigures to the Supplement.*

3. What is the definition of "from-DNAse models" in the legend of Figure 4? It seems that this term is not declared in the manuscript.

*Reply: Thanks for your comments. The “from-Dnase” model is the final model. The final model was built using pairs of open-chromatin regions. Pairs of open-chromatin regions with both regions overlapping with different anchors of a chromatin interaction are termed as positive, and the rest are negative samples. The final model is then trained using these data. To make this clearer and to indicate that “from DNAse” indicates the data is from open chromatin, we added “This final model was defined as the “from open chromatin” model” in the end of Section: methods - Machine learning of ChIA-PET data.*

*We revised the figure 4 legend as “Figure 4: Performances of the final “from-open chromatin” models and validations. a, Illustration of the two parameters, merging distance and extension size, used in constructing putative chromatin interactions anchors from open chromatin regions. b, Area under precision-recall curves of the “from-open ChIA-PET chromatin” models. c, Area under precision-recall curves of the HiC “from-open chromatin” models.”*

4. "uCLL" and "mCLL" were not defined in the main text. Please clarify the description of Figure 5d in the main text. In Figure 5b-c, please mark the sample ID of each donor. Why are the total true numbers different for the same sample?

*Reply: We apologise for the oversight. Previously we defined the “uCLL” and “mCLL” in Section “Results-Exploring chromatin interactions in a cohort of patient samples”. Now we moved the definition to section “Results-Exploring chromatin interactions inpatient samples” (after 4th paragraph) as “In addition, we also applied our ChINN framework on the six new CLL samples and built models using Hi-C and ATAC-seq data from each CLL sample. Clinical samples differ from each other due to a wide variety of factors including different driver mutations and different underlying genetics and epigenetics of each patient. Here we asked whether the subtype of the CLL samples could be one factor giving rise to patient heterogeneity. The CLL samples could be divided into two subtypes based on IGHV mutation status. In our data, two samples (102 and 344) are IGHV-unmutated CLL (uCLL) type and 4 samples (312, 324, 401, and 484) are IGHV-mutated CLL (mCLL) type. IGHV mutation status is an important prognostic biomarker in CLL, with mCLL being less aggressive (24)”.*

*Also, in Section “Results-Exploring chromatin interactions in a cohort of patient samples”, we deleted the original paragraph, and we included at the end of the first paragraph “Among 84 CLL samples, 34 of them are uCLL type and 50 of them are mCLL type.” The description of Figure 5d is clarified as “Figure 5d showed the predicted chromatin interactions in 6 new CLL samples and the differences of uCLL and mCLL samples. With the selected threshold...”*

*We also added the sample ID for Figure 5b-c. The total true numbers are same for the same sample as we checked (adding along the row in confusion matrix represent the total positive or negative number in each sample).*

5. All texts, figures, and tables in Supplementary should be properly cited in the main text.

*Reply: Thanks for your comments. We checked again to make sure that the texts, figures, and tables in Supplementary are properly cited in the main text.*

6. All abbreviations should be declared at their first appearance. For example, "IFC" in line 29 of page 7 is not declared before using.

*Reply: Thanks for your comments. IFC represents the fold change of the average number of chromatin interactions observed at the gene promoter in uCLL samples over that in mCLL samples, we have implemented it at the first appearance at the penultimate paragraph of Section: Exploring chromatin interactions in patient samples as “The results showed that although the trend of different IFC scores (the fold change of the average number of chromatin interactions observed at the gene promoter in uCLL samples over that in mCLL samples) could be observed”. We also checked other abbreviations to make sure they are declared at the first appearances.*

7. What is the definition for "extended datasets"? The "extended" was referred to the extension of sequence lengths or the augmentation of sample size? At least make a clear explanation in Supplementary.

*Reply: Thanks for your comments. Extended datasets are “distance-matched” datasets with more negative samples. They are not related to the extension size of the anchors. This is specified in the Supplementary Method section 1.1: “For each positive dataset, an extended negative dataset was generated by including pairs from Source 4, the corresponding distance-matched negative dataset, and the remaining negative samples from Source 1 and Source 2.” We made this sentence clearer in the Supplement by saying “For each positive dataset, an extended negative dataset was generated by including pairs from Source 4, the corresponding distance-matched negative dataset, and the remaining negative samples from Source 1 and Source 2. This dataset is called the “extended dataset” in the main text”.*

Reviewer #2:

Major comments:

1.According to the auPRC values provided by authors in the text, the performance of ChINN may be not convincing enough, especially the GM12878 and K562 HiC models applied to explore chromatin interactions in patient samples. Authors may want to explain why models can be used with low auPRC and F1 values, given that the predictions would be convincing only if the good performance of method is validated.

*Reply: Thanks for your comments. Besides comparison with Hi-C, we also validated the method performance through (1) the identification of motifs associated with chromatin interactions, such as CTCF, and (2) 4C validation of chromatin interactions. In the case of the apparent poor performance of ChINN in comparing Hi-C samples, we speculate that some of the chromatin interactions predicted by the ChINN method but which are not found by Hi-C might be real, but not captured by Hi-C because Hi-C tends to miss weaker chromatin interactions and is unable to capture every single chromatin interaction in a cell comprehensively. To test this, we used 4C in MCF-7 breast cancer cells to investigate chromatin interactions predicted by the 401 HiC model (Figure 6f-g), and showed that in MCF-7 4C could validate predicted chromatin interactions that were not detected by other chromatin interaction analysis methods.*

*Moreover, auPRC is a performance metric that is usually not very high, especially when the number of negative samples hugely overwhelms the positive samples. Because the number of chromatin interactions in the entire genome, relative to the number of genomic regions with no reported chromatin interactions by Hi-C, is not very high, therefore the number of negative samples in our data hugely overwhelm the positive samples. As such, it is expected that the auPRC score will not be very high when applying GM12878 and K562 HiC models to other cell lines or patient samples. This is reflected by the fact that the auPRC of random expectation is very, very low in such samples with such a low number of positive samples in relation to negative samples, and so we chose to compare our results with the auPRC of random expectation in order to demonstrate that our result perform significantly better than a random classifier.*

*Further, the ChINN method only takes as input the sequences of DNA at open chromatin regions of the genome for prediction. If more types of data are input into the model, the performance of the model is likely to improve, but at the cost of requiring more datatypes which are expensive and labor-intensive to acquire.*

*Nevertheless we agree that ChINN can be further improved in the figure for more accurate prediction of chromatin interaction methods.*

*To discuss our ideas in the text, we revised the results section as follows “We also generated pairs between anchors constructed from open chromatin regions in GM12878 and K562 Hi-C datasets with different combinations of merging distances and extension sizes (Supplementary Figure 6a). We kept to the same parameters as the CTCF model, i.e. merging size of 3,000 and extension size of 1,000, to train gradient boosted trees due to the insignificant difference in auROC achieved by different parameters. The GM12878 and K562 Hi-C model had relatively low auPRC in the within-sample and cross-sample evaluation (Figure 4c), but these auPRC values were still many times that of the random auPRC values. Moreover, some of the predicted chromatin interactions in MCF-7 cells using 4C-seq were able to be validated by our Hi-C models (Supplementary Figure 6b-d).”*

*We also revised the discussion as follows: “The application of ChINN models with gradient boosted tree classifiers to a set of CLL ATAC-seq samples showed that several of the predicted chromatin interactions could be validated by Hi-C. However, we note that the auPRC scores of ChINN reported, particularly the GM12878 and K562 models applied to explore chromatin interactions in patient samples, was around 0.26-0.6, which is consistent with cross-sample testing of other epigenomics machine learning prediction methods such as DeepHistone (Yin et al., BMC Genomics, 2019). However, these auPRC scores are not very high, which could be due to several reasons.*

*First, auPRC is a performance metric that is usually not very high, especially when the number of negative samples hugely overwhelm the positive samples. Because the number of chromatin interactions in the entire genome, relative to the number of genomic regions with no reported chromatin interactions by Hi-C, is not very high, therefore the number of negative samples in our data hugely overwhelm the positive samples. As such, it is expected that the auPRC score will not be very high when applying GM12878 and K562 HiC models to other cell lines or patient samples.*

*Second, the ChINN method only takes as input the sequences of DNA at open chromatin regions of the genome for prediction. If more types of data are input into the model, the performance of the model is likely to improve, but at the cost of requiring more datatypes which are expensive and labor-intensive to acquire.*

*Third, while there were also chromatin interactions that were predicted but not validated by Hi-C, our results showing that 4C could validate predicted chromatin interactions in MCF-7 cells that were not identified by Hi-C suggest that these so-called “false positives” might potentially be real chromatin interactions that were simply not captured by Hi-C due to limited sequencing depth of Hi-C libraries.*

*In future work, further development of Hi-C and other chromatin interaction sequencing methods to comprehensively capture chromatin interactions will allow for a better comparison with chromatin interaction predictions. Additionally, further development and refinement of ChINN to improve the accuracy of chromatin interaction prediction is warranted.”*

2. Why don't the authors compare the performances of ChINN with existing similar methods, such as 'Akita' mentioned in the text and 'DeepC' (Schwessinger et al. Nat Methods, 2020.). It may help to validate the performance of ChINN and show its advantage.

*Reply: Thanks for your comments. We have included a section to compare ChINN with Akita and DeepC in the introduction section as followed:” For Akita and DeepC, their output is Hi-C contact matrix on a list of user-specified genomic regions, while our output is chromatin loops and probabilities of interaction across the whole genome. As a consequence, we can’t call loops from these partial Hi-C matrix of Akita and DeepC, because we would not know the background genomic interaction distribution. Therefore, direct comparison between ChINN output and Akita/DeepC output is not possible.”*

3. To motivate the application of ChINN by biological researchers, it may help to provide detailed user guide in which readers can clearly know how to apply ChINN in a new situation other than CLL samples. The URL of Github provided in the text is invalid, so it is not known whether ChINN is easy to use.

*Reply: Thanks for your comments. We have made the codes of ChINN public and provided detailed user guide as a readme file in the github repository. The new link is: https://github.com/mjflab/chinn.*

4. Authors prepare integrated Hi-C, ATAC-Seq and RNA-Seq libraries from 6 CLL samples, while the quality of these data are not described clearly enough, despite the description of Hi-C libraries summary of CLL samples presented in Supplementary Table 5.

*Reply: Thanks for your comments. We have added detailed information of ATAC-seq sequencing libraries in Supplementary Table 7. For RNA-seq, we used kallisto to quantify transcript abundance and therefore no mapping was done. We have added the per base quality figures of each RNA-seq library in Supplementary Figure 11.*

Minor suggestions:

1. P2L1: DNA sequence may be one of the key words of this article.

*Reply: Thank you for the suggestion. We have made it one of the key words.*

2. P2L38: The authors may want to discuss 'DeepC' (Nat Methods. 2020 Nov;17(11):1118-1124.).

*Reply: Thank you for your comments. We have included a section to compare ChINN with DeepC in the introduction section as follows: “For Akita and DeepC, their output consists of Hi-C contact matrices on lists of user-specified genomic regions, while our output is chromatin loops and probabilities of interaction across the genome. As a consequence, we cannot call loops from these partial Hi-C matrices of Akita and DeepC because we would not know the background genomic interaction distribution. Therefore, direct comparison between ChINN output and Akita/DeepC output is not possible.”*

3. P2L47-50: The authors could test the ability of Akita to predict chromatin interaction using the data of CLL samples and compare the performance between two methods.

*Reply: Thank you for your comments. We have included a section to compare ChINN with Akita in the introduction section as follows: “For Akita and DeepC, their output consists of Hi-C contact matrices on lists of user-specified genomic regions, while our output is chromatin loops and probabilities of interaction across the genome. As a consequence, we cannot call loops from these partial Hi-C matrices of Akita and DeepC, because we would not know the background genomic interaction distribution. Therefore, direct comparison between ChINN output and Akita/DeepC output is not possible.”*

4. P3L26-27: Authors should define more clearly how large is the chromatin interaction anchor in general and provide related reference.

*Reply: Thank you for your comments. Hi-C anchors are normally 5kb to 100kb long in most publications, and in rare extremely deeply sequenced samples, the resolution is down to 1kb size (PMID: 30570701; PMID: 29685368). ChIA-PET is normally several kb long (PMID: 20181287; PMID: 31336684). We have modified the text as follows: “The enhancers used were typically hundreds of base pairs, while the chromatin interaction anchors were much larger in size. For example, Hi-C anchors is normally 5kb to 100kb long (while only in rare case with extremely deep sequencing are the anchors down to 1kb size) (PMID: 30570701; PMID: 29685368), and ChIA-PET is normally several kb long (PMID: 20181287; PMID: 31336684)”.*

5. P3L46: The description of functional genomics is not clearly.

*Reply: Thank you for your comments. Here functional genomics means the use of ChIP-based sequencing data for feature extraction. To make this part clear, we changed “Many of these methods rely on using various functional genomics data, including chromatin immunoprecipitation sequencing (ChIP-seq) data of transcription factors and histone modifications, open chromatin data, and transcription data” to say “Many of these methods rely on using various functional genomics data, meaning the use of chromatin immunoprecipitation sequencing (ChIP-seq) data of transcription factors and histone modifications, open chromatin data, and transcription data” in order to provide the definition for functional genomics in Introduction section.*

6. P3L48: A mathematical definition of auPRC will allow to understand what it is.

*Reply: Thank you for your comments. The auPRC is calculated as the area under the PR curve. A PR curve shows the trade-off between precision and recall across different decision thresholds. We implemented the above description of auPRC in the third paragraph of Section “Results- Open chromatin interactions can be predicted from functional genomic features” where it first shown as “A PR curve shows the trade-off between precision and recall across different decision thresholds. The auPRC is calculated as the area under the PR curve”.*

*7. P4L1: The abbreviation of 'Pol2' may be ambiguous, it's also used to represent the subunit A of DNA polymerase epsilon catalytic. 'RNAPⅡ' or 'RNA PolⅡ' may be better.*

*Reply: Thank you for your suggestion. We have changed all the expression of ‘Pol2’ to ‘RNA PolⅡ’ in the manuscript and Supplementary materials. But we did not change in the figures, because the expression is too long to be plotted. Rather, we added the sentence: “The ‘Pol2’ in the figure represents ‘RNA Pol II’” in the figure legends to make this clear.*

8. P5L50-52: It would help to introduce how to define or recognize chromatin anchor more clearly.

*Reply: Thank you for your comments. Anchors refer to the genomic regions that are interacting with each other as shown in Figure 1a. Here we want to construct a list of paired open chromatin genomic regions (anchors) to be used as the input of our model. We have modified the text as follows: “To be able to predict chromatin interactions between open chromatin regions, the models need to be able to predict chromatin interactions between paired genomic regions (anchors) of open chromatin regions.”*

9. P6L20-24: 'The GM12878 and K562 HiC model ... were acceptable when compared to the random auPRC values.', why? The performance of model should be acceptable when it achieves expected accuracy or function other than performs better than random prediction.

*Reply: Thank you for your comments. We have changed this sentence to say “… were higher when compared to the random auPRC values” to be clearer. As there are no prior chromatin interaction methods that investigate the same chromatin interactions as us (as discussed in the introduction, the other four methods investigate different types of chromatin interactions), there is no prior expectation of accuracy, therefore we changed the word “acceptable” to “higher” to reflect this.*

10. P7L9: The definition of IFC score is not clearly.

*Reply: Thank you for your comments. IFC represents the fold change of the average number of chromatin interactions observed at the gene promoter in uCLL samples over that in mCLL samples, we have implemented this at its first appearance at the 9th paragraph of Section Results - Exploring chromatin interactions in patient samples as “The results showed that although the trend of different IFC scores (the fold change of the average number of chromatin interactions observed at the gene promoter in uCLL samples over that in mCLL samples) could be observed”.*

11. P8L2: 'RNA Polymerase II' should use abbreviation.

*Reply: Thank you for your suggestion. We revised by defining the abbreviation of 'RNA Polymerase II’ in Introduction when at its first appearance as “RNA Polymerase II (RNA Pol II)”, and kept using its abbreviation in the following sections.*

12. There are a few misspellings here and there, but the text is overall easy to follow. P11L3: The word 'analyses' is misspelled.

*Reply: We are sorry for the mistakes. We went through the manuscript and corrected the misspellings that we found.*

13. Authors may want to discuss the potential biological meaning of heterogeneity in chromatin interaction in CLL patients and how this findings will boost future study on cancer or 3D genome.

*Reply: Thank you for your comments. We have refined the discussion to be more detailed about the biological significance. The revised discussion is follows:*

*“Application of ChINN models in CLL revealed that although there were chromatin interactions that were ubiquitous in all samples, there were a large number of patient-specific chromatin interactions and also chromatin interactions that were found in fewer than half the samples. Chromatin interactions predicted using cross-sample models are likely to show less cell-type specificity, and the fact that sample heterogeneity can be seen in these predicted chromatin interactions in spite of the lower likelihood of cell-type specificity due to the nature of the chromatin interaction prediction, suggests that chromatin interaction heterogeneity is widespread throughout the genome. Moreover, the observation of predicted chromatin interaction heterogeneity agrees with our observations that there exist both ubiquitous chromatin interactions and patient-specific chromatin interactions in the 6 Hi-C libraries from the 6 CLL patient samples that we examined. While we previously observed patient-specific chromatin interactions at particular loci, here we show that this phenomenon is widespread [50]. To the best of our knowledge, this observation is novel and has not been previously reported in the 3D genome organization field.*

*One potential reason for these different chromatin interactions could be due to different patient subtypes. Importantly, we found systematic differences in chromatin interactions involving important CLL prognostic genes, such as LPL and CD38, between the IGHV-mutated and IGHV-unmutated subtypes. These results suggest that differences in chromatin interaction landscapes between CLL subtypes could have important functional implications in CLL biology. Moreover, differences in chromatin interaction presence or absence may lead to different expression of oncogenes in cancers.*

*Our observation of widespread patient heterogeneity in patient cancer samples highlights the need for precision medicine and the need to understand chromatin interactions in individual patient samples. Machine learning offers one way for us to predict chromatin interactions in a cost-effective manner. The ChINN method may be useful in the future in understanding chromatin interactions in large cohorts of clinical samples and identifying chromatin interaction-based biomarkers that can be used to distinguish between different subtypes of cancer which may help in the development of precise therapies for the different subtypes of cancer.”*

14. The figures should be checked more carefully. Figure 1 and 7 are not clear enough. In figure 4b, the numbers are overlapped with the bars. In figure 8e, the p values are overlapped with the lines.

*Reply: Thank you for your comments. We have rectified the font issues in Figure 1 and 7, and the overlapped issue in Figure 4b and Figure 8e.*

15. Please confirm the format requirement of the reference. The style of reference in supplementary material is not consistent with the main text. Besides, reference 26 in the main text may be in the wrong format.

*Reply: Thank you for your comments. We have corrected the format of original reference 26. And we changed the reference style in main text as “Numbered” to be consistent with that of the supplementary material.*

**Second round of review**

**Reviewer 1**

The authors have replied the questions raised by the reviewer. But some the comments are not been well answered. The authors evaluated ChINN cross samples and applied it to a new clinical patient data. Then they study the sample heterogeneity based the interactions predicted using cross-sample model which are more likely to capture conserved and less cell-type specific information. I doubt the heterogeneity between samples captured in this way. Besides, there have been many methods developed to predict 3D chromatin interactions using sequences since five years ago, but the authors do not compare with the state-of-art methods and do not show enough bright spot of this work.

1. The strategy the authors used to generate the negative samples are not reasonable. Distance-matched negative samples should be generated from all possible combination of anchor pairs, and simultaneously satisfied the same distance distribution as positive pairs. Please check the strategy used by TargetFinder (Whalen et al, Nature Genetics, 2016). And the author didn’t answer the question that what proportion of negative samples are generated on a different chromosome, which should be the number of negative samples derived on a different chromosome divide the total number of negative samples.

2. I didn’t catch the necessity between the first two sections, i.e. the “functional genomic model” and the sequence model (ChINN). If the author want the “functional genomic model” to work as a baseline model, they may just put it in the second section and compare the performance of these two models to show the advantage of ChINN.

3. The model trained using sequence features of CTCF ChIA-PET data performs better than the models trained using functional genomics data from CTCF ChIA-PET in the cross-sample prediction. The author claim that this is because the low resolution of ChIP-seq data and that the ChIP-seq peaks cannot tell the orientation of the CTCF binding, while sequence can tell the direction of CTCF motif. If this is the reason, why the model trained using sequence features of Pol2 ChIA-PET data performs worse than the models trained using functional genomics data?

4. The authors said they have corrected the way of selecting the cut-offs for F1 scores. Please add the corresponding experiments and the results. Even the threshold may remain the same, the authors should use numbers and results to show it in the response letter.

5. There have been many methods developed to predict 3D chromatin interactions using sequences since five years ago, the authors did not show enough bright spot of this work. I still think the authors should find a way to compare ChINN with at least three of the state-of-art methods to show the advantage of ChINN. Otherwise, when researchers need to predict chromatin interactions, they can just choose other existing methods rather than ChINN.

6. Usually, the interaction predicted using cross-sample model should be more conserved and shows less cell-type specificity. Then, how can the authors study the sample heterogeneity based the interactions predicted using cross-sample model? The discussion now did not answer this question.

**Reviewer 2**

The authors addressed most of my previous concerns. I suggest to accept this article after authors have addressed my minor concerns.

1.Previous major concern 3: The new link (https://github.com/mjflab/chinn.) is still not found.

2.The legend of Supplementary Table 7, "ATAC-C libraries summary of CLL samples": Does 'ATAC-C' mean 'ATAC-seq' ?

**Authors Response**

**Point-by-point responses to the reviewers’ comments:**

Reviewer #1:

The authors have replied the questions raised by the reviewer. But some the comments are not been well answered. The authors evaluated ChINN cross samples and applied it to a new clinical patient data. Then they study the sample heterogeneity based the interactions predicted using cross-sample model which are more likely to capture conserved and less cell-type specific information. I doubt the heterogeneity between samples captured in this way. Besides, there have been many methods developed to predict 3D chromatin interactions using sequences since five years ago, but the authors do not compare with the state-of-art methods and do not show enough bright spot of this work.

*Response 4. The reviewer makes three main criticisms, which we will address point-by-point here.*

A. “Then they study the sample heterogeneity based the interactions predicted using cross-sample model which are more likely to capture conserved and less cell-type specific information. I doubt the heterogeneity between samples captured in this way.”

*Response 4A. We agree that predicting chromatin interactions using cross-sample models from very different cell lines will be more likely to capture conserved and less cell-type specific information. We mentioned this point in our discussion section, “We note that chromatin interactions predicted using cross-sample models are likely to show less cell-type specificity, and the fact that sample heterogeneity can be seen in these predicted open chromatin interactions in spite of the lower likelihood of cell-type specificity due to the nature of the chromatin interaction prediction, suggests that chromatin interaction heterogeneity is widespread throughout the genome.”*

*However, it is still possible to mitigate this problem of capturing mainly conserved and less cell-type specific information. As a mitigation measure, we used cell lines that come from the same lineage as that of the clinical samples. In the section on “Exploring chromatin interactions in patient samples”, in the revised manuscript, we included the sentences: “We used GM12878 and K562 Hi-C models for this prediction because CLL, GM12878 and K562 all come from haematopoietic lineages, and therefore GM12878 and K562 predictions would be likely to have captured both general chromatin interaction mechanisms and tissue-specific mechanisms that are relevant to haematopoietic cells”.*

*Additionally, to further avoid the issue of predicting using cross-sample models from very different cell lines, in the later part of the “Exploring chromatin interactions in patient samples” section, we also applied our ChINN framework on the six new CLL samples and built models using this. We reorganized the later part of the “Exploring chromatin interactions in patient samples” subsection for greater clarity, and say “we applied our ChINN framework on the six new CLL samples and built models using Hi-C and ATAC-seq data from each CLL sample. Models built using CLL samples would have captured general chromatin interaction mechanisms, tissue-specific mechanisms relevant to haematopoietic cells, as well as CLL-specific mechanisms”.*

*The major source of heterogeneity between different patient samples observed comes from different locations of open chromatin in the patient samples. To make this explanation clear in the revised manuscript, we included two paragraphs in “Exploring chromatin interactions in patient samples, as follows:*

*“Genomic sequences are almost identical across different patient samples, except for regions of patient-specific cancer structural variations and single nucleotide variations. Thus, if two anchors are identical in a different cell type, the probability that they are interacting given by the model will be the same. In this study, we have limited the scope of chromatin interaction prediction to only open chromatin regions. and call the predicted chromatin interactions “open chromatin interactions”.*

*Open chromatin profiles have been used to cluster cell types and cancer subtypes (Rendeiro et al, Nature Commun, 2016). With the assumption that the mechanisms of chromatin interactions are similar between different patient samples with the same cancer, and with the patient-specific open chromatin regions, we explored the different chromatin interactions arising due to open chromatin differences between patient samples.”*

*One point which we feel demonstrates the validity of the heterogeneity of predicted open chromatin associated chromatin interactions observed is the fact that actual chromatin interactions called from Hi-C data from six CLL samples also displays heterogeneity. To make this clear, we revised the paragraph discussing Figure 6b as follows “Our Hi-C libraries identified 1795 open chromatin interactions unique to uCLL samples and 10663 open chromatin interactions unique to mCLL samples (Figure 6a). Moreover, uniqueness analysis of the Hi-C interactions from these six CLL samples similarly showed high patient heterogeneity (Figure 6b). Thus, both predicted open chromatin interactions and Hi-C identified interactions indicate high patient heterogeneity.”*

*Finally, to make it clear that we are discussing heterogeneity in the context of open chromatin associated chromatin interactions, we have revised the rest of the manuscript discussing heterogeneity to say “open chromatin interactions”.*

B. “Besides, there have been many methods developed to predict 3D chromatin interactions using sequences since five years ago, but the authors do not compare with the state-of-art methods ...”

*Response 4B. First, several of the methods developed to predict 3D chromatin interactions using sequences five years ago, such as TargetFinder, had exaggerated performances because the training and test datasets overlapped with each other. We discussed this issue in a correspondence with Nature Genetics (Cao et al, Nature Genetics, 2019) regarding TargetFinder and other enhancer-promoter prediction methods.*

*Besides our lab, the lab of Michael Beer also made similar observations in a perspective (Xi and Beer, PLoS Computational Biology, 2018). As they say in their paper “We report an experimental design issue in recent machine learning formulations of the enhancer-promoter interaction problem arising from the fact that many enhancer-promoter pairs share features. Cross-fold validation schemes which do not correctly separate these feature sharing enhancer-promoter pairs into one test set report high accuracy, which is actually arising from high training set accuracy and a failure to properly evaluate generalization performance.”*

*Thus, the previous methods, such as TargetFinder, are unreliable and should not be taken as methods that work successfully to predict 3D chromatin interactions using sequences. To make this clear, we revised our introduction, as follows: “Methods such as RIPPLE [17], TargetFinder [19], and JEME [15] reported high performances in predicting enhancer-promoter interactions using supervised machine learning approaches. However, the reported performances were exaggerated by using cross-validation with random splitting of samples [22] [23]. The lack of effective machine learning approaches has motivated the field to develop new methods.”*

*Subsequently, there have been other methods developed to predict 3D chromatin interactions, and each method is suited to a different question because the inputs and outputs of each method are different from each other. We describe these methods in the introduction as follows:*

*“Recently, the convolutional neural network framework was adapted to predict Hi-C contact matrices from 1-dimentional sequence data in a method called “Akita” [24]. There are other methods that predict Hi-C-like data and chromatin interactions, namely DeepTACT [25], SEPT [26], and DeepC [27]. A detailed comparison and description of these methods is discussed in Supplementary Table 1.*

*CTCF-associated genome folding patterns can be observed in the prediction results of Akita, suggesting the importance of CTCF in regulating chromatin interactions. In addition, prediction results can recapture the differences in genome folding between a normal and genetically altered cell lines, indicating that machine learning framework can predict different genome folding profiles given different input DNA sequences.*

*However, there are several limitations to these methods. First, Akita and DeepC only performs predictions with limited sequence regions (in the case of Akita, this is 1 Mb), thus long-range chromatin interactions cannot be predicted and genome-wide chromatin interactions cannot be obtained with these methods. Second, it is unclear whether ChIA-PET data can be predicted, as DeepTACT predicts promoter capture Hi-C data [25], and Akita, DeepC and SEPT are restricted to Hi-C data. Third, none of these methods have been tested for their abilities to predict chromatin interactions de novo in patient cancer samples.”*

*Additionally, we discussed these methods in the results section of the paper, and further added a paragraph at the end to summarize the differences. We also included Supplementary Figure 7 to illustrate the differences between the different methods and provide a method for researchers to select the optimum machine learning method for their needs. The discussion is as follows:*

*“Other methods that predict Hi-C-like data and chromatin interactions are available, namely DeepTACT [25], SEPT [26], Akita [44], and DeepC [27]. However, except for SEPT, the other three machine learning methods are very different from ChINN in terms of the data. For example, DeepTACT uses promoter-capture Hi-C input data, which is quite different from the use of Hi-C and ChIA-PET input data. For Akita and DeepC, their output consists of Hi-C contact matrices on lists of user-specified genomic regions, while our output is chromatin loops and probabilities of interaction across the whole genome. As a consequence, we cannot call loops from these partial Hi-C matrices of Akita and DeepC, because we would not know the background genomic interaction distribution. Therefore, direct comparison between ChINN output and Akita/DeepC output is not possible.*

*As for the SEPT, following its pipeline, we extended the input sequences or cut to 3 kb or 2 kb flanking regions from the center. But SEPT performs worse as compared with ChINN on our dataset (AUPRC=0.0016 evaluated on K562 Hi-C test datasets with HeLaS3 as source data), as the sequences in our dataset are longer than these input sequences. Therefore, cutting the sequence to 3 kb or 2 kb according to what SEPT does would not let the model learn much useful information.*

*We concluded that each method is designed to investigate different questions, and in Supplementary Figure 7, we summarized the decision making process for researchers who wish to use the different methods. For example, if the researcher is interested in promoter-promoter or promoter-enhancer interactions identified by PCHi-C (Promoter Capture Hi-C), they should use DeepTACT. If the researcher is interested in an output that is shown as a Hi-C heatmap, they should use Akita. If the researcher is interested to look at general chromatin interactions predicted from Hi-C data, or RNA Pol II and CTCF chromatin interactions predicted from ChIA-PET data, they should use ChINN. ChINN is the only machine learning method currently available for predicting Hi-C and ChIA-PET chromatin interactions from sequences with outputs specified as open chromatin associated chromatin interactions instead of Hi-C matrices.”*

C. “the authors … do not show enough bright spot of this work.”

*Response 4C. We revised the introduction of our paper to highlight several bright spots of our work, as follows:*

*“To overcome these challenges, in this study, we investigated the possibility of utilizing DNA sequence features to predict chromatin interactions between open chromatin regions, regardless of distance between them. Our study has several advantages. First, we demonstrated that open chromatin interactions can be predicted accurately from functional genomic data at the resolutions of the experimental techniques.*

*Second, we then developed a novel method, called Chromatin Interaction Neural Network (ChINN) to predict open chromatin interactions from DNA sequences. This model has been developed for RNA Polymerase II (RNA Pol II) ChIA-PET interactions, CTCF ChIA-PET interactions and Hi-C interactions, overcoming previous limitations in terms of data input. Moreover, ChINN is able to identify open chromatin interactions in a genome-wide manner, overcoming the limitations of previous methods which were restricted to specific genomic regions.*

*Third, we extensively validated our method. ChINN was able to identify convergent CTCF motifs, AP-1 transcription family member motifs such as FOS, and other transcription factors such as MYC as being important in predicting chromatin interactions. Moreover, we further applied our model to a set of 6 newly generated chronic lymphocytic leukemia samples, which showed patient-specific chromatin interactions. We were able to validate predicted interactions by Hi-C and 4C. The models were then applied to a cohort of previously published 84 chronic lymphocytic leukemia (CLL) samples [28]. Thus, we demonstrated the prediction power of our method in practice.*

*Fourth, we used ChINN to characterize the levels of open chromatin interaction heterogeneity in patient samples. While we found that many chromatin interactions are ubiquitous, we also found widespread evidence for patient-specific open chromatin interactions, and open chromatin interactions that were different in different subtypes of CLL.*

*Taken together, our results indicate both functional genomics models and ChINN can predict open chromatin interactions, and application of ChINN to cancer patient samples demonstrates widespread patient heterogeneity in chromatin interactions.”*

*Additionally, in the conclusion, we summarize the significance of our work, as follows: “A functional genomics approach is able to predict chromatin interactions. The ChINN framework is able to predict chromatin interactions from open chromatin regions in the human genome, using DNA sequences and distances as features. This framework can be applied in other cell lines or clinical samples given the knowledge of open chromatin regions, making it a useful tool to interrogate chromatin interactions when large-scale functional genomics acquisition is not applicable due to limited biological materials.”*

1. The strategy the authors used to generate the negative samples are not reasonable. Distance-matched negative samples should be generated from all possible combination of anchor pairs, and simultaneously satisfied the same distance distribution as positive pairs. Please check the strategy used by TargetFinder (Whalen et al, Nature Genetics, 2016). And the author didn’t answer the question that what proportion of negative samples are generated on a different chromosome, which should be the number of negative samples derived on a different chromosome divide the total number of negative samples.

*Response 5A. As the reviewer mentioned TargetFinder (Whalen et al, Nature Genetics, 2016), we would like to briefly discuss our previous correspondence with Nature Genetics (Cao et al, Nature Genetics, 2019) regarding TargetFinder and other enhancer-promoter prediction methods. We started this project by utilizing the TargetFinder datasets for development. Using the TargetFinder datasets, we achieved performances similar to TargetFinder by using only transcription factor binding motifs in the genomic regions between the enhancer-promoter pairs in their datasets. However, we later found out that by using random motifs, similar performances could also be achieved.*

*We identified that these high performances were caused by large overlaps in the samples between training and test sets. The lab of Michael Beer also made similar observations in a perspective (Xi and Beer, PLoS Computational Biology, 2018). As they say in their paper “Cross-fold validation schemes which do not correctly separate these feature sharing enhancer-promoter pairs into one test set report high accuracy, which is actually arising from high training set accuracy and a failure to properly evaluate generalization performance.”*

*When we used chromosome-based splitting of training and test data, the performance of TargetFinder was close to random guesses. From that study, we concluded that to properly evaluate a machine learning method in genomics, the samples should always be split by chromosomes (Cao et al, Nature Genetics, 2019).*

*Our sampling approach is similar to TargetFinder for the distance-matched datasets. In general, like TargetFinder, for each positive sample, we sampled 5 negative samples that have similar distance as the positive sample. We require the 5 negative samples to be on the same chromosome as the positive sample. This is to ensure that when we split the data into training, validation and testing sets based on chromosomes, the positive to negative ratios are roughly consistent in the three sets of data.*

*In this study, we split the model development into multiple steps: 1) train the convolutional neural network using the distance-matched datasets; 2) train a classifier using the extended datasets; and 3) develop the final classifier from samples generated from open chromatin regions. It should be noted that even though the indirectly connected anchor pairs were excluded from the distance-matched datasets, they were all included in the extended datasets in step 2. The performance at step 2 are shown in Supplementary Figure 3. We adopted this step-by-step approach to allow the convolutional neural network to learn robust features that can discern interacting pairs from non-interacting pairs. Indirectly connected anchor pairs might represent true interactions that are missed by ChIA-PET, including them in the first step could confound the training process of the convolutional neural network. Thus, they were only added back in step 2.*

*In addition, when we say “a negative sample from a different chromosome”, it refers to a pair with both anchors coming from that chromosome. The following table (included as Supplementary Table 10) shows the negative-to-positive ratios of the distance-matched datasets for each chromosome. The minimum negative-to-positive ratio is 4.4 at chromosome 19 of GM12878 Polr2a dataset. The maximum negative-to-positve ratio is 5.51 at chromosome 13 of K562 Polr2a dataset.*

“And the author didn’t answer the question that what proportion of negative samples are generated on a different chromosome, which should be the number of negative samples derived on a different chromosome divide the total number of negative samples.”

*Response 5B. We apologize for the omission. We have now included a Supplementary Table 10 with the data.*

*Supplementary Table 10: Negative-to-positive ratios of samples on each chromosome of distance-matched datasets.*

2. I didn’t catch the necessity between the first two sections, i.e. the “functional genomic model” and the sequence model (ChINN). If the author want the “functional genomic model” to work as a baseline model, they may just put it in the second section and compare the performance of these two models to show the advantage of ChINN.

*Response 6. As we mentioned above in the discussion of our correspondence with Nature Genetics regarding TargetFinder, TargetFinder’s performance can be purely attributed to the overlapping between training and testing data. In our testing using a cross-chromosome approach, TargetFinder is not predictive at all. Other machine learning methods at that time also had similar issues. Thus, the literature did not support the notion that chromatin interactions can be predicted.*

*Our logic of developing sequence-based method consists of two parts: 1) chromatin interactions can be predicted from function genomic data, represented by methods such as TargetFinder and JEME (Cao et al, Nature Genetics, 2017)); 2) Transcription factor binding can be predicted from sequence, represented by methods such as DeepSea (Zhou et al, Nature Methods, 2015) and DeepBind (Alipanahi et al, Nature Biotech, 2015). Sequence -> TF binding -> chromatin interaction is the logic we were following. If TargetFinder and JEME were predictive only because of overfitting, our logic would be broken. Thus, this first section forms the basis of the sequence-based work.*

*In the revised manuscript, we explain the rationale for the first section “Open chromatin interactions can be predicted from functional genomic features” as: “In light of Xi et al. [22] and our previous study [23] showing that the existing prediction methods have exaggerated performances, we first tried to demonstrate that chromatin interactions could be predicted from functional genomic data”. And in the second section, we connect up the two sections by saying “In our previous section, we showed that open chromatin interactions can be predicted from functional genomics data, which consists of transcription factor data. As transcription factor binding can be predicted from sequences as shown by methods such as DeepSea (Zhou et al, Nature Methods, 2015) and DeepBind (Alipanahi et al, Nature Biotech, 2015), we reasoned that open chromatin interactions can be predicted from DNA sequences. Consequently, we went on to explore whether open chromatin interactions can be predicted from DNA sequences.”*

3. The model trained using sequence features of CTCF ChIA-PET data performs better than the models trained using functional genomics data from CTCF ChIA-PET in the cross-sample prediction. The author claim that this is because the low resolution of ChIP-seq data and that the ChIP-seq peaks cannot tell the orientation of the CTCF binding, while sequence can tell the direction of CTCF motif. If this is the reason, why the model trained using sequence features of Pol2 ChIA-PET data performs worse than the models trained using functional genomics data?

*Response 7. CTCF binding motifs are directional. Chromatin interactions associated with CTCF showed particular patterns in terms of direction of the two anchors. On the other hand, Pol2 binding sites are usually GC-rich, and does not have the pattern of directionality. Therefore, we would expect that the model trained with sequence features of Pol2 ChIA-PET data will not perform as well as the models trained with functional genomics data, because Pol2 sites do not have strong motifs with directionality unlike CTCF.*

4. The authors said they have corrected the way of selecting the cut-offs for F1 scores. Please add the corresponding experiments and the results. Even the threshold may remain the same, the authors should use numbers and results to show it in the response letter.

*Response 8. We thank the reviewer for his/her suggestion. We implemented the description of experiments and the results at the end of Section 2.13 in Supplementary Materials as*

*“We obtained the predicted probabilities scores for validation datasets, the samples with scores larger or equal than the cut-off value were assigned as predicted to be positive; the samples with scores smaller than the cut-off value were assigned as predicted to be negative. The F1 scores were calculated using Python function sklearn.metrics.f1_score with different selection of cut-off values (Supplementary Table 9)”.*

*Supplementary Table 9: F1-score calculated with different cut-off values from pairs of open chromatin regions in GM12878 cells.*

*Cut-off value 0.1 0.15 0.18 0.2 0.22 0.25 0.3 0.4 0.5*

*GM12878_CTCF 0.4439 0.4798 0.4909 0.4956 0.5027 0.4998 0.4987 0.4715 0.4312*

*GM12878_Polr2a 0.3479 0.3865 0.4005 0.3990 0.3983 0.3954 0.3866 0.3407 0.2832*

5. There have been many methods developed to predict 3D chromatin interactions using sequences since five years ago, the authors did not show enough bright spot of this work. I still think the authors should find a way to compare ChINN with at least three of the state-of-art methods to show the advantage of ChINN. Otherwise, when researchers need to predict chromatin interactions, they can just choose other existing methods rather than ChINN.

*Response 10. We discussed the comparison with old methods as well as the “bright spots” in earlier sections of this response (response 4B and 4C respectively). As our method has different inputs, goals, and outputs as compared with other existing methods, it is difficult to directly compare the methods on the exact same input data. To compare between these different methods, we provided Supplementary Table 1 which describes how each method differs from each other.*

*In order to explain how researchers should select which method to predict chromatin interactions, we discussed at the end of “Predicting chromatin interactions from open chromatin regions”:*

*“We concluded that each method is designed to investigate different questions, and in Supplementary Figure 7, we summarized the decision making process for researchers who wish to use the different methods. For example, if the researcher is interested in promoter-promoter or promoter-enhancer interactions identified by PCHi-C (Promoter Capture Hi-C), they should use DeepTACT. If the researcher is interested in an output that is shown as a Hi-C heatmap, they should use Akita. If the researcher is interested to look at RNA Pol II or CTCF chromatin interactions predicted from ChIA-PET and Hi-C data, they should use ChINN”.*

*We also concluded by describing the major advantage of ChINN: “ChINN is the only machine learning method currently available for predicting Hi-C and ChIA-PET chromatin interactions from sequences with outputs specified as open chromatin associated chromatin interactions instead of Hi-C matrices.”*

*Supplementary Figure 7: A diagram of user demands among different chromatin interactions prediction using sequences.*

6. Usually, the interaction predicted using cross-sample model should be more conserved and shows less cell-type specificity. Then, how can the authors study the sample heterogeneity based the interactions predicted using cross-sample model? The discussion now did not answer this question.

*Response 11. We responded to this question in response 4A.*

**Third round of review**

**Reviewer 1**

Comments to the Author

The authors have deposited the source code of ChINN with Github this time. But it's still difficult to reproduce and apply ChINN with available code. As a computational method developed for predicting chromatin interactions from DNA sequences, ChINN is supposed to be easily applied to study related biological problems. My suggestion is therefore to optimize the code and provide more detailed user guide.

Below, I outline my suggestions:

1. The source code should be organized more carefully.

1) The 'DATASET.md' file for preparing datasets in correct format is not available in the repository and the command for getting source code ($ git clone https://github.com/mjflab/chinn/tree/nscc; $ cd pytext) is expired.

2) In 'Data generation and preprocessing' part, 'process_pos.sh' and 'pipe.sh' files for preprocessing data are not available in current repository.

2. A more detailed and user-friendly instruction should be provided.

1) Authors can provide sample data and show how to use this data for training, testing and predicting using ChINN.

2) How to apply ChINN with data in different format or sequencing method to predict chromatin interaction and whether ChINN should be re-trained in different situation should be described more clearly.

3) Describing the demo datasets from 'Dataset.zip' clearer would help users reproduce this work.

4) The arguments of each function should be described more clearly. For example, what dose model_prefix, the positional arguments of test.py, refer to and how to choose or get it?

3. The code might be retested to confirm that users can successfully apply ChINN.

**Authors Response**

**Point-by-point responses to the reviewers’ comments:**

The authors have deposited the source code of ChINN with Github this time. But it's still difficult to reproduce and apply ChINN with available code. As a computational method developed for predicting chromatin interactions from DNA sequences, ChINN is supposed to be easily applied to study related biological problems. My suggestion is therefore to optimize the code and provide more detailed user guide.

Below, I outline my suggestions:

1. The source code should be organized more carefully.

1) The 'DATASET.md' file for preparing datasets in correct format is not available in the repository and the command for getting source code ($ git clone https://github.com/mjflab/chinn/tree/nscc; $ cd pytext) is expired.

2) In 'Data generation and preprocessing' part, 'process_pos.sh' and 'pipe.sh' files for preprocessing data are not available in current repository.

*Reply: We have updated our github repository to include the missing files mentioned by the reviewer.*

2. A more detailed and user-friendly instruction should be provided.

1) Authors can provide sample data and show how to use this data for training, testing and predicting using ChINN.

*Reply: We have provided a K562 dataset as a sample dataset in the github repository.*

2) How to apply ChINN with data in different format or sequencing method to predict chromatin interaction and whether ChINN should be re-trained in different situation should be described more clearly.

*Reply: We have updated the documentation in the github repository on how to apply ChINN in other dataset.*

3) Describing the demo datasets from 'Dataset.zip' clearer would help users reproduce this work.

*Reply:* We have replaced the demo datasets with the sample datasets. We also provided a note file that contains a description of the sample datasets.

4) The arguments of each function should be described more clearly. For example, what dose model_prefix, the positional arguments of test.py, refer to and how to choose or get it?

*Reply: We have updated our documentation to include the help message on the arguments of the functions in github repository.*

3. The code might be retested to confirm that users can successfully apply ChINN.

*Reply: We have retested and confirm that ChINN can be applied following the guideline in the github repository.*
